# Mn(III)–O–Ce(IV) as a Surrogate for Highly Reactive Non-Heme Mn(IV)=O Species Supported by Benzimidazole-Based Ligand

**DOI:** 10.1021/acs.inorgchem.6c02577

**Published:** 2026-06-09

**Authors:** Sikha Gupta, Parkhi Sharma, Lucia Velasco, Divya Lakshmi Hareendran, Markell J. A. Lomax, Asterios Charisiadis, Khyati Jain, Anagha Puthiyadath, Timothy A. Jackson, Sharath Chandra Mallojjala, Dooshaye Moonshiram, Apparao Draksharapu

**Affiliations:** † Southern Laboratories-208A, Department of Chemistry, 30077 Indian Institute of Technology Kanpur, Kanpur 208016, India; ‡ Instituto de Ciencia de Materiales de Madrid, 16379 Consejo Superior de Investigaciones Científicas, Madrid 28049, Spain; § Departamento de Química Física, Universidad Complutense de Madrid, Avenida Complutense s/n, Madrid 28040, Spain; ∥ Department of Chemistry and Center for Environmentally Beneficial Catalysis, 4202 The University of Kansas, Lawrence, Kansas 66045, United States; ⊥ Department of Chemistry, Binghamton University, Binghamton, New York 13850, United States

## Abstract

The interaction of high-valent manganese–oxo species with Ca^2+^ plays a paramount role in the catalytic water oxidation reaction within the oxygen-evolving complex of photosystem II. In artificial systems, redox-active Lewis acids like Ce^4+^ have emerged as promising components when coordinated to metal–oxo complexes. Herein, we report the synthesis, detailed characterization, and reactivity of two novel complexes (L_5_)­Mn^III^–O–Ce^IV^ (**2B/2M**, L_5_ = BnTBEN = *N* ^1^-benzyl-*N* ^1^,*N* ^2^,*N* ^2^-tris­((1-methyl-1*H*-benzo­[*d*]­imidazole-2-yl)­methyl)­ethane-1,2-diamine and MeTBEN = *N* ^1^-methyl-*N* ^1^,*N* ^2^,*N* ^2^-tris­((1-methyl-1*H*-benzo­[*d*]­imidazole-2-yl)­methyl)­ethane-1,2-diamine) in MeCN at ambient conditions. The incorporation of benzimidazole ligands enhances their reactivity in both oxygen atom transfer and hydrogen atom transfer reactions relative to pyridine analogs. Remarkably, adding HClO_4_ or Sc^III^(OTf)_3_ to **2B/2M** triggers the formation of highly reactive [(L_5_)­Mn^IV^=O]^2+^ species (**3B/3M**). These findings are in sharp contrast with our previous report, where the reaction of (BnTPEN)­Mn^III^–O–Ce^IV^ with HClO_4_ and Sc^III^(OTf)_3_ resulted in the formation of (BnTPEN)­Mn^IV^–OH and (BnTPEN)­Mn^IV^–O–Sc^III^, respectively. These newly formed Mn^IV^=O intermediates (**3B/3M**) were extensively characterized by various spectroscopies and ESI-MS. Notably, [(L_5_)­Mn^IV^=O]^2+^ exhibits superior reactivity toward thioanisole derivatives at −40 °C in MeCN, surpassing the known nonheme high-valent Mn^IV^=O species reported thus far. This study advances understanding of ligand effects on high-valent manganese–oxo species and highlights their potential in oxidation catalysis.

## Graphical abstract

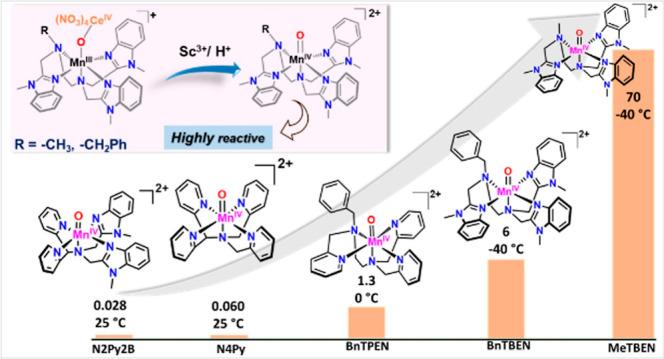

## Introduction

High-valent Mn complexes, particularly Mn–oxo species, have garnered significant attention as crucial intermediates in the oxygen-evolving complex (OEC) of photosystem II (PS-II).^1^ It was hypothesized that a transient Mn^V^–oxo intermediate is vital for the O–O bond-forming step preceding O_2_ evolution at the OEC in PS-II.^2,3^ Despite the extensive research conducted by numerous groups worldwide, the mechanism underlying the formation of the O–O bond remains elusive.^4,5^ High-valent Mn–oxo species have emerged as a focal point within the bioinorganic chemistry community, owing to their remarkable and versatile chemical reactivity. Synthetic high-valent Mn^IV/V^=O complexes bearing heme and nonheme ligands serve as key intermediates in various reactions, including C–H bond activation and the oxidation of olefins, alcohols, and sulfides.^6^ Numerous factors influencing the reactivity of Mn–oxo complexes have been observed in these studies, including geometric and electronic parameters and the basicity of the oxo moiety. To understand how the local coordination sphere influences the reactivity of Mn^IV^=O species in oxygen atom transfer (OAT) reactions, Jackson and co-workers modified the N4Py ligand framework and introduced equatorial ligand perturbations.^7^ Two of the four pyridyl groups of N4Py (*N*,*N*-bis­(2-pyridylmethyl)-*N*-bis­(2-pyridyl)­methylamine) were substituted with quinolinyl (N2Py2Q = *N*,*N*-bis­(2-quinolylmethyl)-*N*-bis­(2-pyridyl)­methylamine), benzimidazolyl (N2Py2B = *N*-bis­(1-methyl-2-benzimidazolyl)­methyl-*N*-(bis-2-pyridylmethyl)­amine), and 3,4-dimethyl-5-methoxypyridyl (^DMM^N4Py = *N*,*N*-bis­((4-methoxy-3,5-dimethylpyridin-2-yl)­methyl)-1,1-di­(pyridin-2-yl)­methanamine) moieties.^8–10^ It was demonstrated that the average Mn–Nequatorial distances found in the crystal structures of the corresponding Mn^II^ complexes correlated with changes in reaction rates for these Mn^IV^=O complexes. This series of compounds exhibited the following tendencies in the second-order rate constant of thioanisole oxidation at 25 °C: [(N2Py2Q)­Mn^IV^=O]^2+^ (9.2 M^–1^ s^–1^) > [(N4Py)­Mn^IV^=O]^2+^ (0.06 M^–1^ s^–1^) > [(N2Py2B)­Mn^IV^=O]^2+^ (0.028 M^–1^ s^–1^) > [(^DMM^N4Py)­Mn^IV^=O]^2+^(0.0023 M^–1^ s^–1^). Higher average Mn–N_equatorial_ distances corresponded to faster substrate oxidation rates.^10^

Additionally, the reactivity of high-valent Mn–oxo complexes can be augmented by the binding of redox-inactive Lewis acids (i.e., Sc^III^(OTf)_3_, Ca^II^(OTf)_2_, Mg^II^(OTf)_2_, Zn^II^(OTf)_2_, Al^III^(OTf)_3_, Y^III^(OTf)_3_, Lu^III^(OTf)_3_), or the addition of Brønsted acids (i.e., HClO_4_ and HOTf), rendering them exceedingly potent oxidants.^11–15^ The dramatic increase in reactivity was attributed to the shift in the Mn^IV/III^ reduction potential to a more positive value in the presence of Lewis and Brønsted acids.^13,14^ On the other hand, the redox-active metal ion like Ce^IV^, bound to [(DPAQ)­Mn^IV^=O]^2+^ (DPAQ = 2-[bis­(pyridin-2-ylmethyl)]­amino-*N*-quinolin-8-ylacetamide)) was found to be a more potent oxidant in electron transfer and OAT reactions than Mn^IV^=O complexes binding redox-inactive metal ions.^15^ Recently, our group reported an alternative method to generate (BnTPEN)­Mn^IV^–O–Sc^III^ and (BnTPEN)­Mn^IV^–OH from a unique precursor [(BnTPEN)­Mn^III^–O–Ce^IV^(NO_3_)_4_]^+^ (**4**) species.^16^ Despite being in the Mn^III^ state, the newly generated species **4** exhibited the capability to perform OAT and HAT reactions.^16^

This study focuses on assessing whether modifying the ligand framework, from pyridine-based to benzimidazole-based structures, can modulate the reactivity of [(L_5_)­Mn^III^–O–Ce^IV^(NO_3_)_4_]^+^ complexes, where L_5_ denotes a neutral pentadentate ligand. Two nonheme manganese complexes, [(BnTBEN)­Mn^III^–O–Ce^IV^(NO_3_)_4_]^+^ (**2B**) and [(MeTBEN)­Mn^III^–O–Ce^IV^(NO_3_)_4_]^+^ (**2M**), (where BnTBEN = *N* ^1^-benzyl-*N* ^1^,*N* ^2^,*N* ^2^-tris­((1-methyl-1*H*-benzo­[*d*]­imidazole-2-yl)­methyl)­ethane-1,2-diamine and MeTBEN = *N* ^1^-methyl-*N* ^1^,*N* ^2^,*N* ^2^-tris­((1-methyl-1*H*-benzo­[*d*]­imidazole-2-yl)­methyl)­ethane-1,2-diamine) were synthesized and rigorously characterized using spectroscopic techniques, including UV–vis, EPR, XAS, NMR, and ESI-MS. The reactivity of these complexes toward electron transfer (ET), oxygen atom transfer (OAT), and hydrogen atom transfer (HAT) processes was systematically examined. Both **2B** and **2M** displayed enhanced reactivity relative to the pyridine-based [(BnTPEN)­Mn^III^–O–Ce^IV^(NO_3_)_4_]^+^ (**4**) analogue. Upon treatment with HClO_4_ or Sc^III^(OTf)_3_, **2B** and **2M** yielded [(L_5_)­Mn^IV^=O]^2+^ (**3B/3M**), which were similarly characterized by various spectroscopic methods. The electron transfer properties of **3B/3M** toward ferrocene and its derivatives were also studied. Notably, **3B** and **3M** showed pronounced reactivity toward thioanisole in acetonitrile at −40 °C, with measured second-order rate constants (*k*_2_) of 6 M^–1^ s^–1^ and 70 M^–1^ s^–1^, respectively. To the best of our knowledge, the [(L_5_)­Mn^IV^=O]^2+^ complexes generated in this investigation represent the most reactive nonheme Mn^IV^=O species reported for thioanisole oxidation to date (Scheme 1).

**Scheme 1:**
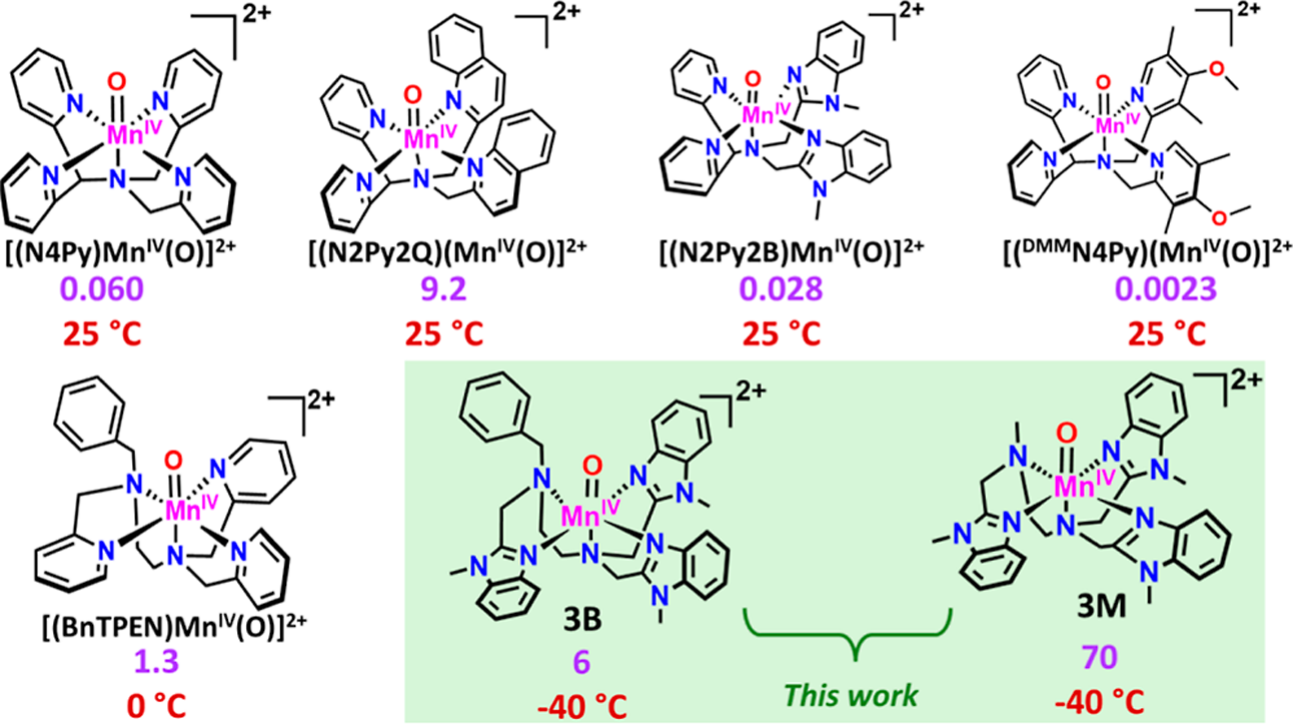
Second-Order Rate Constants (in M^–1^ s^–1^) for Thioanisole Oxidation Were Determined at Various Temperatures Using Mn^IV^=O Complexes Bearing Neutral Pentadentate Ligand Frameworks^8–10^

## Results and Discussions

Equimolar amounts of [Mn^II^(H_2_O)_6_]­(ClO_4_)_2_ and either BnTBEN or MeTBEN were combined in acetonitrile, forming a colorless solution. Introduction of ether to this solution via vapor diffusion resulted in the isolation of white crystalline products, denoted as **1B** for BnTBEN ligand and **1M** for MeTBEN. Structural analysis by single-crystal X-ray diffraction revealed that **1B** adopts a distorted octahedral geometry, with average Mn–N_bzim_ and Mn–N_amine_ bond lengths of 2.21 Å and 2.41 Å, respectively (Table S2). In **1B**, five nitrogen atoms from the BnTBEN ligand coordinate to the Mn­(II) center, while the sixth site is occupied by an oxygen atom from perchlorate (Figure 1). The crystallographic study of **1M** similarly demonstrated a hexacoordinated Mn­(II) center. Here, five nitrogen atoms from the MeTBEN ligand and a nitrogen from acetonitrile fulfill the coordination sphere, with the structure showing a distorted octahedral geometry and average Mn–N_bzim_ and Mn–N_amine_ distances of 2.18 Å and 2.40 Å, respectively (Figure 1 and Table S2). Comparison with previously published data for [(BnTPEN)­Mn^II^(OClO_3_)]^+^ indicated that the average Mn–N_amine_ distance in the current complexes is approximately 0.10 Å longer,^16^ potentially reflecting the geometric constraints imparted by the benzimidazolyl rings (five-membered) versus the pyridyl rings (six-membered), which may influence bond angles and thus bond lengths.

**Figure 1:**
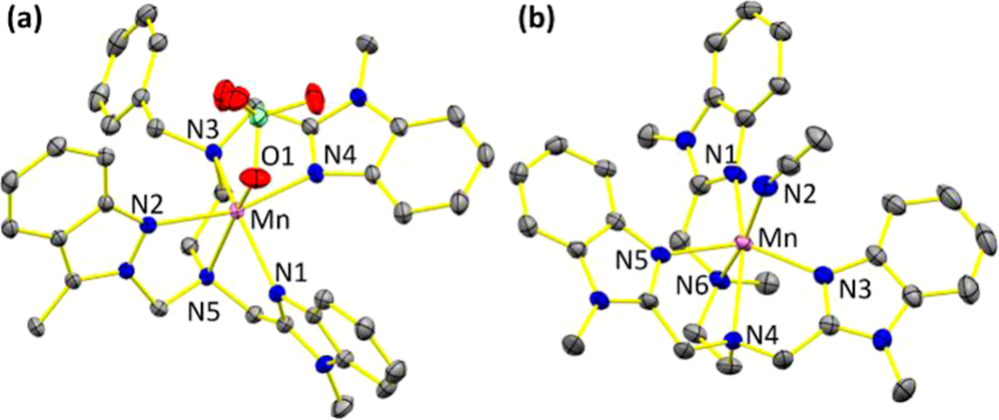
X-ray crystal structures of **1B** (a) and **1M** (b) with 50% ellipsoid probability level. Hydrogen atoms, solvent molecules, and ClO_4_^–^ counteranions were omitted for clarity. CCDC 2367385 and 2369687.

Treatment of the acetonitrile solutions of **1B** and **1M** with 4 equiv of ceric ammonium nitrate (CAN) and 20 μL of water at room temperature resulted in the formation of orange-colored species, designated **2B** and **2M**, respectively. Both complexes exhibited a shoulder absorption band near 550 nm (Figures 2a and S1a). A CAN concentration-dependent study confirmed that 4 equiv were sufficient to achieve maximal formation of **2B** and **2M** (Figures 2b and S1b). The UV–vis spectral features of **2B/2M** closely resembled those of the previously reported (BnTPEN)­Mn^III^–O–Ce^IV^ (**4**) complex, which showed an absorption band centered at 530 nm.^16^ Electrospray ionization mass spectrometry (ESI-MS) of **2B** displayed a prominent ion peak at *m*/*z* 1041.11, which shifted to *m*/*z* 1043.11 upon incorporation of H_2_^18^O (Figures 2c, S2, and S3). Similarly, **2M** exhibited a signal at *m*/*z* 965.08, which shifted to 967.08 with H_2_^18^O labeling (Figure S1c). The isotopic distributions and masses correspond well to the formulations [(BnTBEN)­Mn^III^(O)­Ce^IV^(NO_3_)_4_]^+^ for **2B** and [(MeTBEN)­Mn^III^(O)­Ce^IV^(NO_3_)_4_]^+^ for **2M**, respectively. Attempts to record resonance Raman spectra were unsuccessful due to fluorescence interference from these complexes. ^1^H NMR spectra of **2B** and **2M** in CD_3_CN at room temperature exhibited well-resolved paramagnetically shifted signals over a range of approximately −20 to 40 ppm (Figures S4 and S5), consistent with the Mn^III^ oxidation state, and similar to reported data for complex **4**.^16^

**Figure 2:**
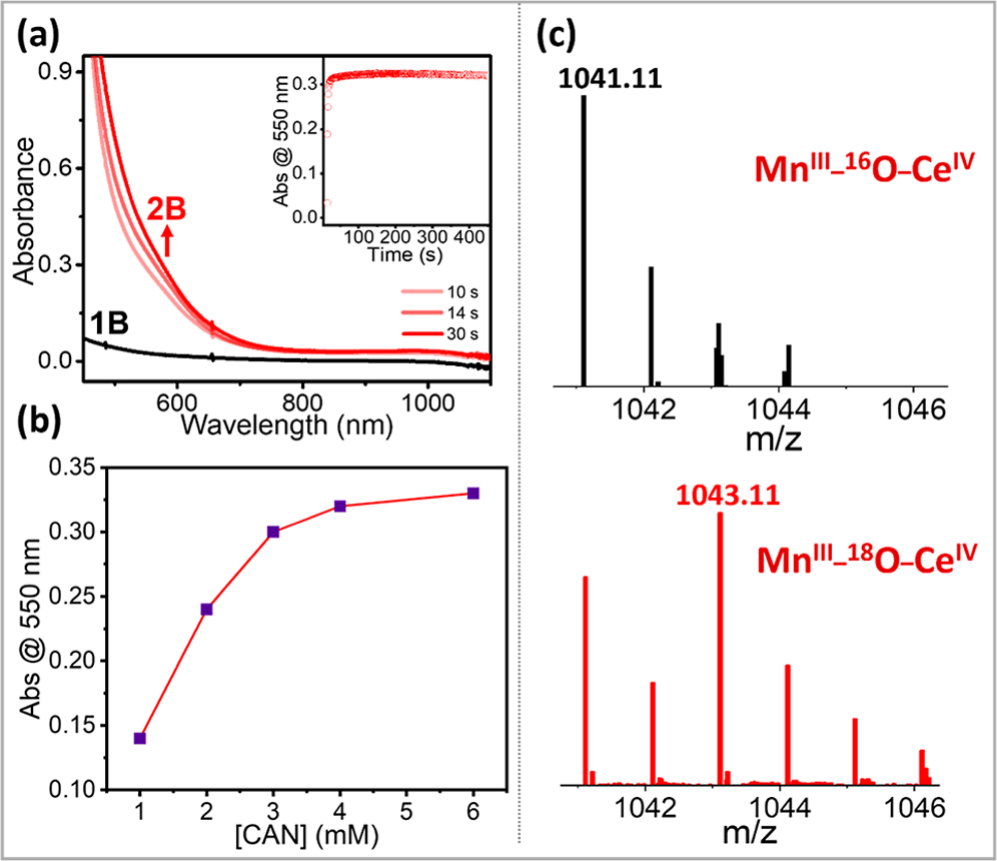
(a) UV–vis absorption spectral changes showing the formation of **2B** (red) after adding 20 μL H_2_O and 4 equiv of CAN to 1 mM **1B** (black) in MeCN at 25 °C. The inset depicts the plot of absorbance at 550 nm vs time. (b) Absorbance vs [CAN] plot depicting the formation of 550 nm species in MeCN at 25 °C. (c) ESI-MS analysis for species **2B** (black) and ^**18**^ **O-2B** (red). Condition to generate **2B** and ^**18**^ **O-2B**: 1 mM **1B** + 20 μL H_2_O/H_2_^18^O + 4 equiv of CAN at 25 °C in MeCN.

From our previous studies, it was apparent that at lower temperatures, Mn­(III)–O–Ce­(IV) converts to Mn­(IV) species.^16^ We have carried out X-ray absorption near-edge structure (XANES) and Extended X-ray absorption fine structure analysis (EXAFS) analyses of complexes **1B**, **2B**, **1M**, and **2M** to gain insight into their coordination behaviors, electronics, and structural conformations. The complexes were kept at 15 K in a He atmosphere at ambient pressure and recorded as fluorescence excitation spectra. **1B**, **2B**, **1M**, and **2M** all display a rising edge from 6544 to ∼6554 eV, corresponding to the 1s → 4p electron transition (Figures 3a and S6).^7^ Interestingly, both **2B** and **2M** exhibit a clear energy shift of 2.74 eV from 6549.13 to 6551.87 eV compared to **1B** and **1M** (Figures 3a and S6a), reflecting the higher ionization energy required to eject a core 1s electron from a more positively charged ion. The observed energy shift of more than 2 eV illustrates the formation of a Mn^IV^ species at low temperatures for both **2B** and **2M**, as previously observed.^15,18–20^ The edge energies of **2B** and **2M** are in further close agreement with those observed for Mn^IV^ complexes.^15,21–25^ Thus, at 15 K, **2B/2M** are converted to a Mn^IV^ species, while at 298 K, they exist in the Mn­(III) state (vide supra). Interestingly, substantial changes are additionally observed in the pre-edge region of the absorption spectra of **1B/2B** and **1M/2M** at lower photon energies around 6545 eV (Figure 3a inset). The presence of pre-edge features corresponds to 1s to 3d quadrupole transitions and dipole excitations of the core electrons into the valence 3d states, hybridized with ligand p orbitals.^26–28^ **1B** and **1M** both exhibit a pre-edge feature at 6541.2 eV, while **2B** and **2M** show a more intense and positively shifted pre-edge at 6542.0 eV (Figure 3b). Time-dependent DFT (TD-DFT) simulations of the pre-edge XANES spectra were performed using ORCA^29^ with the B3LYP^30,31^ functional. Although the calculated intensities cannot be directly compared to experimental values (Figure 3b), the simulations confirm that **2B** and **2M** possess more intense pre-edge features than their respective parent complexes **1B** and **1M**, along with a pre-edge peak at 6542.0 eV, as elaborated above (Figure 3b). This closely aligns with the computationally predicted maxima at 6542.7 eV (Figure 3b), supporting the formation of a Mn^IV^ intermediate featuring an elongated Ce^IV^ scatterer.

**Figure 3:**
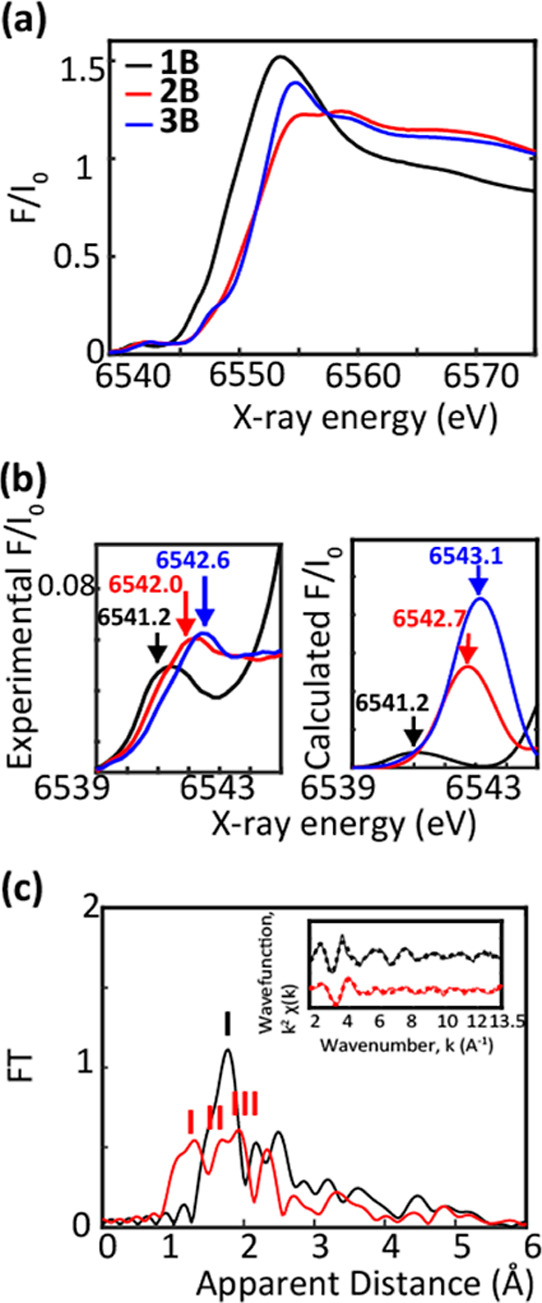
Structural parameters derived via XANES and EXAFS: Normalized Mn K-edge XANES spectra recorded at 15 K of (a) **1B**, **2B**, and **3B** (shown in black, red, and blue, respectively). (b) (Left) Zoom-in of the pre-edge region of **1B**, **2B**, and **3B**. (Right) TD-DFT XANES calculated pre-edges of three models generated from **1B**, namely a Mn^II^ complex coordinated to MeCN molecule (black), a Mn^IV^ oxidized species with a loosely coordinated Ce^IV^ (red), and a Mn^IV^=O intermediate (blue). (c) Fourier transforms of k^2^-weighted Mn EXAFS of **1B** and **2B** (in black and red, respectively). Inset shown in panel (c). *k* ^2^[χ­(*k*)]-weighted traces as a function of *k*, the photoelectron wavevector (solid lines) and fitted (dashed lines) of the Mn complexes. Experimental spectra were calculated for *k* values of 1.5–13.5 Å^–1^. Conditions for the preparation of **2B**: 5.5 mM **1B** in MeCN + 20 μL H_2_O + 4 equiv of CAN at 298 K. **3B**: 5.5 mM **1B** in MeCN + 20 μL H_2_O + 4 equiv of CAN + 20 equiv of HClO_4_ at 298 K.

The EXAFS spectral comparisons of complexes **1B** vs **2B** and **1M** vs **2M** are further shown in Figures 3c and S6c. A prominent peak **I** is observed in the EXAFS spectra of both **1B** and **1M**, corresponding to the averaged contribution of the Mn–N distances (black trace, Figures 3c, and S6c). The EXAFS fits for the extraction of the actual bond distances of all the studied complexes are shown in Figures 3c, S6c, and S7, and in Table S3. Analysis of the EXAFS spectra of **1B** and **1M** in solution yields 6 averaged Mn–N bond distances at 2.21 Å. The fit quality, however, considerably improves upon inclusion of 4 Mn–N bond distances at 2.23 Å and 2 elongated Mn–N distances at 2.54 Å (Table S3, Fit 1 vs 2, 3), revealing a distorted octahedral geometry and longer bond distances for the Mn–N_amine_ group as determined from both crystallography and DFT studies (Table S4). DFT optimization calculations were performed using the BP86 functional, as detailed in the Supporting Information (Table S4). The experimentally determined Mn–N EXAFS distances for **1B** vary within 0.06–0.09 Å from its DFT optimized geometry, while that for **1M** deviates within 0.05–0.07 Å. This demonstrates that theoretical methods, including geometry optimizations, can be reliably used to understand the structural conformations of the intermediate species **2B** and **2M** as discussed below.

The EXAFS spectrum of **2B** illustrates 3 peaks **I**, **II**, and **III** corresponding to the shortened Mn^IV^=O distance (peak **I**), and the Mn–N distances (peak **II**, **III**) as illustrated in Figure 3c, which can be best fitted with a shortened Mn–O bond distance at 1.70 Å, 2 Mn–N distances at 2.07 Å, and 3 Mn–N distances at 2.26 Å (Fit 6, Table S3, Figure S7), **2M** shows 2 peaks **I** and **II** (Figure S6c) matching once again with the shortened Mn^IV^=O distance of 1.70 Å and 5 averaged Mn–N bond distances at 2.13 Å (Fit 14, Table S3, Figure S7). The Mn–O distance extracted for both **2B** and **2M** corresponds very well with the presence of a Mn^IV^=O moiety as previously determined at 1.69 Å^15^ and with the DFT optimized structures of the Mn^IV^=O models (Section S2 in Supporting Information), which similarly yield a short Mn–O distance of 1.70 Å (Section S2 in Supporting Information). The average experimentally extracted Mn–N bond distances for **2B** and **2M** vary closely between 0.05 and 0.01 Å, respectively, compared to DFT-optimized geometries (Section S2 in Supporting Information). These close matches effectively rule out the formation of a Mn^III^–O–Ce^IV^ species at lower temperatures, which would be expected to exhibit a longer Mn–O bond distance of 1.74–1.75 Å and Mn–N bond distances averaging 2.20–2.21 Å as predicted computationally (Section S2 in Supporting Information). Instead, the data support the formation of a more oxidized Mn^IV^ species.

Upon treating intermediate **2B** with 20 equiv of HClO_4_ at room temperature in acetonitrile, a distinct absorption band at 510 nm, accompanied by a shoulder at 620 nm, denoted as **3B**, was detected (Figures 4a and S8). Species **3B** demonstrated moderate stability, decaying over time with a half-life of approximately 500 s. Notably, the addition of either 10 equiv of Sc^III^(OTf)_3_ or Yb^III^(OTf)_3_ to **2B** yielded identical absorption features to those observed for **3B** (Figure 4b). Similarly, introducing 20 equiv of HClO_4_ or 10 equiv of Sc^III^(OTf)_3_ to **2M** generated the **3M** complex, which exhibited an absorption band at 500 nm and a shoulder at 600 nm (Figures S9 and S10). Previous studies from our group have shown that adding HClO_4_ to **4** leads to the formation of (BnTPEN)­Mn^IV^–OH, which absorbs at 620 nm.^16^ The addition of Sc^III^(OTf)_3_ to **4** forms (BnTPEN)­Mn^IV^–O–Sc^III^, displaying a band at 650 nm.^16^ In contrast, the formation of similar absorption bands upon addition of either a Lewis or Brønsted acid to **2B/2M** signifies the generation of structurally analogous intermediates, most likely [(Bn/MeTBEN)­Mn^IV^=O]^2+^ species. ESI-MS measurements of **3B** showed a predominant peak at *m*/*z* 752.20, with the isotopic pattern consistent with the assignment as [(BnTBEN)­Mn­(O)­(ClO_4_)]^+^ (Figures 4c and S11). Introduction of isotope-labeled water (H_2_^18^O) produced a two-unit shift to *m*/*z* 754.20, confirming that water serves as the oxygen source and indicating **3B** as a Mn^IV^=O species (Figures 4c and S12). Similar analysis for **3M** revealed an *m*/*z* peak at 676.17, shifting to 678.17 in the presence of H_2_^18^O, suggesting **3M** as [(MeTBEN)­Mn­(O)­(ClO_4_)]^+^ (Figures S9, S13, and S14). EPR analysis of **3B/3M** performed at 77 K in MeCN revealed the signal at *g* = ∼4, suggesting the existence of an *S* = 3/2 species, confirming the oxidation state of Mn as +4 (Figures S15 and S16) (vide infra). Given that EPR signals for Mn^IV^ centers can show a strong temperature dependence, being most intense at the lowest temperatures, we also collected EPR data for a sample of **3B** at 9 K (Figure S17). This spectrum also showed a weak signal near *g* ∼4 and a prominent multiline signal near *g* ∼2. The multiline signal consists of six main lines, due to hyperfine coupling to the ^55^ Mn nucleus, with *a* = 9.1 mT. The *a* value is similar to that reported for [Mn^II^(OH_2_)_6_]^2+^.^32^ Spin quantification of the *g* ∼2 signal in **3B** shows that [Mn^II^(OH_2_)_6_]^2+^ corresponds to approximately 25% of the total Mn present in the sample.

**Figure 4:**
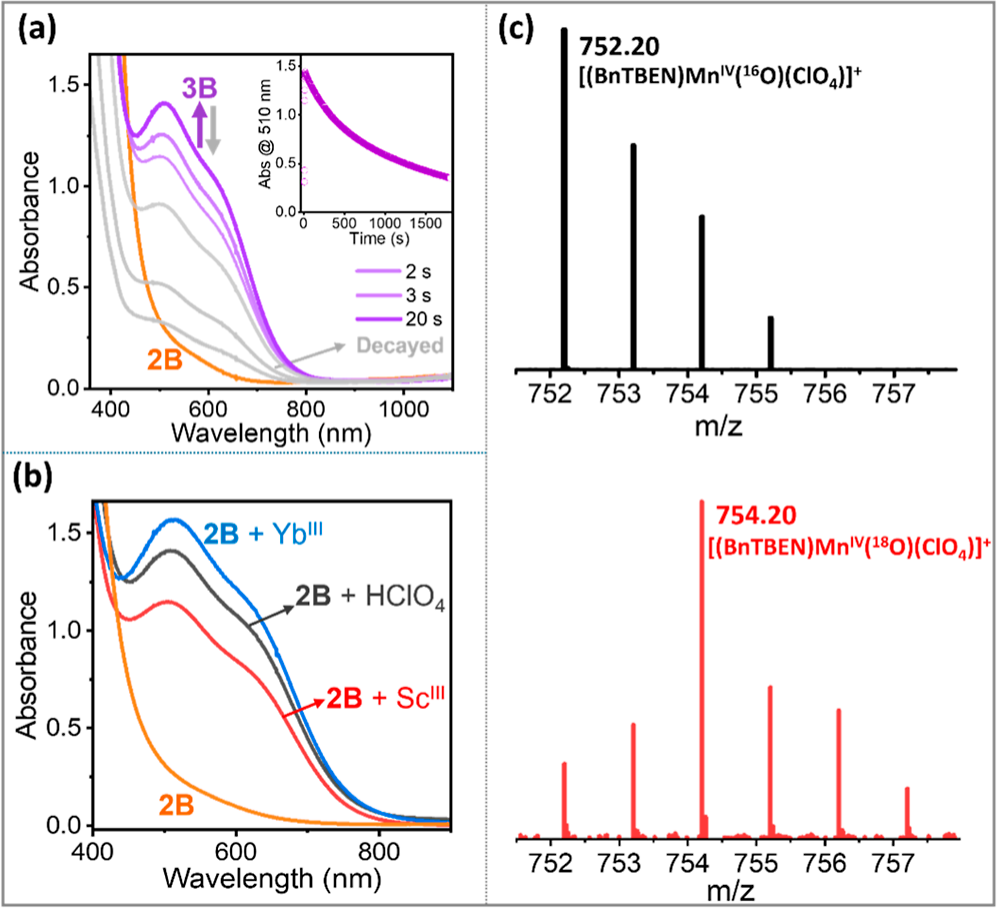
(a) UV–vis spectral changes showing the formation of **3B** after adding 20 equiv of HClO_4_ to **2B** in MeCN at 25 °C. (b) UV–vis spectra showing generation of **3B** upon adding 10 equiv of Sc^III^(OTf)_3_, 10 equiv of Yb^III^(OTf)_3_, and 20 equiv of HClO_4_. (c) ESI-MS analysis for species **3B** (black) and ^**18**^ **O-3B** (red). Note: Partial incorporation of ^18^O into species **3B** is noticed. Condition to generate **3B/** ^**18**^ **O-3B**:**2B/** ^**18**^ **O-2B** + 20 equiv of HClO_4_ in MeCN at 25 °C. Condition to generate **2B** and ^**18**^ **O-2B**: 0.5 mM **1B** + 20 μL H_2_O/H_2_^18^O + 4 equiv of CAN at 25 °C in MeCN.

XANES analysis on **3B** and **3M** further shows an energy shift of 2.74 eV from 6553.13 to 6555.87 eV, compared to **1B** and **1M**, respectively, which is similar to that observed in **2B** and **2M**, and confirms the presence of a Mn^IV^ species (Figures 3a, S6a).^15,18–20,33^ Furthermore, the pre-edge features of **3B** and **3M** exhibit higher intensities than those of **2B** and **2M** (Figures 3b, and S6b inset), with their peaks shifted to 6542.6 eV for **3B** and 6542.2 eV for **3M**. These values are in close agreement with the computationally predicted pre-edge positions of 6543.1 eV for both **3B** and **3M**, which are consistent with a Mn­(IV)=O structural motif (Figures 3b and S6b), thereby supporting the observed spectral trends. In contrast, the calculated pre-edge for a Mn­(IV)–OH motif exhibits a less intense maximum relative to **2B** and **2M**, as illustrated in Figure S18.

The EXAFS spectra of **3B** and **3M** vs **2B** and **2M** are shown in Figure 5. Three peaks **I**, **II** and **III** are observed in both **3B** and **3M**. Both **3B** and **3M** reveal shorter Mn–O and Mn–N bond distances than **2B** and **2M**, in agreement with their DFT-optimized geometrical comparisons (Section S2 in Supporting Information). For instance, **3B** shows a considerably shortened Mn–O bond of 1.60 Å, 2 Mn–N at 2.02 Å, and 3 Mn–N bond distances at 2.22 Å (Table S3, Fit 9), while **3M** reveals a shortened Mn–O at 1.58 Å, 2 Mn–N at 1.95 Å, and 3 Mn–N bond distances at 2.22 Å (Table S3, Fit 17). The bond distances of 1.58–1.60 Å determined for **3B** and **3M** are consistent with the presence of a shortened Mn^IV^=O unit and within 0.05–0.07 Å away from their DFT optimized models (Section S2 in Supporting Information). EXAFS analyses of Mn^IV^=O complexes in a pseudo-octahedral environment have further been reported to vary from 1.58 to 1.70 Å,^13,22–25,34^ thus confirming the presence of a Mn^IV^=Ounit in both **3B** and **3M**. Salen-supported Mn^IV^=O complexes were, for instance, shown to display a bond distance of 1.58 Å by Fujii and co-workers.^34^ Our EXAFS analyses strongly support the Mn^IV^=O formulation for both **3B** and **3M**, consistent with the EPR and ESI-MS data. A Mn=O bond distance of 1.58–1.60 Å in **3B** and **3M** further rules out the formation of a Mn^IV^–OH species, which would theoretically yield a much more elongated Mn–O bond distance of 1.79 Å (Section S2 in Supporting Information).^35^

**Figure 5:**
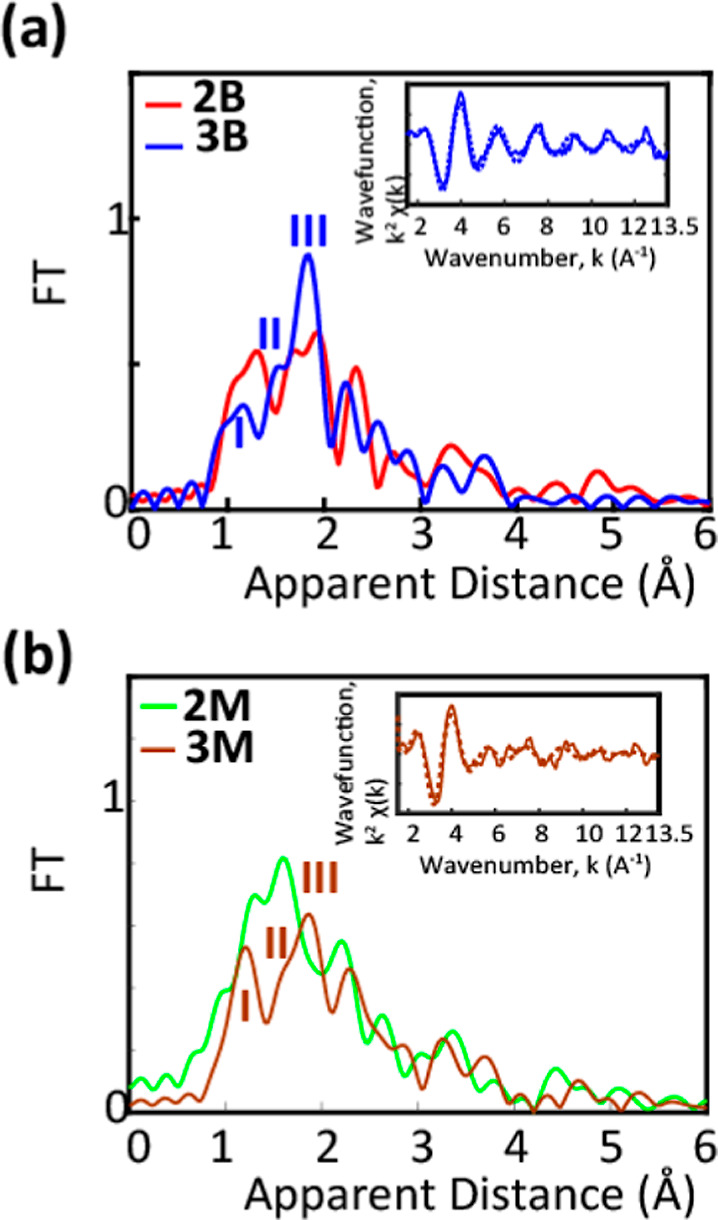
Structural parameters derived via EXAFS: Fourier transforms of k^2^-weighted Mn EXAFS of (a) **2B** (red) and **3B** (blue) and (b) **2M** (green) and **3M** (brown). Inset shown in panels (a) and (b). Top. *K* ^2^[χ­(*k*)] -weighted traces as a function of *k*, the photoelectron wavevector (solid lines) and fitted (dashed lines) of the Mn complexes. Experimental spectra were calculated for *k* values of 1.5–13.5 Å^–1^. Bottom. Structural models of Mn^IV^=O species generated in **3B** and **3M**.

These geometries of **3B** and **3M** were found to be in excellent agreement with the experimental values reported here and in the literature (Figure 6).^15,17–20,36^ For comparison, the corresponding high-valent Fe­(IV)=O complexes were also computed (Figure S19 and Table S7). The Mn=O distance and the Fe=O distances were found to be very similar in all these complexes (Figure 6). Finally, the lowest lying geometries for the benzyl derivatives all exhibited significant intramolecular pi-stacking interactions. The manganese complexes were all found to be on the quartet electronic surface. The methyl substituted ligands favored the intermediate spin surface by more than 4 kcal/mol compared to the benzyl substituted ligands. The computed UV-vis spectra for these compounds are reported in the Supporting Information (Figure S20) and are in agreement with experimental data. A strong absorbance was observed near the 700 nm region for these complexes (Figure S21a). The corresponding transitions responsible for this absorbance are shown in Figure S21. Intrinsic bond orbital analysis of these complexes indicated (Figures 7 and S22) that the oxidation state of the central metal ions is indeed +4. Furthermore, the spin density of the oxygen atom for all these species was also evaluated using the IBO localization scheme. The oxygen atom was found to have a spin density ranging from 0.52 for the [(BnTPEN)­Mn^IV^=O]^2+^ species to 0.50 for [(MeTBEN)­Mn^IV^=O]^2+^ species (Table 1). Both benzyl and methyl substituted ligands were found to have identical spin densities on the oxygen atom, while the TBEN ligand framework had a lower spin density on the manganese atom compared to the TPEN framework due to the electron-donating nature of the TBEN ligands.

**Figure 6:**
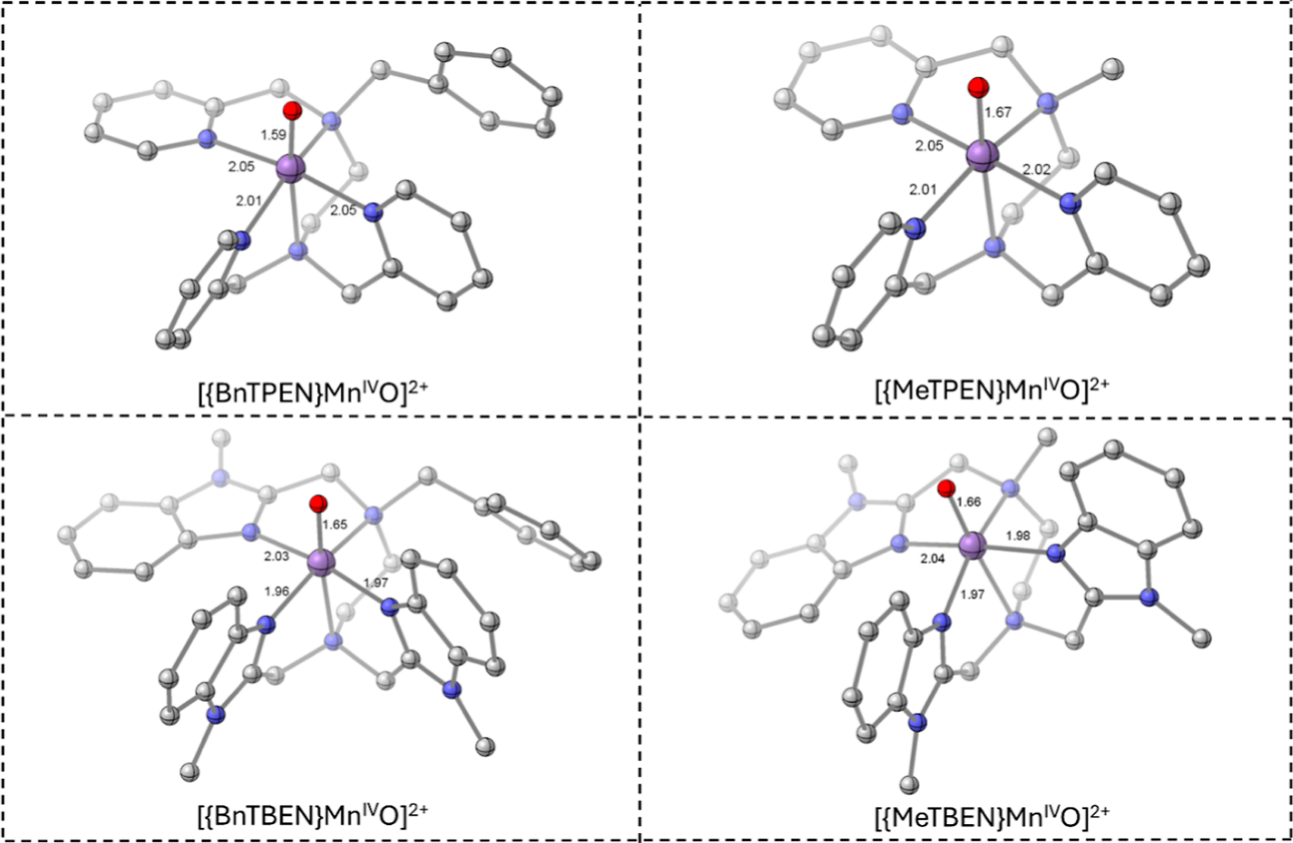
Geometries of various complexes investigated in this study computed at PCM­(CH_3_CN)-M06L/def2tzvpp//PCM­(CH_3_CN)-M06L/def2svp level of theory.^37–39^

**Figure 7:**
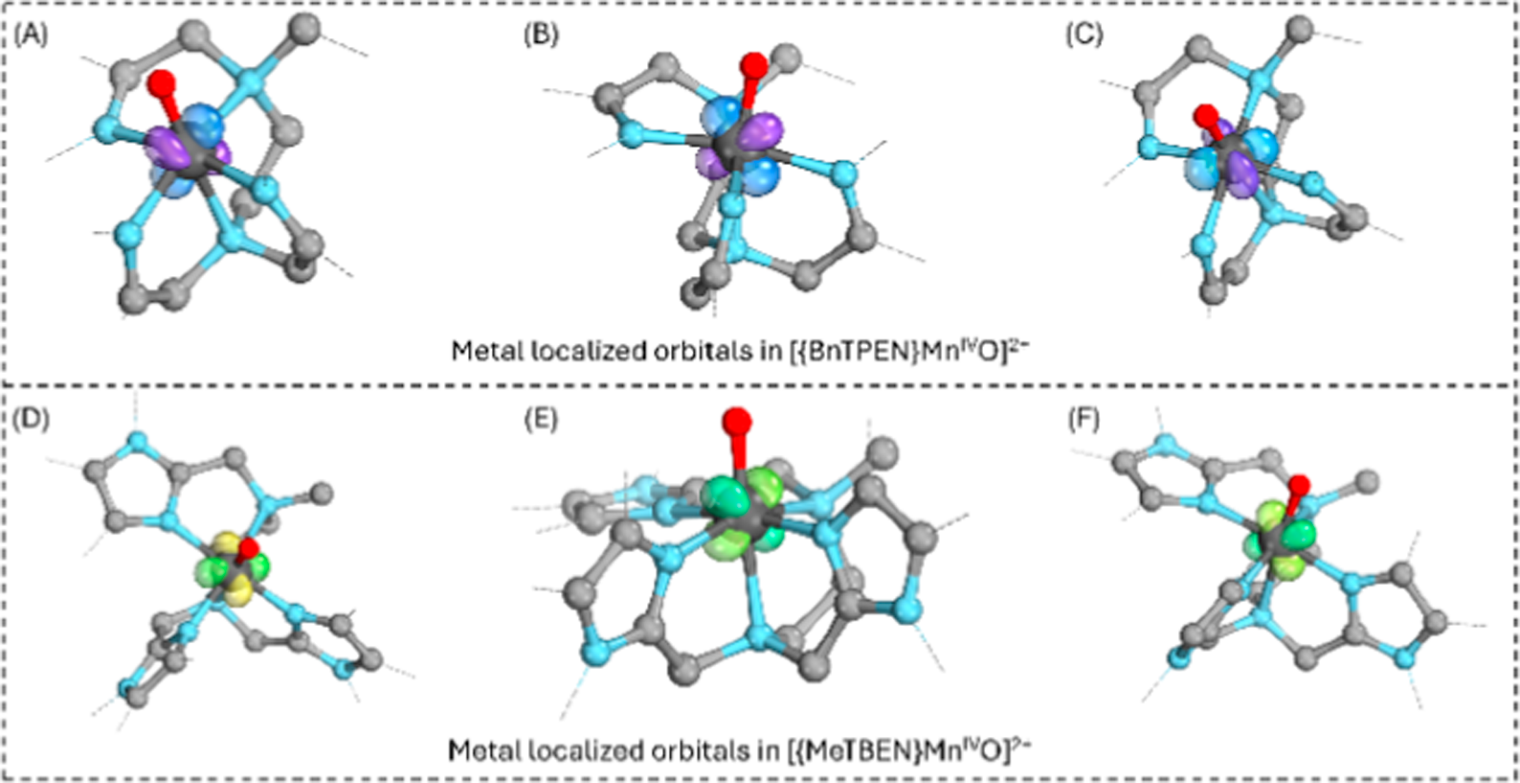
Intrinsic bond orbital analysis of metal localized orbitals for two Mn–oxo complexes with different ligand frameworks. The occupancy numbers for IBOs (A) through (C) are 0.998, 0.994, and 0.988, while the occupancy numbers for IBOs (D) through (F) are 0.988, 0.995, and 0.997, respectively.

**Table 1:** Mulliken Spin Densities on Manganese and Oxygen Atoms Computed at PCM­(CH_3_CN)-M06L/def2tzvpp//PCM­(CH_3_CN)-M06L/def2svp level of theory

Molecule	Mulliken spin on Mn	Mulliken Spin on O
[(BnTPEN)Mn^IV^=O]^2+^	2.51	0.52
[(MeTPEN)Mn^IV^=O]^2+^	2.50	0.51
**3B**	2.45	0.49
**3M**	2.43	0.50

Generation of Mn­(IV)=O by the reaction of Lewis and Brønsted acids with Mn­(III)–O–Ce­(IV), i.e., **2B** and **2M**, parallels previous findings in analogous iron complexes: according to the work reported by Que and co-workers,^36^ the rapid conversion of [(BnTPEN)­Fe^III^–O–Ce^IV^(NO_3_)_4_]^+^ to [(BnTPEN)­Fe^IV^=O]^2+^ upon addition of water demonstrates equilibrium between the two species.^39^ To enhance our existing evaluation, we have carried out similar reactions for [(BnTPEN)­Fe^III^–O–Ce^IV^(NO_3_)_4_]^+^ complex. The reactions with the iron­(III) complex [(BnTPEN)­Fe^III^–O–Ce^IV^(NO_3_)_4_]^+^ revealed that introducing Sc^III^(OTf)_3_ or HClO_4_ produces [(BnTPEN)­Fe^IV^=O]^2+^, and subsequent addition of Ce^III^(NO_3_)_3_ reverts the system back to [(BnTPEN)­Fe^III^–O–Ce^IV^(NO_3_)_4_]^+^ (Figure S23). Importantly, a similar equilibrium is evident in the manganese systems described here: the addition of Ce^III^(NO_3_)_3_ to **3B** or **3M** resulted in a rapid reversion to **2B** and **2M**, respectively, thereby confirming the dynamic equilibrium between species **2B/2M** and **3B/3M** (Scheme 2 and Figure S24).

**Scheme 2:**
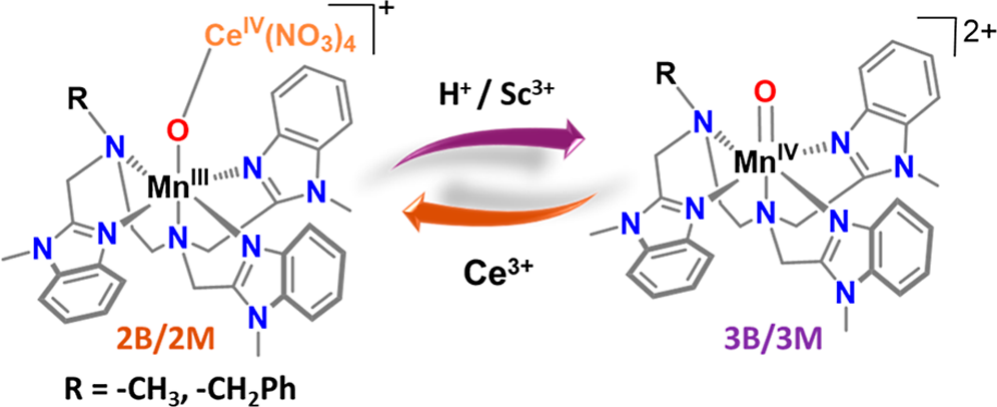
Schematic Diagram Illustrating the Equilibria of Mn^III^–O–Ce^IV^ (**2B/2M**) With High Valent Mn^IV^=O (**3B/3M**) Species in Acetonitrile Solution

### Electron Transfer, OAT, and HAT Reactivity of **2B** and **2M**

Earlier findings established that complex **4** is capable of mediating electron transfer (ET), oxygen atom transfer (OAT), and hydrogen atom transfer (HAT) reactions.^16^ In a similar way, the reactivities of **2B** and **2M** toward ET, OAT, and HAT processes in acetonitrile at room temperature were systematically investigated. The ET behavior was probed using ferrocene-based substrates, including ferrocene, acetylferrocene, and diacetylferrocene. Both **2B** and **2M** reacted with ferrocene and acetylferrocene to yield two equivalents of the corresponding ferrocenium oxidized species, while a reaction with diacetylferrocene afforded one equivalent of the respective oxidized product (Figures S25 and S26).

To examine OAT and HAT activity, thioanisole and 1,4-cyclohexadiene (1,4-CHD) were selected as model substrates, respectively. For HAT, the measured second-order rate constants (*k*_2_) for the reactions of **2B** and **2M** with 1,4-CHD in MeCN at room temperature were 5 × 10^–2^ M^–1^ s^–1^ and 2.4 × 10^–2^ M^–1^ s^–1^, respectively. For OAT, the corresponding *k*_2_ values for thioanisole oxidation were 0.22 M^–1^ s^–1^ (**2B**) and 0.4 M^–1^ s^–1^ (**2M**) in MeCN at room temperature (Figures S27 and S28). Notably, these *k*_2_ values for **2B** and **2M** are markedly higher than those previously reported for complex **4**;^16^ specifically, **2B** is 30 times and **2M** is 55 times more reactive toward thioanisole oxidation. Additionally, the *k*_2_ for 1,4-cyclohexadiene oxidation was found to be five times greater for **2B** and twice as high for **2M** compared to **4** (Table S5). These findings demonstrate that substituting pyridine ligands with benzimidazole ligands significantly enhances both the OAT and HAT reactivity of Mn^III^–O–Ce^IV^ complexes.

### Electron Transfer and OAT Reactivity of 3B and 3M

The electron transfer reactivities of **3B** and **3M** were investigated using ferrocene, acetylferrocene, and diacetylferrocene as reductants. Both **3B** and **3M** reacted with ferrocene and acetylferrocene to produce two equivalents of ferrocenium, whereas diacetylferrocene, which is a weaker reductant, yielded only one equivalent of the oxidized product (Figures S29 and S30). These results indicate that **3B** and **3M** possess oxidation potentials exceeding 0.93 V vs Ag/AgCl, while the (L_5_)­Mn^III^–OH species formed upon one-electron reduction have potentials ranging from 0.64 to 0.93 V vs Ag/AgCl. Previous work by our group showed that [(BnTPEN)­Mn^IV^=O]^2+^ reacts with acetylferrocene and diacetylferrocene to produce only one oxidizing equivalent of ferrocenium, highlighting that the introduction of benzimidazole ligands in **3B** and **3M** leads to enhanced electron transfer activity.^16^

Further, the OAT reactivity of **3B** and **3M** generated by oxidizing **1B** and **1M** with 4 equiv of CAN and 20 equiv of HClO_4_ in acetonitrile was assessed using thioanisole and its para-substituted derivatives in acetonitrile (Table S6). Reactions were conducted with an excess (10–100 equiv) of para-X-thioanisole (X = Me, H, Br, Cl, or CN). Due to the rapid nature of these processes at room temperature, kinetic experiments were instead carried out at −40 °C to obtain reliable rate measurements. A linear relationship between *k*_obs_ and substrate concentration was observed, allowing for the determination of second-order rate constants (*k*_2_). The results for **3B** indicated *k*_2_ values of 100 M^–1^ s^–1^ for *p*-Me-thioanisole, 6 M^–1^ s^–1^ for thioanisole, 2.6 M^–1^ s^–1^ for p-Cl-thioanisole, and 1.25 M^–1^ s^–1^ for *p*-Br-thioanisole at −40 °C. In comparison, **3M** exhibited *k*_2_ values of 70 M^–1^ s^–1^ for thioanisole, 60 M^–1^ s^–1^ for *p*-Cl-thioanisole, 43 M^–1^ s^–1^ for *p*-Br-thioanisole, and 11.5 M^–1^ s^–1^ for *p*-CN-thioanisole under the same conditions (Figures S31 and S32). Product analysis using ESI-MS confirmed the formation of thioanisole oxides, respectively (Figures S33 and S34). Notably, a 2011 report by Nam and co-workers identified [(BnTPEN)­Mn^IV^=O]^2+^ (**5**) as the most reactive Mn^IV^=O for thioanisole oxidation, with a *k*_2_ of 1.3 M^–1^ s^–1^ at 0 °C in TFE/MeCN (19:1).^22^

Jackson and co-workers modified the N4Py ligand framework by substituting two pyridyl groups with quinolinyl moieties, yielding the N2Py2Q scaffold.^9^ This alteration improved the second-order rate constant for the thioanisole oxidation by [(N2Py2Q)­Mn^IV^=O]^2+^ to 9.2 M^–1^ s^–1^ at 25 °C. Remarkably, our current study demonstrates that complexes **3B** and **3M** exhibit substantially higher *k*_2_ values of 6 M^–1^ s^–1^ and 70 M^–1^ s^–1^, respectively, for thioanisole oxidation in acetonitrile at −40 °C, establishing these as the most reactive nonheme Mn^IV^=O species supported by neutral pentadentate ligands reported to date (Figure 8).

**Figure 8:**
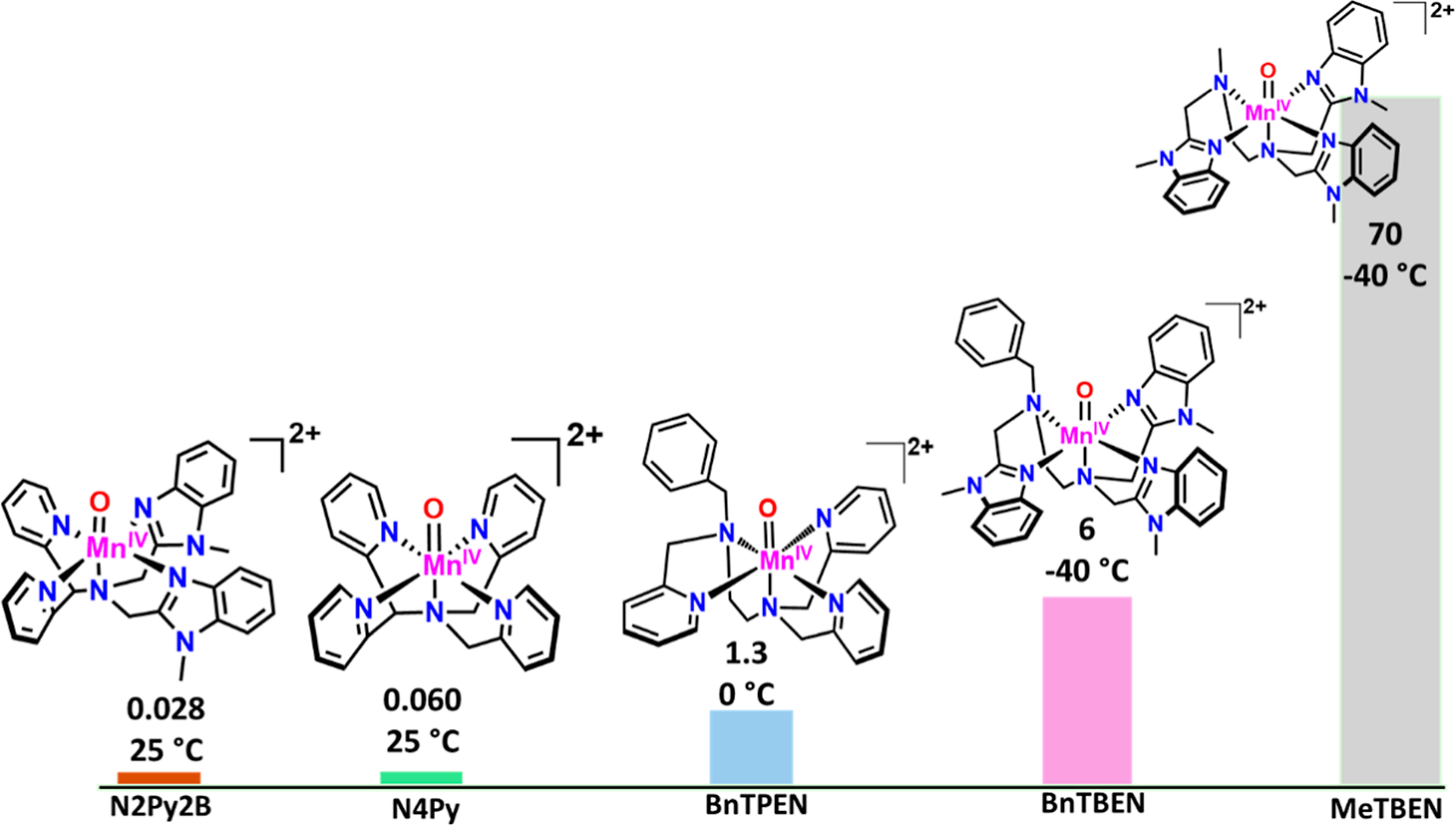
A bar graph illustrates the comparison of thioanisole sulfoxidation rates by Mn^IV^=O species at temperatures mentioned below their respective *k*_2_ values supported by neutral pentadentate ligands.

Analysis of para-substituted thioanisole derivatives revealed that electron-donating substituents result in the highest *k*_2_ values, whereas electron-withdrawing groups correspond to lower *k*_2_ values. A Hammett plot correlating log­(*k*_X_/*k*_H_) with the σ_p_^+^ parameters showed a linear relationship with negative slopes, yielding ρ values of −4.0 for **3B** and −1.2 for **3M** (Figure 9). The negative slopes signify that both **3B** and **3M** exhibit electrophilic character during oxidation. The obtained ρ values align well with those previously reported for electrophilic high-valent Mn^IV/V^=O complexes, underscoring the strong electrophilicity of these newly synthesized complexes.^7^ Although, the addition of 1,4-cyclohexadiene (CHD) and xanthene at 298 K leads to the decay of **3B** and **3M**, the reaction does not follow pseudo-first order behavior.

**Figure 9:**
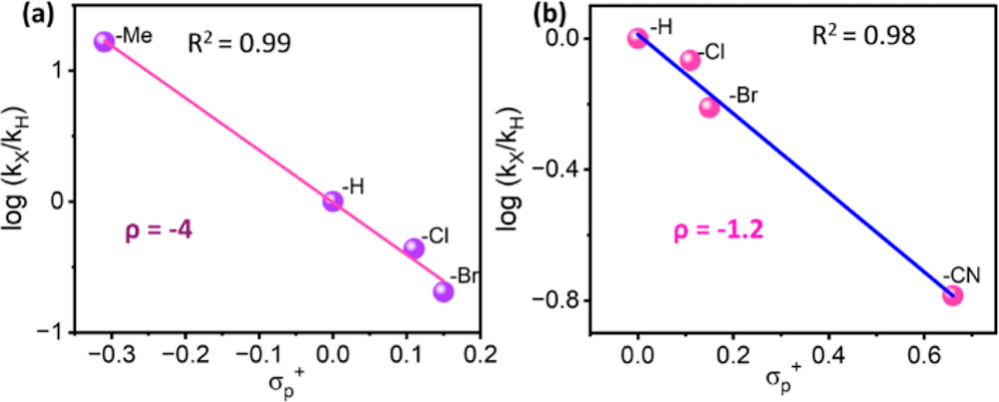
(a) Hammett plots of log­(*k*_X_/*k*_H_) against σ_p_^+^ of para-X-thioanisole derivatives by (a) **3B** and (b) **3M** at −40 °C in MeCN.

Transition states computed for the oxygen atom transfer reactivity toward *p*-substituted thioanisoles indicated that **3B** has higher barriers for OAT toward thioanisoles despite the favorable pi-stacking interaction (Figure 10) present between thioanisole and the phenyl ring of the benzyl substituent, compared to that of **3M**, lending credence to **3M** being an overall stronger oxidizing agent as indicated by the oxidation potentials. A piecewise energy decomposition analysis was performed on these complexes, along with an analysis of the electronic surface along the intrinsic reaction coordinate (IRC). Inspection of the spin densities along the IRC for both complexes reveals a significant change in the spin densities on the oxygen atom, indicating that the oxygen atom behaves more like an oxyl species. Furthermore, oxygen on **3M** behaves more like an oxyl species compared to **3B** based on spin densities. This difference was much more apparent in the hydrogen atom transfer transition states for both complexes, as indicated in the Supporting Information. A similar analysis was carried out for the iron complexes and found that the manganese species have higher oxyl character in all the TS compared to the iron species. Energy decomposition analysis of key transition states for the OAT of thioanisole with complex **3B** revealed higher stabilizing π-stacking interactions due to the extra phenyl ring that the **3M** complex lacks. Finally, despite these favorable interactions, the oxygen atom in **3M** has a higher spin density in the TS, indicating that it is more oxyl-like and therefore provides a rationale for its exceptional reactivity.

**Figure 10:**
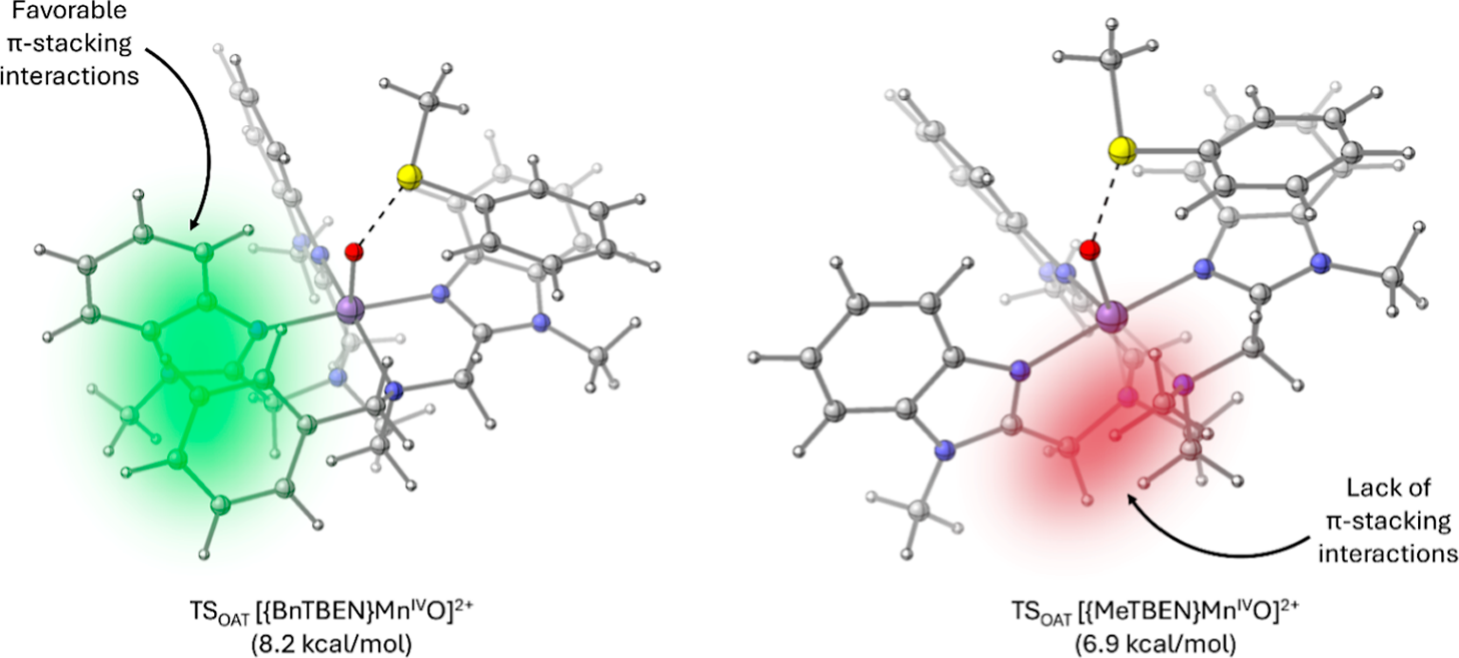
Transition states for the OAT of thioanisole with **3B** and **3M** complexes computed at PCM (CH_3_CN)-M06L/def2tzvpp//PCM (CH_3_CN)-M06L/def2SVP level of theory.

## Conclusion

Understanding the factors that govern the reactivity of high-valent metal–oxo species is essential for advancing the design of efficient oxidation catalysts. This work emphasizes the critical influence of ligand architecture, demonstrated through the synthesis and comprehensive spectroscopic characterization of two novel benzimidazole-supported Mn­(III)–O–Ce­(IV) complexes (**2B** and **2M**). The benzimidazole ligands markedly enhance both oxygen atom transfer and hydrogen atom transfer reactivity compared to pyridine analogs. The in situ generation of highly reactive [(L5)­Mn­(IV)=O]^2+^ intermediates (**3B** and **3M**) upon addition of H^+^ or Lewis acids, combined with spectroscopic and computational analyses, reveals an electrophilic Mn-oxyl-like character that correlates with superior oxidation performance. These findings underscore the pivotal role of benzimidazole coordination in tuning the electronic structure and reactivity of manganese–oxo species, offering valuable insights relevant to both synthetic oxidation catalysis and the biomimetic study of photosystem II.

## Experimental and Computational Section

### Materials and Methods

All chemicals and reagents used in the present study, which were commercially available, were used as received. Ceric ammonium nitrate, ferrocene, acetylferrocene, and diacetylferrocene were acquired from Sigma-Aldrich and Sisco Research Laboratories (SRL). For the spectroscopic studies, HPLC-grade dry CH_3_CN was obtained from Thomas Baker. UV-vis absorption spectroscopic studies were performed using an Agilent 8453 diode-array spectrophotometer. The reaction kinetics were monitored spectrophotometrically using 1 cm quartz cells (λ = 190–1100 nm). The X-band electron paramagnetic resonance (EPR) spectra of **3B** were performed on a Bruker EMXplus with Oxford ESR900 continuous-flow liquid helium cryostat and Oxford ITC503 temperature system at 9 K (9.64 GHz microwave frequency, 9 G modulation amplitude, 20 dB power attenuation). Whereas EPR for **3B** and **3M** were measured on a JES-FA200 ESR spectrometer at 77 K in acetonitrile solution. EPR parameters: [frequency, 9136 MHz; power, 0.995 mW; field center, 490.0 mT, width, ± 500.00Mt; sweep time, 30.0 s; modulation frequency, 100.00 kHz, width, 1 mT; amplitude, 1 mT; and time constant, 0.03 s]. Agilent 6546 LC/Q-TOF was used to record ESI-MS plots under positive ion mode.

### X-ray Crystallography

A single crystal of suitable dimensions was used for data collection. Diffraction intensities were collected on a Bruker APEX-II CCD diffractometer, with graphite-monochromated Mo Kα (0.71073 Å) radiation at 100(2) K. Data were corrected for Lorentz and polarization effects; empirical absorption corrections (SADABS v 2.10) were applied. Using Olex2,^40^ the structures were solved by shelXT^41^ structure solution program using Intrinsic Phasing and, refined with the ShelXL^42^ refinement package using Least Squares minimization. All the non-hydrogen atoms are refined anisotropically, and hydrogen atoms are refined isotropically on their ideal position. CCDC 2367385 and 2369687 contain the supplementary crystallographic data for **1B** and **1M**, respectively. This data can be obtained free of charge from the Cambridge Crystallographic Data Centre via www.ccdc.cam.ac.uk.

### X-ray Absorption Spectroscopy Methods

X-ray absorption spectra were collected at on bending magnet beamline 9–3 at electron energy 6.539 keV and an average current of 100 mA at Stanford Light Source (USA). The radiation was in both cases monochromatized by Si(220) crystal monochromator. The intensity of the X-rays was monitored by three ion chambers (I_0_, I_1_, and I_2_) filled with 70% nitrogen and 30% He and placed before the sample (I_0_) and after the sample (I_1_ and I_2_). Mn metal foil was placed between ion chambers I_1_ and I_2_ and its absorption was recorded with each scan for energy calibration. Mn XAS energy was calibrated by the first maxima in the second derivative of the Manganesés metal foil’s X-ray absorption near edge structure (XANES) spectrum. The samples were kept at 15 K in a He atmosphere at ambient pressure and recorded as fluorescence excitation spectra using a 100-element energy-resolving Ge detector. The solution complexes were measured in the continuous helium flow cryostat in fluorescence mode. Around 10 XAS spectra of each sample were collected. Care was taken to measure at several sample positions on each sample and no more than 5 scans were taken at each sample position. In order to reduce the risk of sample damage by X-ray radiation, 80% flux was used (beam size 2000 μm (Horizontal) × 1000 μm (Vertical)) and no damage was observed scan after scan to any samples. All samples were also protected from the X-ray beam during spectrometer movements by a shutter synchronized with the scan program. Mn XAS energy was calibrated by the first maxima in the second derivative of the Manganese metal X-ray Absorption Near Edge Structure (XANES) spectrum.

### Extended X-ray Absorption Fine Structure Analysis

Athena software^43^ was used for data processing. The energy scale for each scan was normalized using the Manganese metal standard. Data in energy space were pre-edge corrected, normalized, deglitched (if necessary), and background corrected. The processed data were next converted to the photoelectron wave vector (*k*) space and weighted by *k* ^2^. The electron wavenumber is defined as $k={\left[2m\left(E-E_{0}\right)/\hbar^{2}\right]}^{1/2}$ , *E*_0_ is the energy origin or the threshold energy. *K*-space data were truncated near the zero crossings *k* = 1.5 to 13.5 Å^–1^ in Mn EXAFS before Fourier transformation. The *k*-space data were transferred into the Artemis Software for curve fitting. In order to fit the data, the Fourier peaks were isolated separately, grouped together, or the entire (unfiltered) spectrum was used. The individual Fourier peaks were isolated by applying a Hanning window to the first and last 15% of the chosen range, leaving the middle 70% untouched. Curve fitting was performed using ab initio-calculated phases and amplitudes from the FEFF8 ^43^ program from the University of Washington. Ab initio-calculated phases and amplitudes were used in the EXAFS eq eq 1.

1 $$\chi \left(k\right)=\sum_{j}\frac{N_{j}S_{0}^{2}F_{i}\left(k\right)}{kR_{j}^{2}}e^{-2\sigma_{j}^{2}k^{2}}e^{-2R_{j}/\lambda_{j}\left(k\right)}\sin\left(2kR_{j}+\delta_{j}\left(k\right)\right)$$

where *N*_*j*_ is the number of atoms in the *j*th shell; *R*_*j*_ the mean distance between the absorbing atom and the atoms in the *j*th shell; *f*_eff_(π,*k*, *R*_*j*_) is the ab initio amplitude function for shell *j*, and the Debye–Waller term $e^{-2\sigma_{j}^{2}k^{2}}$ accounts for damping due to static and thermal disorder in absorber-backscatterer distances. The mean free path term $e^{-2R_{j}/\lambda_{j}\left(k\right)}$ reflects losses due to inelastic scattering, where λ_*j*_(*k*), is the electron mean free path. The oscillations in the EXAFS spectrum are reflected in the sinusoidal term sin­(2**k** *R*_*j*_ + δ_*j*_(**k**)), where δ_*j*_(**k**)­is the ab initio phase function for shell *j*. This sinusoidal term shows the direct relation between the frequency of the EXAFS oscillations in *k*-space and the absorber-back scatterer distance. *S*_0_^2^ is an amplitude reduction factor.

The EXAFS equation^44^ (eq 1) was used to fit the experimental Fourier isolated data (*q*-space) as well as unfiltered data (*k*-space) and Fourier transformed data (R-space) using *N*, *S*_0_^2^, *E*_0_, *R*, and σ^2^ as variable parameters. *N* refers to the number of coordination atoms surrounding Mn for each shell. The quality of fit was evaluated by *R*-factor (eq 2) and the reduced Chi^2^ value. The deviation in *E*_0_ ought to be less than or equal to 10 eV. *R*-factor less than 2% denotes that the fit is good enough whereas R-factor between 2 and 5% denotes that the fit is correct within a consistently broad model. The reduced Chi^2^ value is used to compare fits as more absorber-backscatter shells are included to fit the data. A smaller reduced Chi^2^ value implies a better fit. Similar results were obtained from fits done in *k*, *q*, and *R*-spaces.

2 $$R‐factor=\frac{\sum_{i}{\left(difference\;between\;data\;\mathrm{and}\;fit_{i}\right)}^{2}}{\sum_{i}{\left(data\right)}^{2}}$$

### DFT Calculations

The DFT optimization calculations were performed using the ORCA (Version 5.0) program package developed by Neese^45^ and co-workers. The geometry optimizations were carried out using the solid-state (XRD) as a starting point. The calculations were carried out using the BP86 exchange–correlation functional^46^ in combination with the triple-ζ valence polarization functions (def2-TZVP),^47^ and the atom-pairwise dispersion correction with the Becke-Johnson damping scheme (D3BJ).^48^ The conductor-like polarizable continuum model (CPCM) was applied to model the acetonitrile solvent.^48^

The RI^49^ approximation was used to accelerate Coulomb and exchange integrals for the ground and excited state calculations, respectively. The default GRID settings were further used for the self-consistent field iterations and for the final energy evaluation. The calculated structures were confirmed to be minima based on a check of the energies and the absence of imaginary frequencies from frequency calculations carried out on the optimized geometries. The frequency run was further combined with a polarizability calculation to calculate the Raman spectral features.

The synthesis of the BnTBEN and MeTBEN ligands was conducted following the procedure reported in our recent article.^50,51^

### Synthesis of **1B**

A 2 mL acetonitrile solution of [Mn^II^(H_2_O)_6_]­(ClO_4_)_2_ (199 mg, 0.55 mmol) was combined with a 2 mL acetonitrile solution of the ligand BnTBEN (320 mg, 0.55 mmol) at room temperature. The reaction mixture was stirred for 3 h, after which half of the solvent was removed under reduced pressure. Upon the addition of diethyl ether, a white precipitate formed. The solid was washed with diethyl ether and dried under reduced pressure. Crystallization was achieved by vapor diffusion of diethyl ether into acetonitrile/methanol (1:1 vol/vol) solution of the compound **1B**. After 1 week, white block-shaped crystals suitable for X-ray analysis were obtained.

### Synthesis of **1M**

A 2 mL acetonitrile solution of [Mn^II^(H_2_O)_6_]­(ClO_4_)_2_ (124.46 mg, 0.34 mmol) was combined with a 2 mL acetonitrile solution of the ligand MeTBEN (174.2 mg, 0.34 mmol) at room temperature. The reaction mixture was stirred for 5 h, after which half of the solvent was removed under reduced pressure. Upon the addition of diethyl ether, a white precipitate formed. The solid was washed with diethyl ether and dried under reduced pressure. Crystallization was achieved by vapor diffusion of diethyl ether into an acetonitrile solution of the compound **1M**. After 2 days, white block-shaped crystals suitable for X-ray analysis were obtained.

## Supplementary Material



## Data Availability

Other data presented here is available in the Supporting Information.

## Notes

Experimental details, synthesis of **1M** and **1B**, characterization of **2M**, **2B**, **3M**, and **3B**, ferrocene reactivity, and other spectroscopic data (PDF)
