# Supplementary material for: Mn(III)–O–Ce(IV) as a Surrogate for Highly Reactive Non-Heme Mn(IV)=O Species Supported by Benzimidazole-Based Ligand
Source: Inorg Chem. 2026 Jun 9;65(24):13730–41. doi: 10.1021/acs.inorgchem.6c02577 (PMC13292347; doi:10.1021/acs.inorgchem.6c02577)
Supplement: Supplementary file 1 [file ic6c02577_si_001.pdf]

Supporting online information for

Mn(III)–O–Ce(IV) as a Surrogate for Highly Reactive Non-heme Mn(IV)=O Species Supported by Benzimidazole-based Ligand

Sikha Gupta,[Ψ,Δ] Parkhi Sharma,[Ψ,Δ] Lucia Velasco,[¥,‖] Divya Lakshmi Hareendran,[Ψ] Markell J. A. Lomax,[Φ] Asterios Charisiadis,[¥] Khyati Jain,[Ψ] Anagha Puthiyadath,[Φ] Timothy A. Jackson,[Φ,] Sharath Chandra Mallojjala,[⊥,*] Dooshaye Moonshiram,[¥,*] Apparao Draksharapu[Ψ,*]*

[Ψ] Southern Laboratories-208A, Department of Chemistry, Indian Institute of Technology Kanpur, Kanpur-208016, India. E-mail: appud@iitk.ac.in

[¥] Instituto de Ciencia de Materiales de Madrid, Consejo Superior de Investigaciones Científicas, Madrid 28049, Spain. E-mail: dooshaye.moonshiram@csic.es

[‖] Departamento de Química Física, Universidad Complutense de Madrid, Avenida Complutense s/n, 28040, Madrid, Spain.

[Φ] The University of Kansas, Department of Chemistry and Center for Environmentally Beneficial Catalysis, Lawrence, Kansas 66045, United States. E-mail: taj@ku.edu

[⊥] Department of Chemistry, Binghamton University, Binghamton, NY 13850, United States. E-mail: sharathc@binghamton.edu

Table S1. Crystal data and structure refinement data of **1B** and **1M**

	**1B**	**1M**
Empirical formula	$C_{38}H_{41}Cl_2MnN_9O_8$	$C_{32}H_{37}Cl_2MnN_9O_8$
Formula weight	877.64	801.54
Temperature/K	100	100
Crystal system	triclinic	monoclinic
Space group	P-1	P21/n
a/Å	11.5281 (4)	20.0523 (9)
b/Å	11.9137 (4)	8.1519 (3)
c/Å	14.3264 (5)	21.9667 (9)
α/°	85.0790 (10)	90
β/°	84.0720 (10)	103.5600 (10)
γ/°	87.0410 (10)	90
Volume/Å^3	1948.07 (12)	3490.7 (2)
Z	2	4
ρ_{calc}g/cm^3	1.496	1.525
μ/mm^{-1}	0.542	0.596
F(000)	910.0	1660.0
Reflections collected	30905	55840
Independent reflections	9667 [R_{int} = 0.0471, R_{sigma} = 0.0481]	8679 [R_{int} = 0.0735, R_{sigma} = 0.0516]
Data/restraints/parameters	9667/0/527	8679/0/474
Goodness-of-fit on F^2	1.038	1.127
Final R indexes [I>=2σ (I)]	R_1 = 0.0499, wR_2 = 0.1205	R_1 = 0.0763, wR_2 = 0.1671
Final R indexes [all data]	R_1 = 0.0632, wR_2 = 0.1286	R_1 = 0.1070, wR_2 = 0.1810

Table S2. Selected bond lengths (Å) of [(BnTBEN)Mn^{II}($OClO_3$)](ClO_4) (**1B**) and [(MeTBEN)Mn^{II}(CH_3CN)](ClO_4)$_2$ (**1M**).

	[(BnTBEN)Mn^{II}($OClO_3$)](ClO_4) **(1B)**	[(MeTBEN)Mn^{II}(CH_3CN)](ClO_4)$_2$ (**1M**).
	**Bond length (Å)**	**Bond length (Å)**
Mn-N1	2.240(4)	2.216(15)
Mn-N2	2.180(4)	2.270(10)
Mn-N3	2.437(4)	2.147(16)
Mn-N4	2.203 (4)	2.465(16)
Mn-N5	2.385(4)	2.180(16)
Mn-O1	2.235(5)	-
Mn-N6	-	2.339(5)

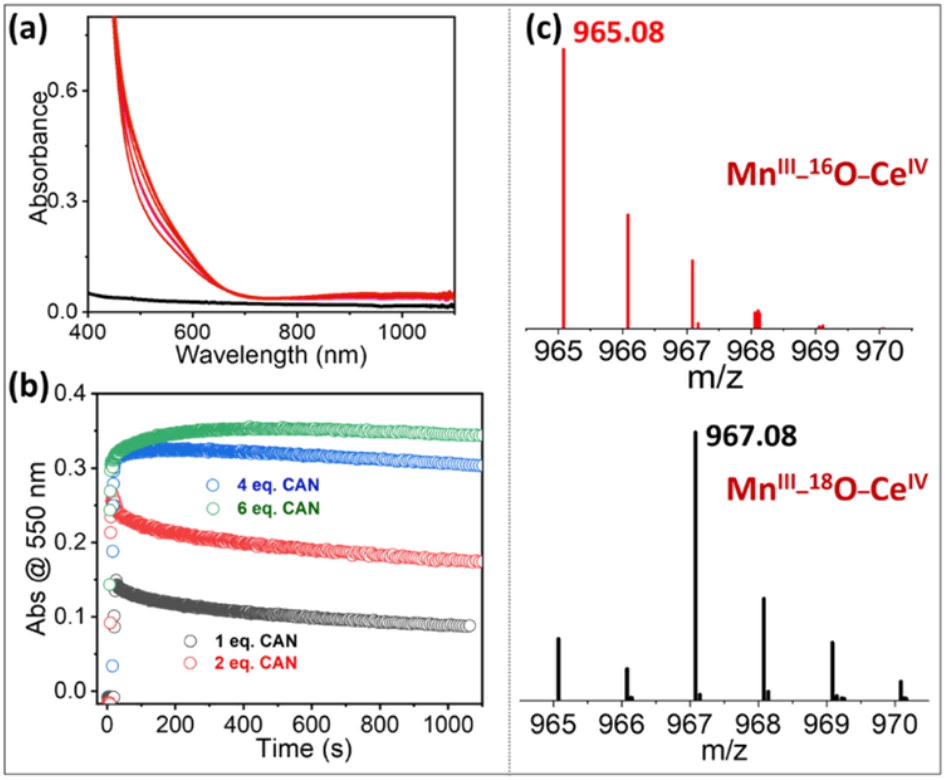

Figure S1. (a) UV-Vis spectral changes showing the formation of **2M** after adding 20 μL H_2O and 4 equiv of CAN to 1 mM **1M** in MeCN at 25 °C. (b) Absorbance vs time plot illustrating the formation of 550 nm species with different equivalents (1-6 eq.) of CAN in MeCN at 25 °C. (c) ESI-MS analysis for species **2M** (red) and **^{18}O-2M** (black). *Condition to generate **2M** and **^{18}O-2M***: **1M** + 20 μL $H_2O/H_2{}^{18}O$ + 4 equiv of CAN at 25 °C in MeCN.

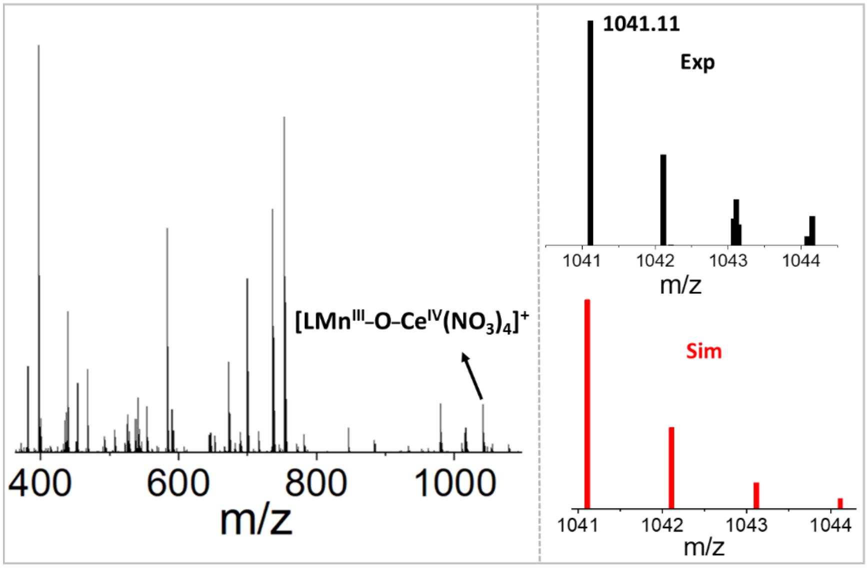

Figure S2. ESI-MS analysis of **2B** in MeCN. *Condition to generate **2B***: 1 mM **1B** in MeCN + 20 μL H_2O + 4 equiv of CAN at 25 °C.

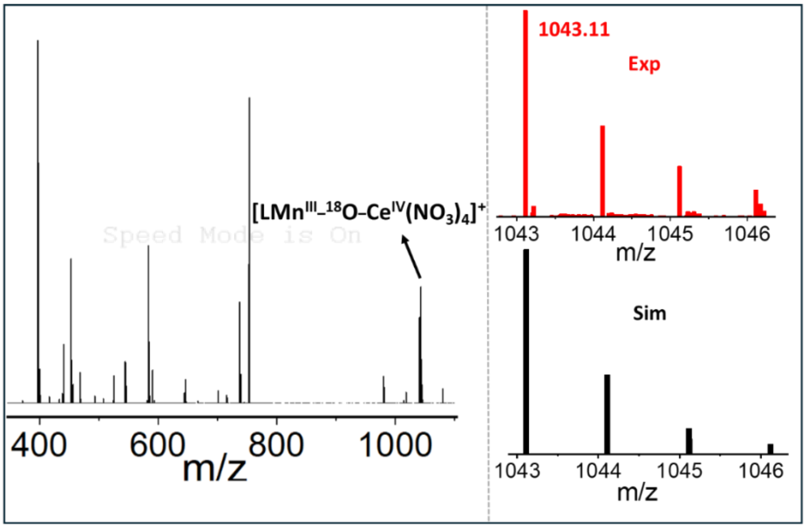

Figure S3. ESI-MS analysis of **^{18}O-2B** in MeCN. *Condition to generate **2B***: 1 mM **1B** in MeCN + 20 μL $H_2{}^{18}O$ + 4 equiv of CAN at 25 °C.

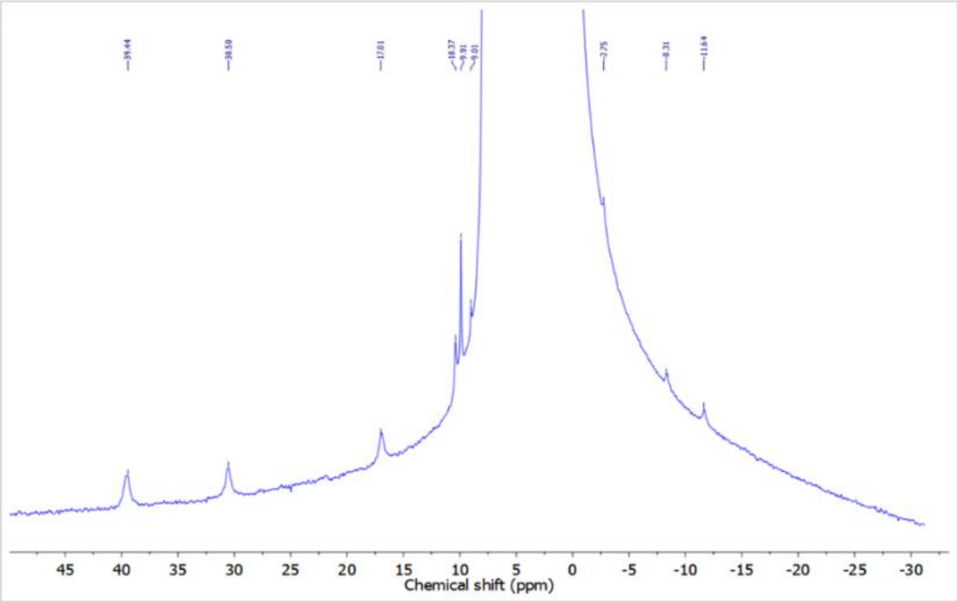

Figure S4. ^{1}H NMR (400 MHz) spectrum of **2B** in CD_3CN at room temperature. *Conditions to prepare **2B**:* 5.5 mM **1B** + 20 μL D_2O + 4 equiv of CAN at 25 °C in CD_3CN.

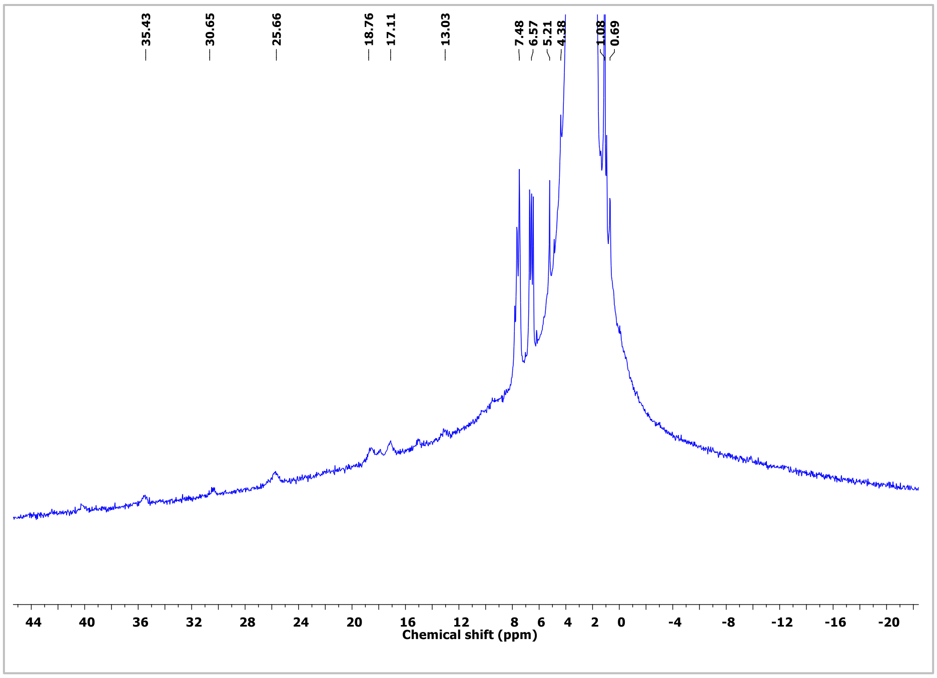

Figure S5. ^{1}H NMR (400 MHz) spectrum of **2M** in CD_3CN at room temperature. *Conditions to prepare **2M**:* 5 mM **1M** + 20 μL D_2O + 4 equiv of CAN at 25 °C in CD_3CN.

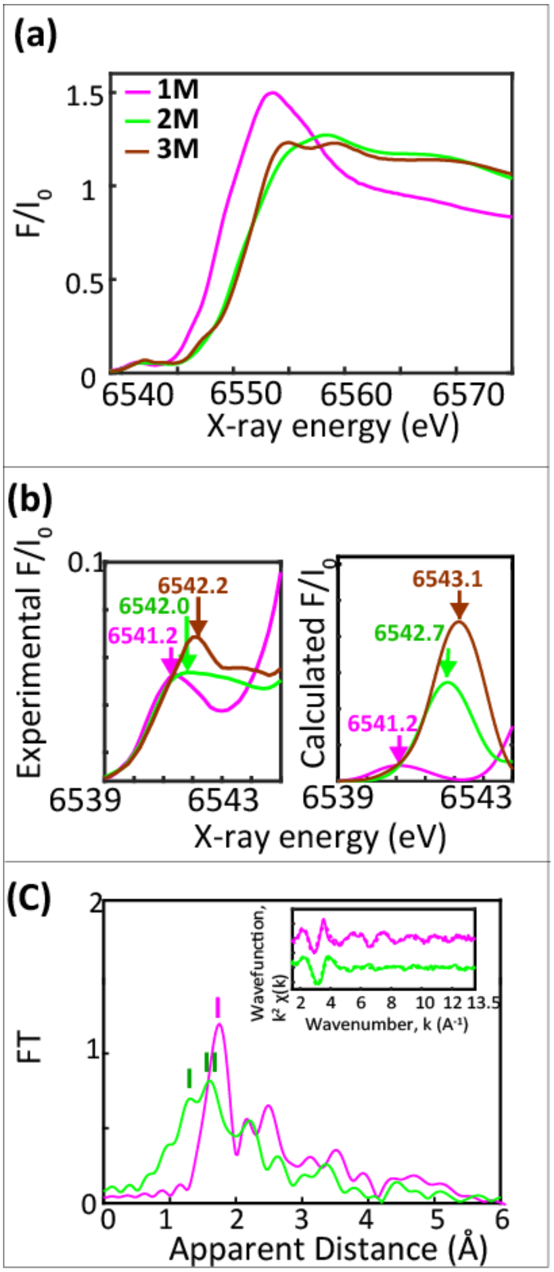

Figure S6. Structural parameters derived via XANES and EXAFS: Normalized Mn K-edge XANES spectra recorded at 15 K of (a) **1M**, **2M**, and **3M** (shown in magenta, green, and brown, respectively). (b) (left) Zoom-in of the pre-edge region of **1M**, **2M**, and **3M**. (right) TD-DFT XANES calculated pre-edges of three models generated from **1M**, namely a Mn^{II} complex coordinated to an ACN molecule (magenta), a Mn^{IV}-O-Ce^{IV} intermediate (green) and a Mn^{IV}=oxo intermediate (brown). Fourier

transforms of k²-weighted Mn EXAFS of (**c**) **1M** and **2M** (in magenta and green respectively). Inset shown in panel-$K^2[\chi(k)]$ -weighted traces as a function of *k*, the photoelectron wavevector (solid lines) and fitted (dashed lines) of the Mn complexes. Experimental spectra were calculated for *k* values of 1.5–13.5 Å^{-1}.

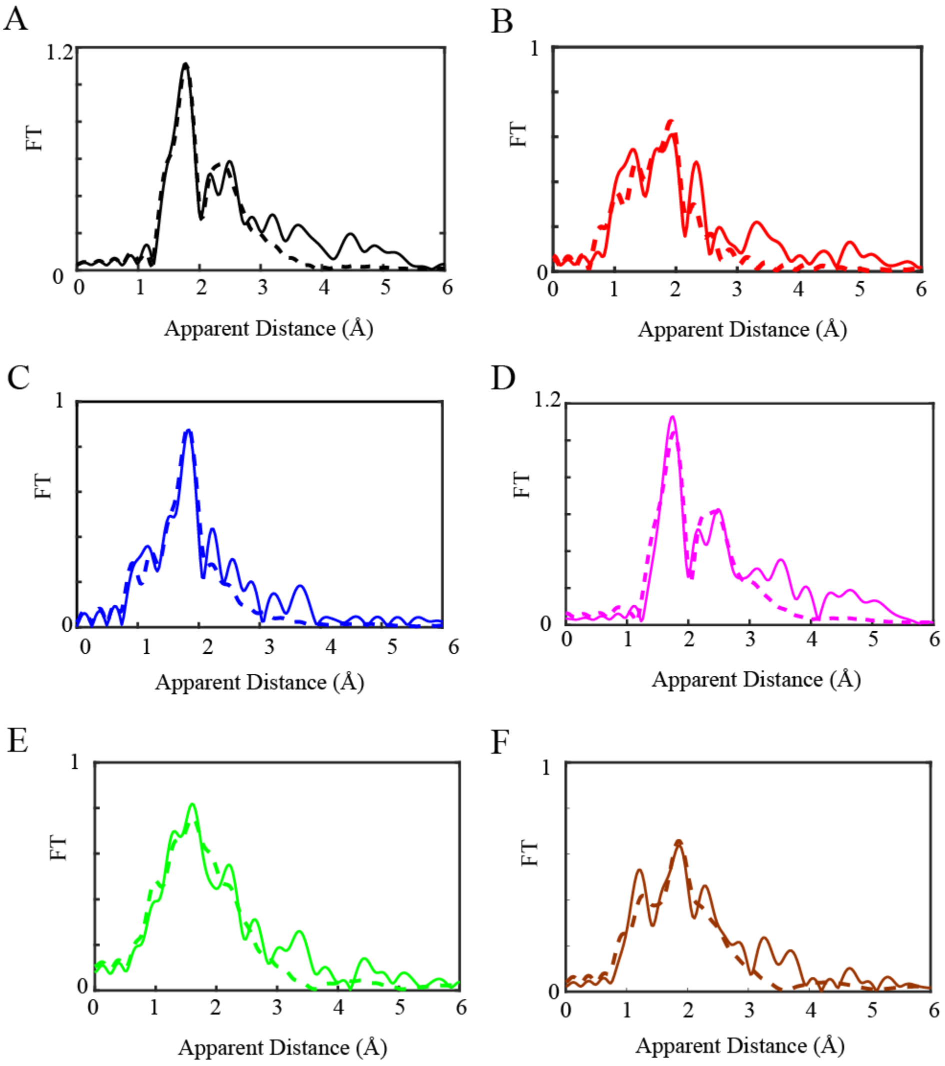

Figure S7. Fourier transforms of k²-weighted Mn of **1B** (black), **2B** (red), **3B** (blue), **1M** (magenta), **2M** (green), and **3M** (brown) in solid lines together with their respective fits (Fits 3, 6, 9, 12, 14, and 17 in Table 3) shown in dashed lines.

Table S3. EXAFS Fit parameters

Sample	Fit	Region	Shell,N	R, Å	E_0	ss.^{2} (10^{-3})	R-factor	Reduced Chi Squared
**1B**	1	I	Mn-N,6	2.21	2.4	1.0	0.0112	129
	2	I	Mn-N,4<br>Mn-N,2	2.23<br>2.44	7.4	0.3<br>0.4	0.0299	109
	**3**	**I,II**	**Mn-N,4<br>Mn-N,2<br>Mn-C,9**	**2.23<br>2.54<br>3.10**	**6.3**	**0.5<br>1.9<br>2.3**	**0.0381**	**96**
**2B**	4	I	Mn-O, 1<br>Mn-N,5	1.70<br>2.26	15	5.9<br>2.7	0.0737	255
	5	I	Mn-O,1<br>Mn-N,2<br>Mn-N,3	1.70<br>1.99<br>2.22	8.2	6.8<br>1.0<br>0.7	0.0478	422
	**6**	**I,II**	**Mn-O,1<br>Mn-N,2<br>Mn-N,3<br>Mn-C,9**	**1.70<br>2.07<br>2.26<br>2.84**	**7.6**	**6.8<br>2.0<br>0.5<br>4.1**	**0.0860**	**215**
**3B**	7	I	Mn-O,1<br>Mn-N,5	1.60<br>2.20	10	4.5<br>2.2	0.1152	846
	8	I	Mn-O,1<br>Mn-N,2<br>Mn-N,3	1.61<br>1.99<br>2.21	7.6	4.4<br>1.5<br>0.4	0.0215	394
	**9**	**I,II**	**Mn-O,1<br>Mn-N,2<br>Mn-N,3<br>Mn-C,9**	**1.60<br>2.02<br>2.22<br>2.82**	**7.4**	**4.6<br>2.5<br>4.9<br>3.8**	**0.0428**	**216**
**1M**	10	I	Mn-N,6	2.20	3.6	1.1	0.0365	3766
	11	I	Mn-N,4<br>Mn-N,2	2.23<br>2.44	8.6	0.3<br>0.2	0.0578	171
	**12**	**I,II**	**Mn-N,4<br>Mn-N,2<br>Mn-C,9**	**2.23<br>2.54<br>3.11**	**6.3**	**0.5<br>1.9<br>2.2**	**0.0330**	**70**

**2M**	13	I	Mn-O, 1<br>Mn-N,5	1.69<br>2.14	10	4.2<br>2.1	0.0113	135
	**14**	**I,II**	**Mn-O,1**<br>**Mn-N,5**<br>**Mn-C,9**	**1.70**<br>**2.13**<br>**2.94**	**8.8**	**4.7**<br>**2.6**<br>**3.0**	**0.0240**	**119**
**3M**	15	I	Mn-O,1<br>Mn-N,5	1.58<br>2.31	19	3.5<br>3.4	0.07	400
	16	I	Mn-O,1<br>Mn-N,2<br>Mn-N,3	1.58<br>1.98<br>2.24	12	2.6<br>1.5<br>0.8	0.03	610
	**17**	**I,II**	**Mn-O,1**<br>**Mn-N,2**<br>**Mn-N,3**<br>**Mn-C9**	**1.58**<br>**1.95**<br>**2.22**<br>**2.90**	**9.0**	**2.4**<br>**1.4**<br>**0.9**<br>**3.6**	**0.05**	**286**

***** Region **I** corresponds to the fit between 1- 2.1/2.5 Å, in apparent distance scale and Region **I,II** to the fit between 1- 2.8/3.0 Å. We note that the data resolution, the ability to distinguish between 2 bond distances, given by $\pi/2\Delta k$ is ~ 0.130 Å.

Table S4. Selected theoretical bond lengths (Å) for the complexes **1B**, **1M**, and all their derivatives.

Compounds	Mn-N1	Mn-N2	Mn-N3	Mn-N4	Mn-N5	Mn-O1	Mn-N6
$[(BnTBEN)Mn^{II}(CH_3CN)]^{2+}$ **(1B)**	2.44	2.18	2.18	2.16	2.45	-	2.19
$[(BnTBEN)(Mn^{III}OCe^{IV})(NO_3)_4]^{+}$	2.44	2.08	2.21	2.02	2.27	1.75	-
$[(BnTBEN)(Mn^{III}OCe^{IV})(OH_2)(NO_3)_4]^{+}$	2.41	2.09	2.23	2.02	2.28	1.74	-
$[(BnTBEN)(Mn^{IV}OCe^{IV})(NO_3)_4]^{2+}$ **(2B)**	2.27	2.05	1.97	2.00	2.34	1.70	-
$[(BnTBEN)(Mn^{IV}OCe^{IV})(OH_2)(NO_3)_4]^{2+}$	2.27	2.05	1.98	1.99	2.34	1.70	-
$[(BnTBEN)Mn^{IV}(O)]^{2+}$ **(3B)**	2.24	2.08	1.95	1.99	2.33	1.65	-
$[(BnTBEN)Mn^{IV}(OH)]^{3+}$	2.28	2.02	1.96	1.99	2.30	1.79	
$[(MeTBEN)Mn^{II}(CH_3CN)]^{2+}$ (**1M**)	2.47	2.18	2.17	2.16	2.46	-	2.19

[(MeTBEN)($Mn^{III}OCe^{IV}$) $(NO_3)_4$]$^{+}$	2.46	2.08	2.21	2.02	2.27	1.74	-
[(MeTBEN)($Mn^{III}OCe^{IV}$) (OH_2) $(NO_3)_4$]$^{+}$	2.43	2.09	2.23	2.02	2.28	1.74	-
[(MeTBEN)($Mn^{IV}OCe^{IV}$) $(NO_3)_4$]$^{2+}$ **(2M)**	2.28	2.04	1.95	1.99	2.33	1.70	-
[(MeTBEN)($Mn^{IV}OCe^{IV}$) (OH_2) $(NO_3)_4$]$^{2+}$	2.28	2.05	1.97	1.99	2.33	1.69	-
[(MeTBEN)Mn^{IV}(O)]$^{2+}$ **(3M)**	2.25	2.09	1.94	1.98	2.32	1.65	-
[(MeTBEN)Mn^{IV}(OH)]$^{3+}$	2.13	2.01	1.94	1.97	2.16	1.79	-

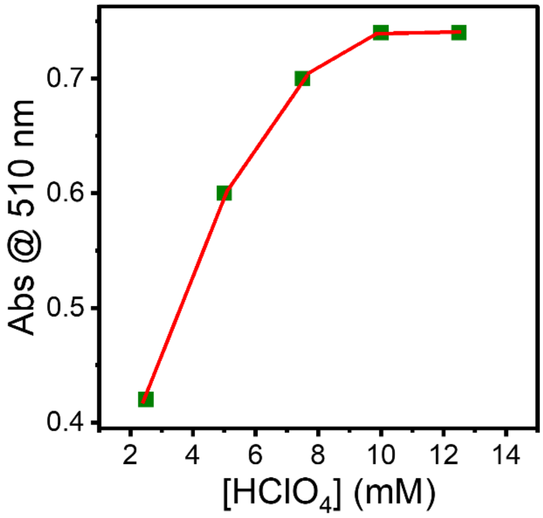

Figure S8. Absorbance vs [$HClO_4$] plot illustrating the formation of **3B** obtained by the addition of different equivalents (5-20 equiv) of $HClO_4$ to **2B** in MeCN at room temperature. *Condition to generate **2B***: 0.5 mM **1B** + 20 µL H_2O + 4 equiv of CAN at 25 ^{o}C in MeCN.

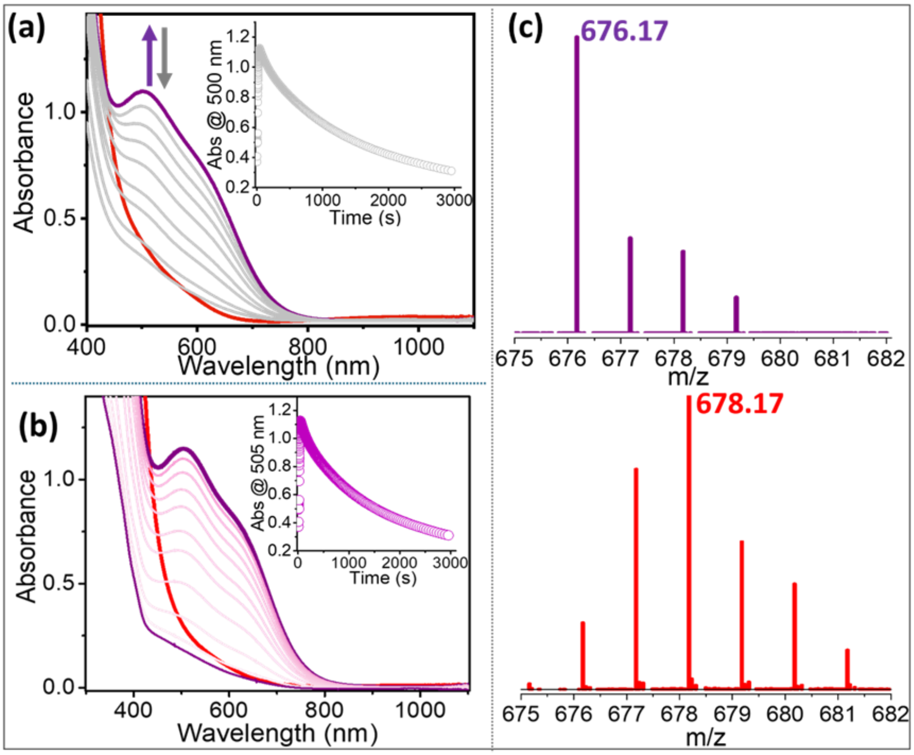

Figure S9. (a) UV-Vis spectral changes showing the formation of **3M** after adding 20 equiv of $HClO_4$ to **2M** in MeCN at 25 °C. (b) UV-Vis spectra showing the generation of **3M** upon adding 10 equiv of $Sc^{III}(OTf)_3$ (c) ESI-MS analysis for species **3M** (purple) and **^{18}O-3M** (red). Note: Partial incorporation of ^{18}O into species **3M** is noticed. *Condition to generate **2M** and* **^{18}O-2M**: **1M** + 20 µL $H_2O/H_2{}^{18}O$ + 4 equiv of CAN at 25 °C in MeCN. *Condition to generate **3M/^{18}O-3M**: **2M/^{18}O-2M** +* 20 equiv of $HClO_4$ in MeCN at 25 °C.

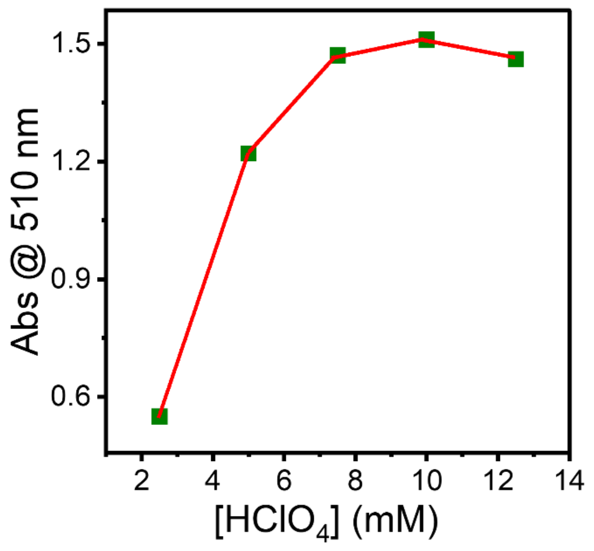

Figure S10. Absorbance vs [$HClO_4$] plot illustrating the formation of **3M** obtained by the addition of different equivalents (5-20 equiv) of $HClO_4$ to **2M** in MeCN at 25 °C. *Condition to generate **2M***: 0.5 mM **1M** + 20 µL H_2O + 4 equiv of CAN at 25 °C in MeCN.

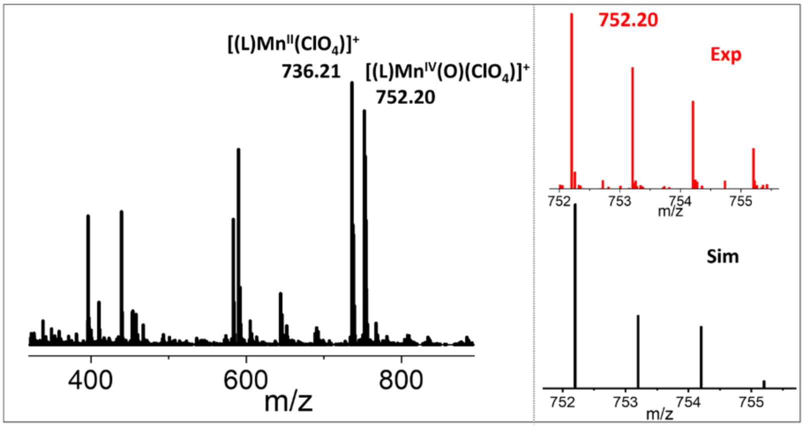

Figure S11. ESI-MS analysis of **3B** in MeCN. *Condition to generate **3B***: adding 20 equiv of $HClO_4$ to **2B** in MeCN at 25 °C. *Condition to generate **2B***: 1mM **1B** in MeCN + 20 µL H_2O + 4 equiv of CAN at 25 °C.

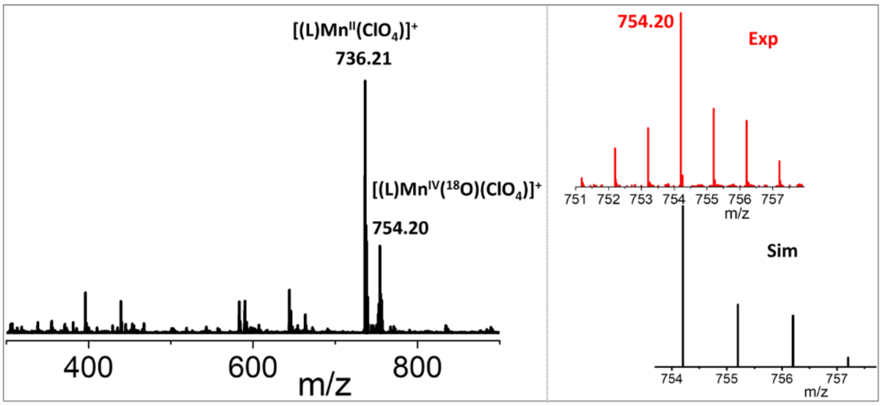

Figure S12. ESI-MS analysis of ^{18}**O-3B** in MeCN. *Condition to generate* ^{18}**O-3B**: adding 20 equiv of $HClO_4$ to ^{18}**O-2B** in MeCN at 25 °C. *Condition to generate* ^{18}**O-2B:** 1 mM **1B** in MeCN + 20 μL $H_2{}^{18}O$ + 4 equiv of CAN at 25 °C.

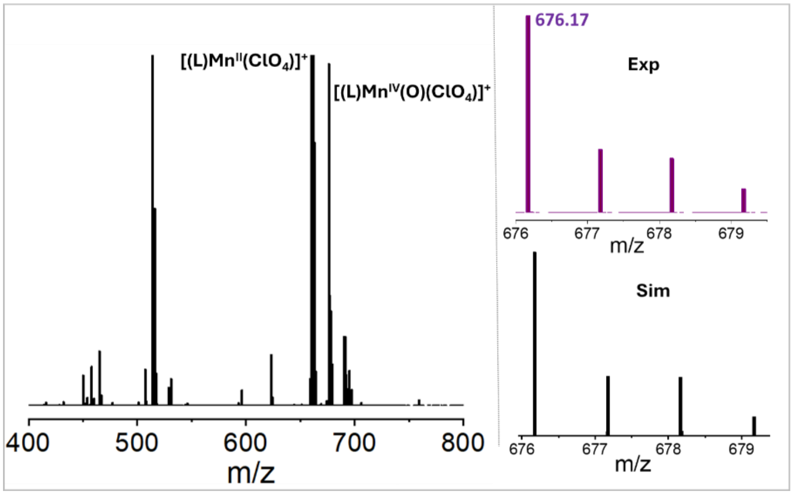

Figure S13. ESI-MS analysis of **3M** in MeCN. *Condition to generate **3M***: adding 20 equiv of $HClO_4$ to **2M** in MeCN at 25 °C. *Condition to generate **2M***: 1 mM **1M** in MeCN + 20 μL H_2O + 4 equiv of CAN at 25 °C.

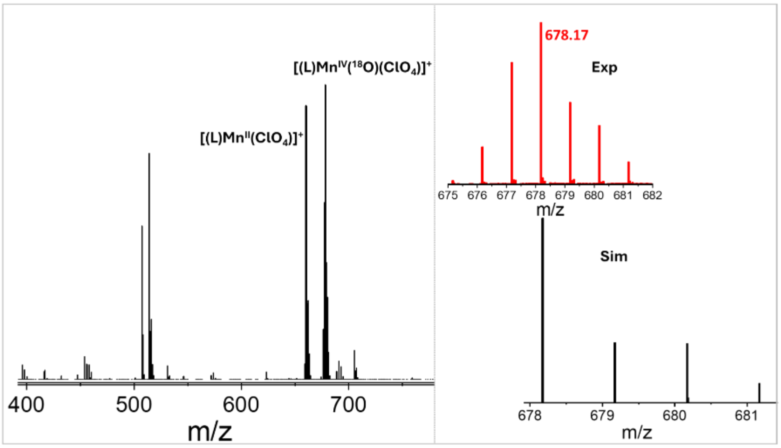

Figure S14. ESI-MS analysis of **^{18}O-3M** in MeCN. *Condition to generate* **^{18}O-3M**: adding 20 equiv of $HClO_4$ to **^{18}O-2M** in MeCN at 25 °C. *Condition to generate* **^{18}O-2M:** 1 mM **1M** in MeCN + 20 µL $H_2{}^{18}O$ + 4 equiv of CAN at 25 °C.

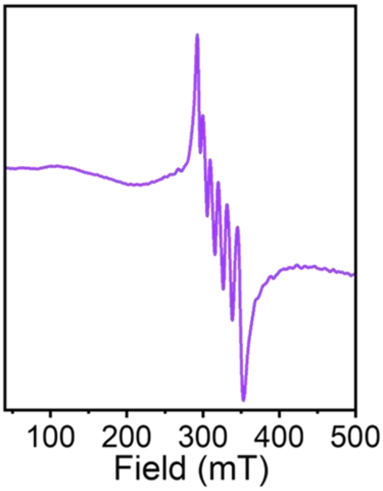

Figure S15. Perpendicular mode EPR spectrum of **3B** at 77 K in MeCN. *Condition to generate* ***3B:*** *2 mM* ***2B*** *in MeCN* + 20 equiv $HClO_4$ in MeCN at room temperature**.** *Condition to generate* ***2B***: 2 mM **1** in MeCN + 20 µL H_2O + 4 equiv of CAN at 25 °C.

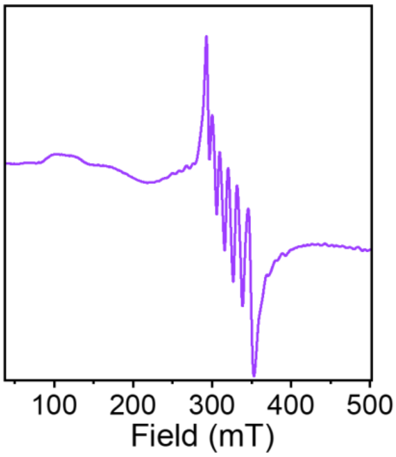

Figure S16. Perpendicular mode EPR spectrum of **3M** at 77 K in MeCN. *Condition to generate **3M**: 2 mM **2M** in MeCN +* 20 equiv $HClO_4$ in MeCN at room temperature**.** *Condition to generate **2M***: 2 mM **1M** in MeCN + 20 µL H_2O + 4 equiv of CAN at 25 °C.

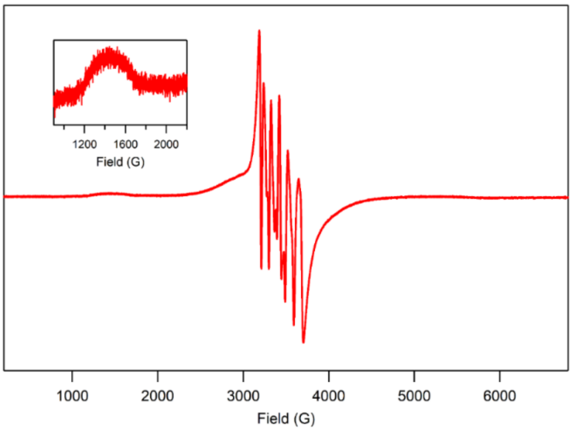

Figure S17. Perpendicular-mode, X-band EPR spectrum of **3B** at 9 K in MeCN. Experimental conditions: *5 mM **2B** in MeCN +* 20 equiv $HClO_4$ in MeCN at 25 °C**.** *Condition to generate **2B***: 5 mM **1** in MeCN + 20 µL H_2O + 4 equiv of CAN at 25 °C.

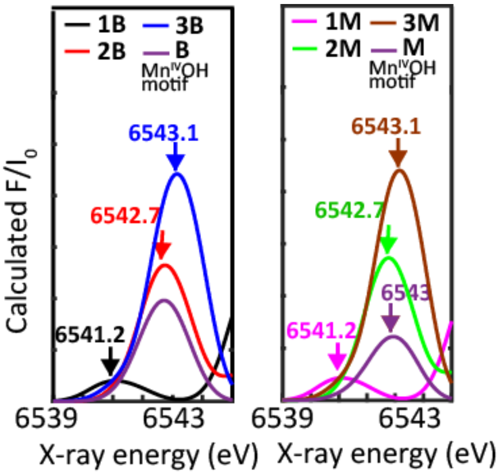

Figure S18. (left) TD-DFT XANES calculated pre-edges of four models generated from **1B** and (right) **1M**, namely a Mn^{II} complex coordinated to an MeCN molecule, a Mn^{IV} oxidized species with a loosely coordinated Ce^{IV} intermediate, a Mn^{IV}=O intermediate, and a Mn^{IV}-OH intermediate.

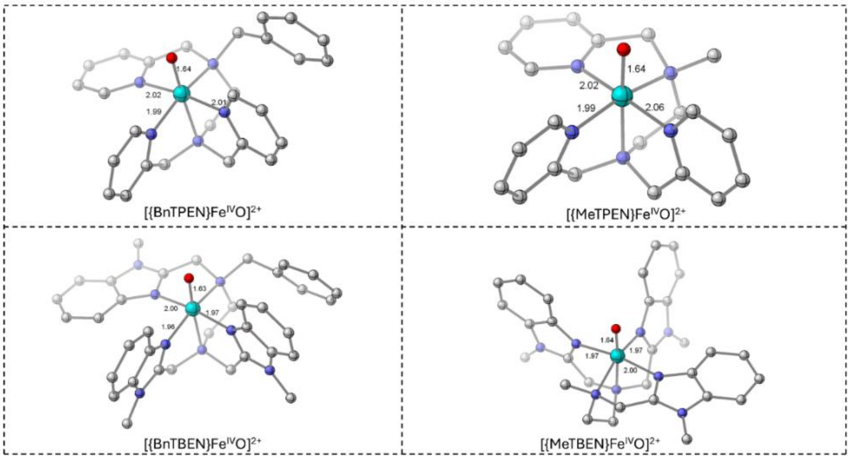

Figure S19. The geometries of various complexes investigated in this study computed at PCM(CH3CN)-M06L/def2tzvpp/PCM(CH3CN)-M06L/def2svp level of theory.

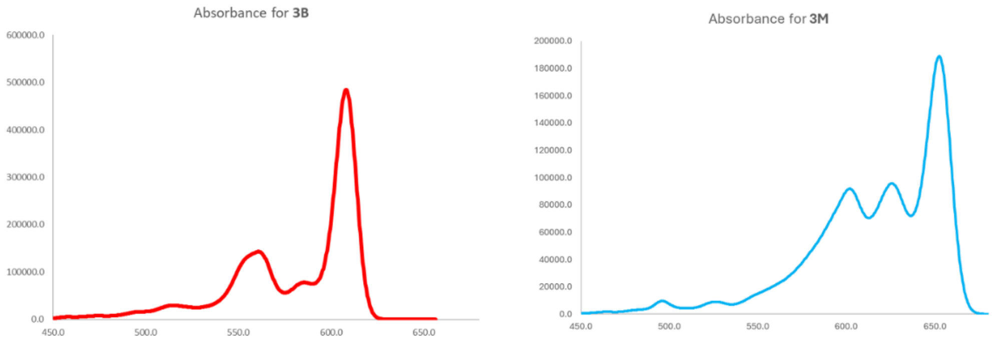

Figure S20. The computed UV/Vis spectra for **3B** and **3M** computed at CPCM(CH3CN)-M06L/def2tzvpp/PCM(CH3CN)-M06L/def2svp level of theory.

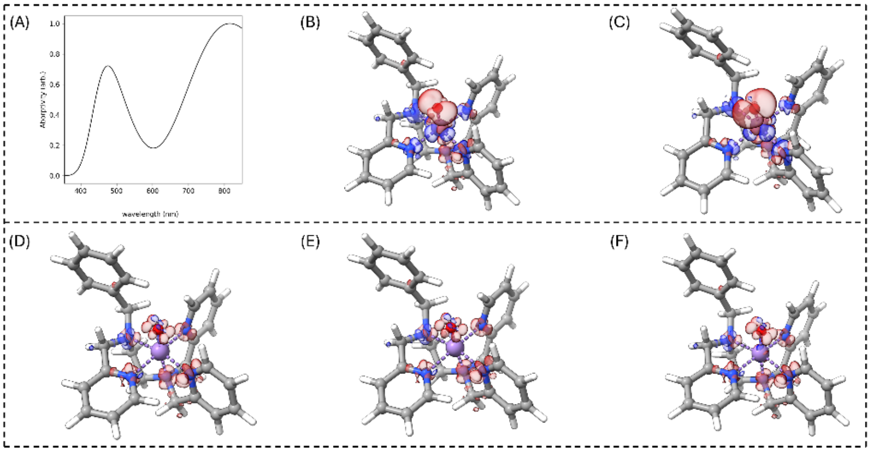

Figure S21. (a) The UV/Vis spectrum and electron density difference maps generated for the transitions observed at 490 nm and 800 nm for **3B** as computed at UM06L/ZORA-def2tzvp//UM06L/def2tzvp level of theory in acetonitrile using CPCM as the solvent model, (b-c) correspond to the transitions observed around 800 nm and (d-f) correspond to the transitions observed around 470 to 530 nm. Note that the key spectral features remain intact even at a different level of theory, as demonstrated in Figure S20.

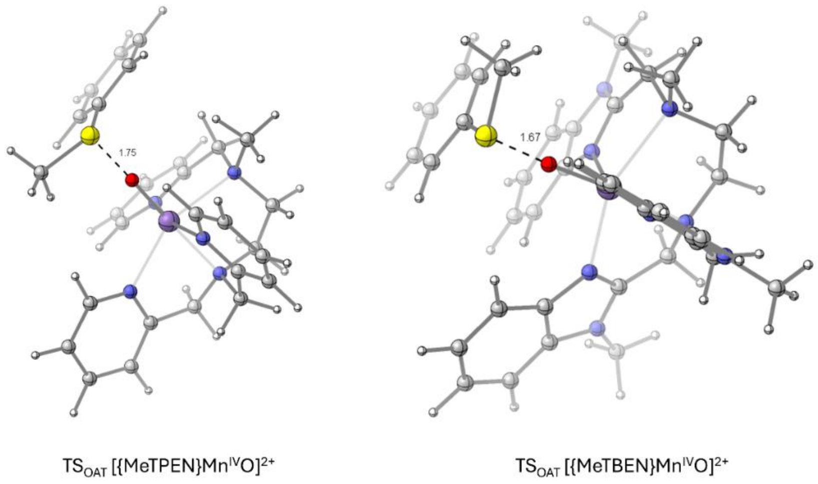

Figure S22. TS computed at M06L/6-31+G(d) in acetonitrile with PCM solvation model for both the ligand frameworks.

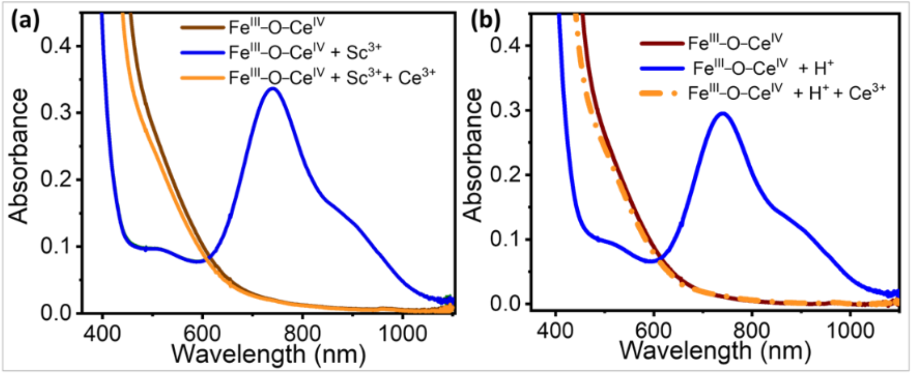

Figure S23. (a) UV-Vis spectra showing the generation of [(BnTPEN)Fe^{III}–O–Ce^{IV}(NO$_3$)$_4$]$^+$ by the reaction of 1 mM (BnTPEN)Fe]$^{2+}$ in MeCN with 2 equiv of CAN and 20 µL H_2O (brown), followed by the generation of [(BnTPEN)Fe^{IV}=O]$^{2+}$ with 5 equiv Sc(OTf)$_3$ to [(BnTPEN)Fe^{III}–O–Ce^{IV}(NO$_3$)$_4$]$^+$ (blue). [(BnTPEN)Fe^{IV}=O]$^{2+}$ goes back to [(BnTPEN)Fe^{III}–O–Ce^{IV}(NO$_3$)$_4$]$^+$ after adding 10 equiv of Ce(III)(NO$_3$)$_3$ (orange). (b) UV-Vis spectra showing the generation of [(BnTPEN)Fe^{III}–O–Ce^{IV}(NO$_3$)$_4$]$^+$ by the reaction of 1 mM (BnTPEN)Fe]$^{2+}$ in MeCN with 2 equiv of CAN and 20 µL H_2O (brown), followed by the generation of [(BnTPEN)Fe^{IV}=O]$^{2+}$ with 5 equiv of $HClO_4$ to [(BnTPEN)Fe^{III}–O–Ce^{IV}(NO$_3$)$_4$]$^+$ (blue). [(BnTPEN)Fe^{IV}=O]$^{2+}$ goes back to [(BnTPEN)Fe^{III}–O–Ce^{IV}(NO$_3$)$_4$]$^+$ after adding 10 equiv of Ce(III)(NO$_3$)$_3$ (orange).

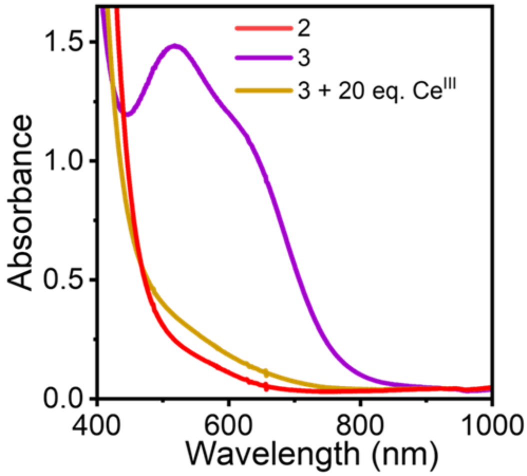

Figure S24. UV-Vis absorption spectra depicting the generation of **2** by the reaction of 0.5 mM **1** in MeCN with 4 equiv of CAN and 20 µL H_2O (red), followed by the generation of **3** with 20 equiv of $HClO_4$ to **2** (purple) at 25 °C. **3** goes back to **2** after adding 20 equiv of Ce(III)$(NO_3)_3$ (dark yellow) at 25 °C.

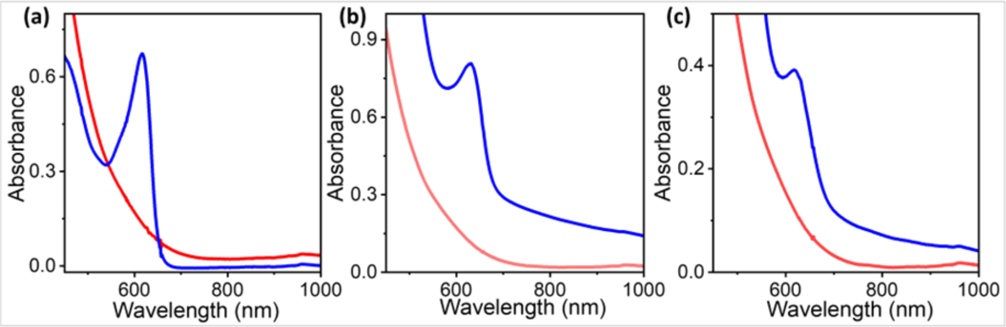

Figure S25. UV-Vis absorption spectral changes depicted the generation of Fc^+ upon adding 5 equiv of Ferrocene derivatives to **2B**. (a) ferrocene (b) acetyl ferrocene, and (c) diacetyl ferrocene at room temperature. Conditions to generate **2B**: 1 mM **1B** + 2 equiv of CAN + 20 µL H_2O at 25 °C in MeCN.

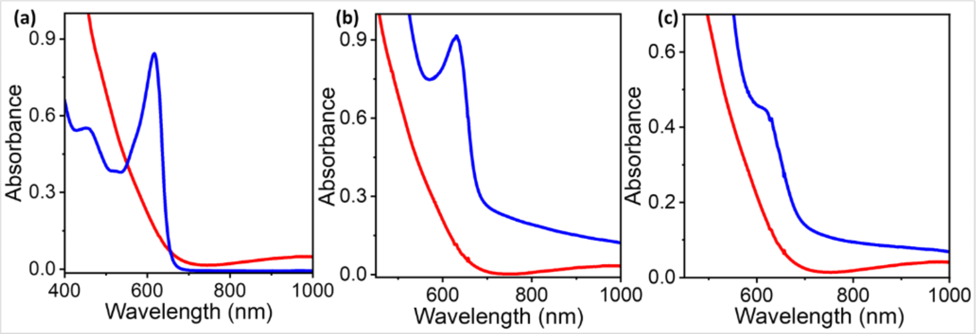

Figure S26. UV-Vis absorption spectral changes depicted the generation of Fc^+ upon adding 5 equiv of Ferrocene derivatives to **2M**. (a) ferrocene (b) acetyl ferrocene, and (c) diacetyl ferrocene at room temperature. Conditions to generate **2M**: 1 mM **1M** + 2 equiv of CAN + 20 µL H_2O at 25 °C in MeCN.

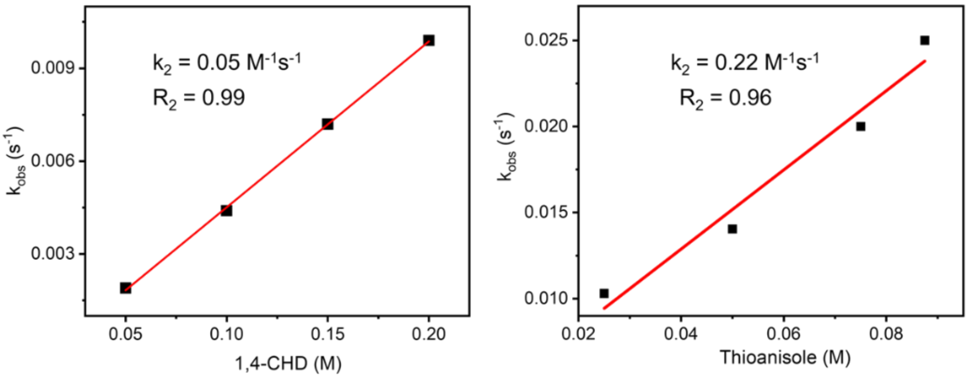

Figure S27. Plot of k_{obs} against the concentration of substrates (left) 1,4 CHD and (right) thioanisole to get the corresponding k_2 of **2B** at 25 °C in CH_3CN. *Condition to generate **2B***: 0.5 mM **1B** in MeCN + 20 µL H_2O + 4 equiv of CAN at 25 °C.

Table S5. Reactivity of **2B** and **2M** towards OAT and HAT reactions at room temperature in MeCN and their comparison with intermediate **4**.[1]

Substrate	**2B** k_2 ($M^{-1}s^{-1}$)	**2M** k_2 ($M^{-1}s^{-1}$)	**4** k_2 ($M^{-1}s^{-1}$)
Thioanisole	0.22	0.4	7.2×10^{-3}
1,4-CHD	5×10^{-2}	2.4×10^{-2}	8.6×10^{-3}

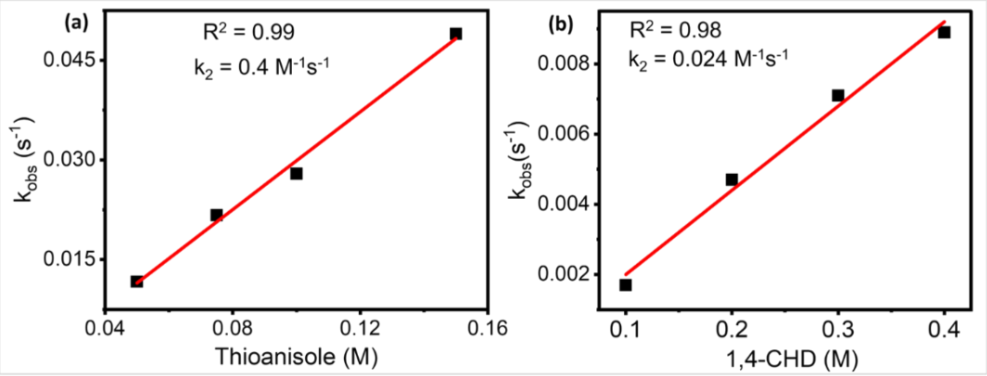

Figure S28. Plot of k_{obs} against the concentration of substrates (a) thioanisole and (b) 1,4 CHD to get the corresponding k_2 of **2M** at 25 °C in CH_3CN. *Condition to generate **2M***: 0.5 mM **1M** in MeCN + 20 µL H_2O + 4 equiv of CAN at 25 °C.

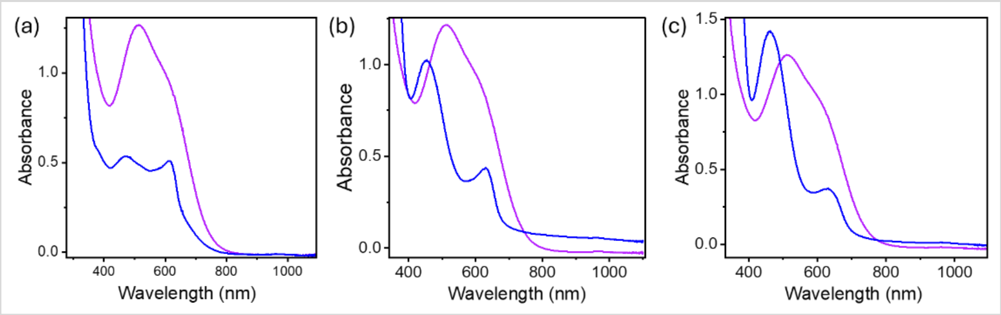

Figure S29. UV-Vis absorption spectral changes depicted the generation of the corresponding ferrocenium ions (blue traces) upon adding 5 equiv of Ferrocene derivatives to **3B** (purple traces). (a) ferrocene (b) acetylferrocene, and (c) diacetylferrocene at room temperature. *Conditions to generate **3B**:* 0.5 mM **1B** + 20 µL H_2O + 2 equiv of CAN + 20 equiv of $HClO_4$ at 25 °C in MeCN.

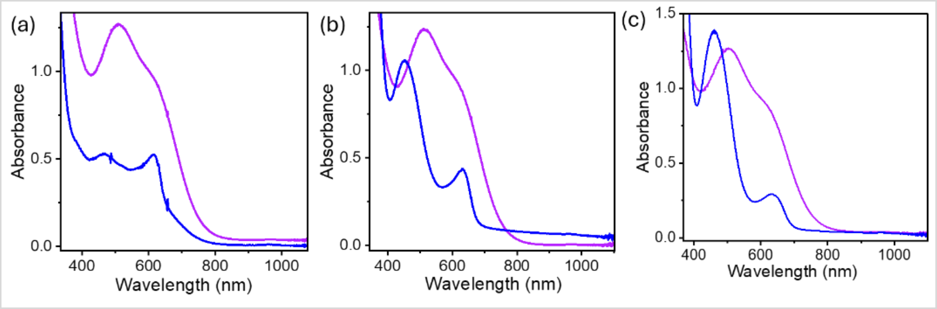

Figure S30. UV-Vis absorption spectral changes depicted the generation of the corresponding ferrocenium ions (blue traces) upon adding 5 equiv of Ferrocene derivatives to **3M** (purple traces). (a) ferrocene (b) acetylferrocene, and (c) diacetylferrocene at room temperature. *Conditions to generate **3M**:* 0.5 mM **1M** + 20 µL H_2O + 2 equiv of CAN + 20 equiv of $HClO_4$ at 25 °C in MeCN.

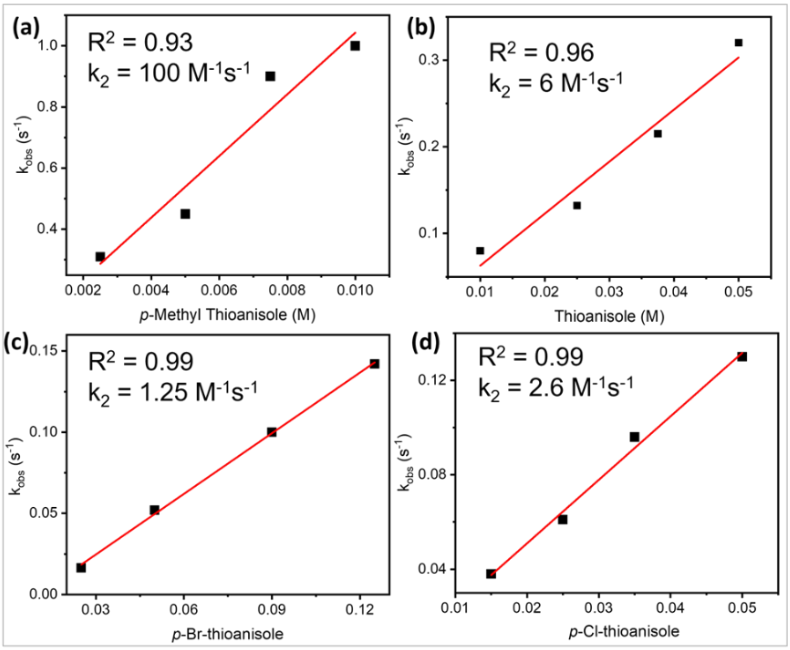

Figure S31. Plot of k_{obs} against the concentration of substrates (a) *p*-methylthioanisole, (b) thioanisole, (c) *p*-Br-thioanisole, and (d) *p*-Cl-thioanisole to get the corresponding k_2 of **3B**. *Condition to generate **3B***: 0.5 mM **1B** in MeCN + 20 µL H_2O + 4 equiv of CAN + 20 equiv $HClO_4$ at -40 °C.

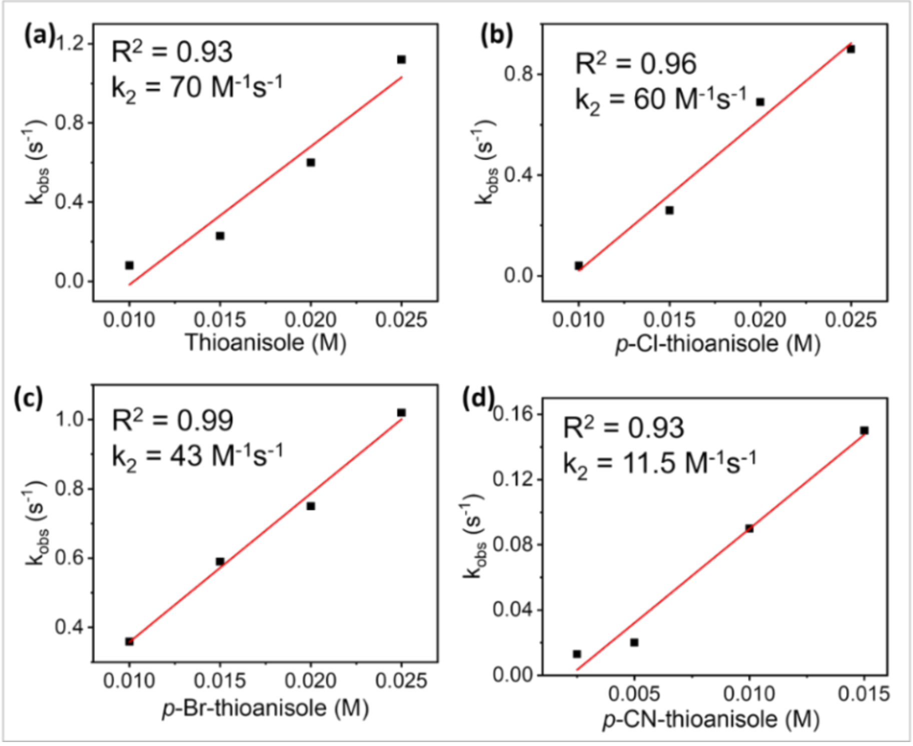

Figure S32. Plot of k_{obs} against the concentration of substrates (a) thioanisole, (b) *p*-Cl-thioanisole, (c) *p*-Br-thioanisole and (d) *p*-CN-thioanisole to get the corresponding k_2 of **3M**. *Condition to generate **3M***: 0.5 mM **1M** in MeCN + 20 µL H_2O + 4 equiv of CAN + 20 equiv $HClO_4$ at -40 °C.

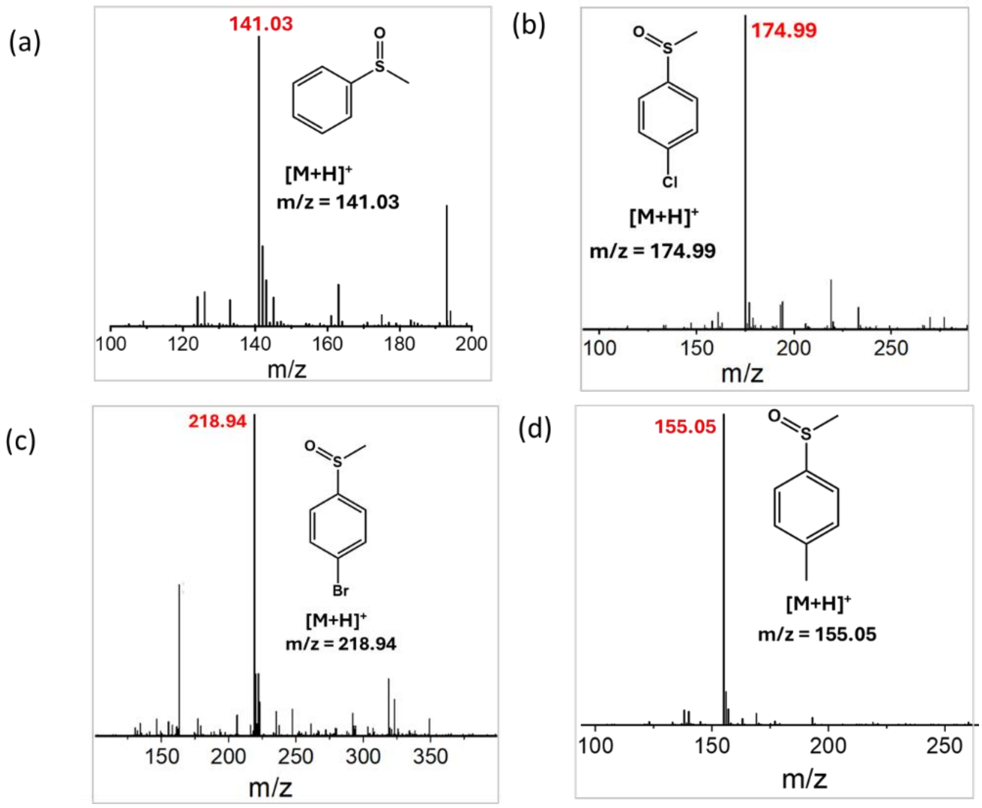

Figure S33. Product analysis of the reaction mixture of **3B** with (a) 50 equiv of thioanisole, (b) 50 equiv of *p*-Cl-thioanisole, (c) 50 equiv of p-Br-thioanisole, and (d) 50 equiv of *p*-Me-thioanisole by ESI-MS. *Conditions to generate **3B***: 1 mM **1B** + 20 µL H_2O + 4 equiv of CAN + 20 equiv of $HClO_4$ in MeCN at 298 K.

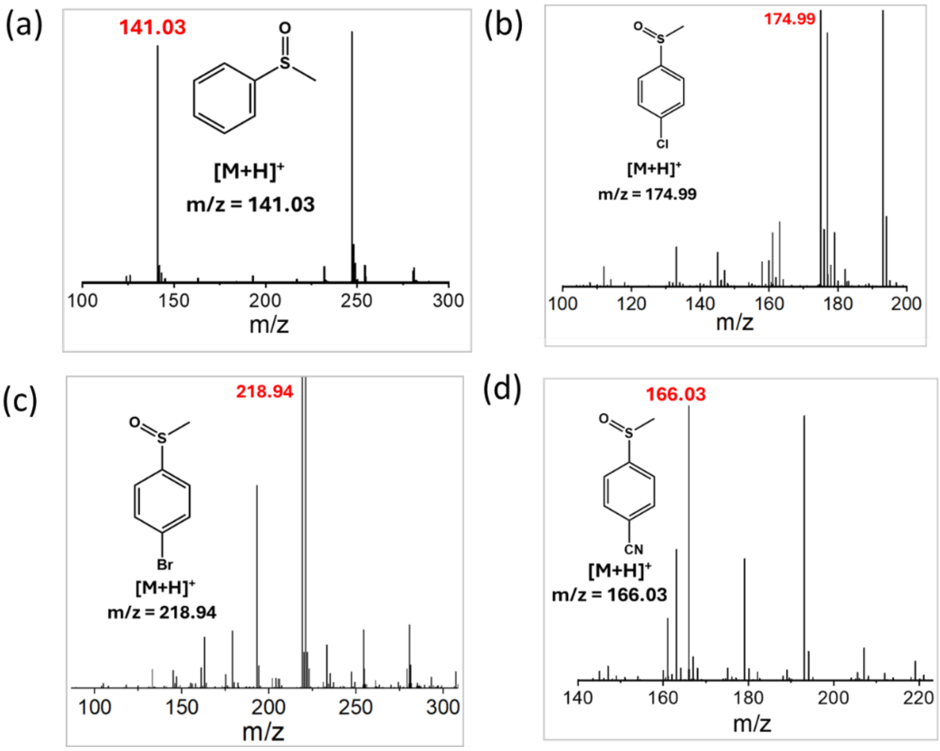

Figure S34. Product analysis of the reaction mixture of **3M** with (a) 50 equiv of thioanisole, (b) 50 equiv of *p*-Cl-thioanisole, (c) 50 equiv of *p*-Br-thioanisole, and (d) 50 equiv of *p*-CN-thioanisole by ESI-MS. *Conditions to generate **3M***: 1 mM **1M** + 20 µL H_2O + 4 equiv of CAN + 20 equiv of $HClO_4$ in MeCN at 298 K.

Table S6. Reactivity of **3B** and **3M** towards OAT reactions and their comparison with $[(BnTPEN)Mn^{IV}{=}O]^{2+}$ (**5**) species.[2]

**Substrate**	**3B** k_2 ($M^{-1}s^{-1}$) at -40 °C	**3M** k_2 ($M^{-1}s^{-1}$) at -40 °C	**5** k_2 ($M^{-1}s^{-1}$) at 0 °C
p-Me-thioanisole	100	–	7.9
thioanisole	6	70	1.3
p-Cl-thioanisole	2.6	60	0.16
p-Br-thioanisole	1.25	43	0.1
p-CN-thioanisole	–	11.5	–

Additional Computational methods:

All geometry optimizations and frequency computations were performed in Gaussian16, with M06L as the functional using the unrestricted formalism and def2-TZVP as the basis set. Single points were computed using M06L functional and def2-TZVPP basis set. Solvent effects were accounted for using integral equation formalism of polarized continuum model as implemented in Gaussian 16. Acetonitrile was used as the solvent for all computations. Ultrafine grid was used for all computations. Stability analysis was performed to verify the stability of the wave function. XQC and YQC algorithms were used when necessary for scf convergence for both geometry optimizations and single points. TDDFT computations were performed using Orca 5.0.4. Unrestricted formalism was used for TDDFT computations. PBE0 functional with ZORA-def2-TZVP basis set and CPCM solvent model with acetonitrile as the solvent were used for TDDFT computations.

Key words used:

For optimizations:

#P M06L def2tzvp opt freq=noraman scrf=(pcm,solvent=acetonitrile)

For stability analysis,

Stable = opt

For single points: #P M06L gfinput IOP(6/7=3) def2tzvpp scrf=(pcm,solvent=acetonitrile) 6D 10F

For TDDFT

! UKS PBE0 ZORA ZORA-DEF2-TZVP SARC/J CPCM(CH3CN) RI-SOMF(1X)

%PAL NPROCS 8 END

%TDDFT NROOTS 10

DoNTO TRUE

NTOSTATES 1,2,3,4,5,6,7,8,9,10

NTOThresh 1e-4

DOSOC FALSE

TDA FALSE

END

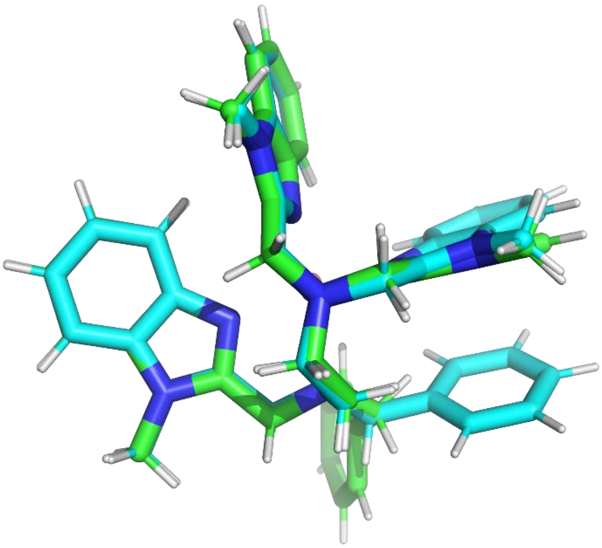

Figure S35. Overlay of the lowest energy structures from two different levels of theory for complex **3B**.

The geometries obtained from three different levels of theory in BP86-D3BJ/def2-TZVP; M06L/6-31+G(d); B97D/def2-TZVP were overlayed in Figure S36 and showed a remarkable agreement with each other indicating little dependence of geometry on the level of theory. Computed TS for the oxygen atom transfer indicates that the electron TBEN framework results in a much later TS for the oxidation of thioanisoles compared to the TPEN framework. However, the key characteristics of the TS remain identical. There is a significant pi stacking interaction between the thioanisole and one of the aromatic groups of the ligand framework. These interactions are affected by the changes in electron density between the imidazole and benzene rings thereby altering the nature of the transition states.

Computed ground state geometries of iron oxo complexes at PCM(CH3CN)-M06L/def2tzvpp/PCM(CH3CN)-M06L/def2svp level of theory are shown in Figure S19. The corresponding Mulliken spins are shown in Table S7.

Table S7. Mulliken spin densities on iron and oxygen atoms computed at PCM(CH_3CN)-M06L/def2tzvpp/PCM(CH_3CN)-M06L/def2svp level of theory.

Molecule	Mulliken spin on Mn	Mulliken Spin on O
[(BnTPEN)Fe^{IV}=O]$^{2+}$	1.27	0.82
[(MeTPEN)Fe^{IV}=O]$^{2+}$	1.28	0.82
[(BnTBEN)Fe^{IV}=O]$^{2+}$	1.26	0.82
[(MeTBEN)Fe^{IV}=O]$^{2+}$	1.25	0.82

Finally, the computed UV/Vis spectra for **3B** and **3M** agree qualitatively with the experimental spectra as indicated in Figure S20.

Section 2: DFT optimization data

The following Tables present the DFT optimized coordinates that were obtained using the BP-86 functional, Def2-TZVP basis set and the atom-pairwise Grimme dispersion correction with the Becke-Johnson damping scheme (D3BJ). All the calculations were performed in gas phase without the polarizable continuum model (CPCM).

Table 5. [(BnTBEN)Mn^{II}(CH_3CN)]$^{2+}$ **(1B)**

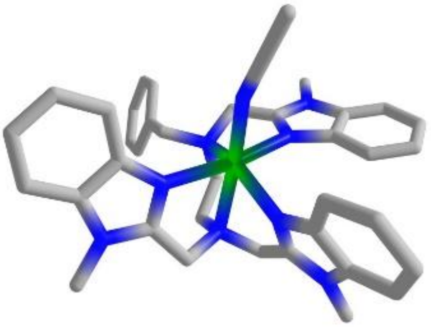

25	8.292584000	8.007419000	3.219944000
7	9.365974000	5.865991000	6.745244000
7	8.336896000	6.716394000	4.947904000
7	10.642455000	7.832663000	3.894508000
7	9.212390000	6.198624000	1.865721000
7	8.608039000	8.395982000	-1.032491000
7	8.913059000	9.899484000	4.100682000
7	8.438395000	8.748692000	1.172861000
7	10.464867000	11.513581000	4.164169000
6	8.108061000	5.260086000	6.687578000
6	6.190502000	5.375645000	5.180220000
6	9.448902000	3.683900000	1.596659000
6	9.449759000	6.725331000	5.687403000
6	5.579505000	4.419376000	5.987586000
6	8.701362000	3.123794000	0.549859000
6	7.472143000	5.801449000	5.545540000
6	11.270591000	6.699959000	3.183064000
6	10.350595000	5.622147000	7.793024000
6	6.220571000	3.895449000	7.129563000
6	10.204073000	10.196329000	3.924690000
6	8.287268000	11.082971000	4.488229000
6	8.903543000	4.831379000	2.407057000
6	10.608411000	7.614101000	5.354543000
6	7.499316000	4.304750000	7.503712000
6	10.666569000	6.481976000	1.804590000
6	9.224472000	2.088598000	-0.226730000
6	9.255109000	12.115805000	4.519653000
6	8.626576000	6.373881000	0.521835000
6	8.486819000	9.777555000	-0.863510000
6	8.565878000	7.832865000	0.208878000
6	8.377791000	9.989071000	0.532564000
6	11.200684000	9.144977000	3.537700000
6	10.725046000	3.164797000	1.863673000
6	6.628425000	12.662937000	5.150568000
6	6.953551000	11.350993000	4.813924000
6	8.463006000	10.822601000	-1.788563000
6	8.934897000	13.433014000	4.853187000
6	8.322263000	12.108674000	-1.268992000
6	8.722769000	7.729104000	-2.324766000
6	10.505264000	1.595763000	0.036949000
6	8.201554000	12.333686000	0.117284000

6	7.599936000	13.685488000	5.166204000
6	11.253039000	2.130495000	1.088902000
6	8.225190000	11.286173000	1.036183000
6	11.738554000	12.214229000	4.048805000
6	5.051378000	8.606954000	2.868817000
7	6.115846000	8.201115000	3.092027000
6	3.725154000	9.116795000	2.597971000
1	11.292718000	6.119131000	7.543248000
1	10.531534000	4.543777000	7.884584000
1	9.987352000	6.010665000	8.753723000
1	12.363135000	6.835171000	3.091784000
1	11.116109000	5.804058000	3.798963000
1	9.288283000	4.796927000	3.432865000
1	7.808574000	4.772239000	2.483142000
1	10.465493000	8.587172000	5.849578000
1	11.561352000	7.197887000	5.725470000
1	10.799572000	7.386910000	1.193922000
1	11.206034000	5.665170000	1.294261000
1	9.182897000	5.812110000	-0.248227000
1	7.606732000	5.956565000	0.538566000
1	11.360912000	9.165709000	2.447955000
1	12.183371000	9.330713000	4.011450000
1	9.556158000	8.163837000	-2.890874000
1	7.794057000	7.851610000	-2.897600000
1	8.912442000	6.661742000	-2.177701000
1	12.537371000	11.498482000	3.832594000
1	11.966850000	12.729895000	4.990202000
1	11.691876000	12.949148000	3.234403000
1	3.255297000	8.534368000	1.793106000
1	3.787985000	10.169106000	2.286648000
1	3.102923000	9.043882000	3.500913000
1	7.687072000	3.481069000	0.356448000
1	8.626793000	1.654002000	-1.028420000
1	10.912278000	0.782518000	-0.564046000
1	12.242217000	1.731133000	1.314071000
1	11.303192000	3.555384000	2.704671000
1	8.106286000	11.471919000	2.101493000
1	8.077915000	13.354937000	0.477620000
1	8.544385000	10.651978000	-2.861609000
1	8.297188000	12.957968000	-1.951179000
1	6.208901000	10.556311000	4.818650000
1	5.601096000	12.909806000	5.417718000
1	7.300696000	14.697536000	5.437217000

1	9.680473000	14.227196000	4.874707000
1	5.693000000	5.781564000	4.300373000
1	4.580356000	4.064336000	5.735812000
1	5.703246000	3.151251000	7.734324000
1	7.988215000	3.895737000	8.387217000

Table 6. $[(BnTBEN)Mn^{III}\text{-O-}Ce^{IV}(NO_3)_4]^+$

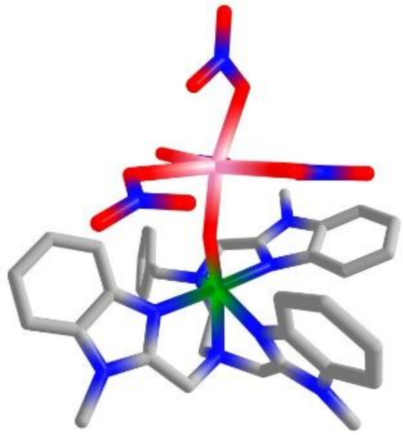

25	8.265950000	8.188847000	3.249646000
7	8.966473000	6.156381000	6.764376000
7	8.155143000	6.943606000	4.841094000
7	10.430452000	7.946277000	3.887213000
7	9.030511000	6.318496000	1.888623000
7	8.202280000	8.673276000	-0.834540000
7	8.903345000	10.069456000	4.214826000
7	8.616464000	8.949411000	1.345160000
7	10.521223000	11.616929000	4.268614000
6	7.701014000	5.578649000	6.598264000
6	5.986287000	5.615627000	4.857229000
6	9.014106000	3.812682000	1.541899000
6	9.190759000	6.954233000	5.687213000
6	5.272601000	4.710346000	5.637306000
6	8.189528000	3.369278000	0.496857000
6	7.203138000	6.069087000	5.367424000
6	11.050034000	6.756774000	3.240053000
6	9.832373000	5.946957000	7.915893000
6	5.758110000	4.254045000	6.878570000
6	10.195259000	10.319141000	4.002352000
6	8.330070000	11.267477000	4.635861000
6	8.598933000	4.978959000	2.400929000

6	10.400513000	7.761126000	5.369533000
6	6.989131000	4.672007000	7.383383000
6	10.495012000	6.499561000	1.850779000
6	8.596134000	2.319052000	-0.327845000
6	9.341959000	12.259715000	4.666427000
6	8.451795000	6.592691000	0.564830000
6	8.259568000	10.050842000	-0.619989000
6	8.413919000	8.064011000	0.354853000
6	8.522459000	10.222379000	0.759771000
6	11.097647000	9.221669000	3.533778000
6	10.239433000	3.164436000	1.759057000
6	6.736109000	12.903543000	5.291323000
6	7.007096000	11.579686000	4.957836000
6	8.090528000	11.119415000	-1.497652000
6	9.074814000	13.586699000	5.007268000
6	8.206775000	12.397307000	-0.956015000
6	7.786424000	8.033726000	-2.075760000
6	9.828845000	1.695566000	-0.116089000
6	8.470388000	12.586071000	0.412106000
6	7.748042000	13.886139000	5.317431000
6	10.648398000	2.114143000	0.934835000
6	8.629053000	11.512819000	1.285606000
6	11.809014000	12.272343000	4.094858000
1	10.801570000	6.425163000	7.742287000
1	9.986983000	4.871688000	8.072687000
1	9.376646000	6.381174000	8.815835000
1	12.147044000	6.866939000	3.203324000
1	10.833615000	5.894502000	3.884784000
1	8.998388000	4.869711000	3.417304000
1	7.506074000	5.024838000	2.493562000
1	10.331009000	8.749844000	5.845418000
1	11.329338000	7.281600000	5.721314000
1	10.711261000	7.354526000	1.195835000
1	10.998816000	5.619739000	1.413106000
1	9.019665000	6.094844000	-0.242838000
1	7.434594000	6.179318000	0.516745000
1	11.207103000	9.253737000	2.438656000
1	12.106105000	9.288331000	3.980298000
1	8.313413000	8.494359000	-2.919618000
1	6.701493000	8.146037000	-2.200841000
1	8.028860000	6.967161000	-2.040306000
1	12.572912000	11.528901000	3.845072000
1	12.097497000	12.781114000	5.023724000

1	11.752827000	13.008767000	3.281738000
1	7.212983000	3.832950000	0.343517000
1	7.941251000	1.976905000	-1.129814000
1	10.142112000	0.871556000	-0.757717000
1	11.599395000	1.613766000	1.120327000
1	10.869525000	3.462596000	2.601419000
1	8.767792000	11.692738000	2.345239000
1	8.516937000	13.599629000	0.808844000
1	7.861605000	10.968570000	-2.551886000
1	8.068017000	13.265685000	-1.598861000
1	6.222859000	10.825080000	4.934744000
1	5.711595000	13.188503000	5.527338000
1	7.485313000	14.910287000	5.581796000
1	9.849257000	14.353027000	5.024102000
1	5.601493000	5.961413000	3.901803000
1	4.306267000	4.357481000	5.279854000
1	5.158322000	3.552968000	7.458372000
1	7.365451000	4.307108000	8.338429000
8	6.534938000	8.361306000	3.087256000
58	4.538075000	8.901473000	2.529367000
7	5.034582000	6.538916000	0.832798000
8	4.329893000	6.494391000	1.907782000
8	5.391252000	7.703805000	0.436080000
8	5.378392000	5.517309000	0.242341000
8	3.841033000	7.544970000	4.477173000
8	4.106026000	8.179891000	6.564121000
7	4.116349000	8.426816000	5.376357000
8	4.424354000	9.594319000	4.907980000
8	5.550424000	10.543605000	0.960215000
8	5.433615000	12.718426000	1.266679000
7	5.208979000	11.587096000	1.636839000
8	4.609641000	11.330705000	2.759837000
8	2.881496000	9.264989000	0.769097000
7	1.840918000	9.315173000	1.549014000
8	2.111121000	9.139015000	2.803881000
8	0.724097000	9.506961000	1.136235000

Table 7. [(BnTBEN)Mn^{III}-O-Ce^{IV}(OH_2)(NO_3)$_4$]$^+$

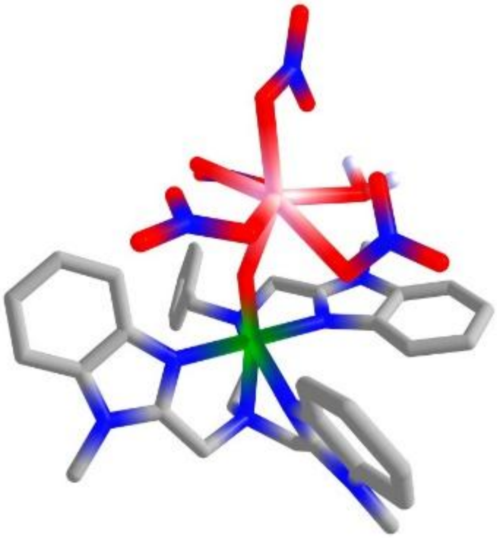

25	8.313591000	8.141343000	3.269525000
7	9.272121000	6.189940000	6.772915000
7	8.311665000	6.976991000	4.919358000
7	10.531188000	7.946531000	3.782498000
7	9.042868000	6.322022000	1.872801000
7	8.422429000	8.705367000	-0.845404000
7	8.983320000	10.034704000	4.236890000
7	8.577969000	8.960083000	1.368612000
7	10.579105000	11.606083000	4.174342000
6	7.983230000	5.643510000	6.728643000
6	6.100707000	5.737954000	5.173475000
6	9.018914000	3.817070000	1.553322000
6	9.417498000	6.973793000	5.672614000
6	5.435886000	4.869574000	6.034362000
6	8.187994000	3.383680000	0.508937000
6	7.385958000	6.137144000	5.544274000
6	11.130048000	6.761080000	3.111684000
6	10.231479000	5.970251000	7.845619000
6	6.029850000	4.398960000	7.222528000
6	10.244654000	10.310980000	3.905542000
6	8.443842000	11.210112000	4.756552000
6	8.621422000	4.990005000	2.411385000
6	10.603748000	7.773731000	5.262798000
6	7.321238000	4.772412000	7.593832000
6	10.504804000	6.487098000	1.756615000

6	8.584030000	2.330119000	-0.317014000
6	9.438936000	12.218069000	4.708574000
6	8.395842000	6.615848000	0.585019000
6	8.498250000	10.077669000	-0.614659000
6	8.463292000	8.084288000	0.359398000
6	8.590583000	10.237498000	0.788101000
6	11.134589000	9.234281000	3.369186000
6	10.238921000	3.158020000	1.766946000
6	6.923691000	12.792200000	5.671489000
6	7.166933000	11.488688000	5.251532000
6	8.463666000	11.153757000	-1.500922000
6	9.196759000	13.528894000	5.124183000
6	8.527815000	12.426929000	-0.938312000
6	8.182595000	8.097834000	-2.145892000
6	9.811093000	1.694573000	-0.108226000
6	8.604164000	12.603841000	0.456270000
6	7.916047000	13.793158000	5.607330000
6	10.636695000	2.104459000	0.941483000
6	8.633348000	11.523715000	1.335325000
6	11.821012000	12.291773000	3.851646000
1	11.193275000	6.421235000	7.581924000
1	10.372480000	4.892921000	8.000905000
1	9.868439000	6.424579000	8.777314000
1	12.222865000	6.880958000	3.016693000
1	10.956505000	5.900342000	3.771774000
1	9.049612000	4.894035000	3.417746000
1	7.531140000	5.038319000	2.530911000
1	10.565047000	8.764751000	5.737219000
1	11.554588000	7.297162000	5.553939000
1	10.695702000	7.330287000	1.078597000
1	10.977667000	5.596921000	1.306243000
1	8.854327000	6.055278000	-0.248340000
1	7.336376000	6.320948000	0.636537000
1	11.162254000	9.259380000	2.269129000
1	12.171904000	9.335151000	3.737587000
1	8.963143000	8.405547000	-2.853352000
1	7.201228000	8.415155000	-2.522314000
1	8.189254000	7.008246000	-2.052508000
1	12.603814000	11.558537000	3.630078000
1	12.139293000	12.900122000	4.707199000
1	11.681724000	12.940528000	2.975723000
1	7.212966000	3.853183000	0.363792000
1	7.923671000	1.993136000	-1.116782000

1	10.114847000	0.867482000	-0.750536000
1	11.583392000	1.595042000	1.124469000
1	10.873950000	3.452360000	2.607100000
1	8.599615000	11.691561000	2.405101000
1	8.600309000	13.613524000	0.865705000
1	8.376527000	11.011418000	-2.577567000
1	8.495343000	13.301001000	-1.588256000
1	6.385560000	10.731173000	5.273136000
1	5.934206000	13.049581000	6.047905000
1	7.673927000	14.802406000	5.939830000
1	9.956747000	14.308067000	5.073422000
1	5.632663000	6.102550000	4.264612000
1	4.423904000	4.555604000	5.781878000
1	5.467015000	3.725252000	7.868227000
1	7.778761000	4.403779000	8.511301000
8	6.584520000	8.049404000	3.071614000
58	4.671859000	8.755604000	2.390732000
8	4.159775000	6.265860000	2.368012000
7	4.850264000	5.966755000	1.336972000
8	5.099796000	4.811759000	1.016886000
8	5.314357000	6.969047000	0.664338000
8	5.438498000	9.496818000	-0.114326000
1	4.844497000	8.860907000	-0.559444000
1	4.955720000	10.346023000	-0.161202000
8	2.939649000	8.594953000	0.621953000
7	1.919724000	8.868354000	1.386725000
8	0.792482000	8.941156000	0.959446000
8	2.222977000	9.046497000	2.624403000
8	3.916874000	7.791584000	4.550717000
8	4.425262000	9.902311000	4.578778000
7	4.083624000	8.852491000	5.258293000
8	3.955799000	8.870993000	6.464673000
8	6.123523000	10.748208000	2.547836000
7	5.278438000	11.650619000	2.164994000
8	4.146444000	11.188698000	1.757019000
8	5.547485000	12.835579000	2.184172000

Table 8. [(BnTBEN)Mn^{IV}-O-$Ce^{IV}(NO_3)_4]^{2+}$ **(2B)**

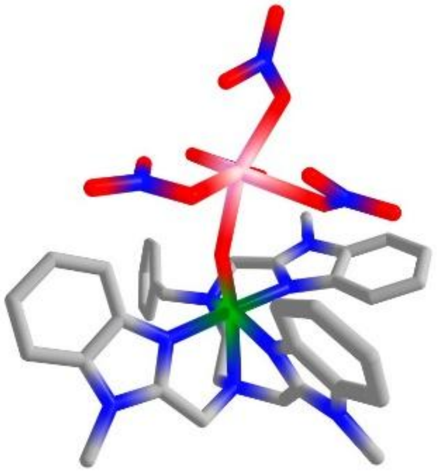

25	8.359283000	8.150995000	3.185150000
7	9.281081000	6.314629000	6.755900000
7	8.370739000	7.071409000	4.865072000
7	10.623837000	7.979273000	3.750526000
7	8.975216000	6.360440000	1.941924000
7	8.316628000	8.607487000	-0.885478000
7	8.823578000	9.874344000	4.012642000
7	8.631898000	8.913243000	1.305001000
7	10.265662000	11.550556000	4.287724000
6	7.976679000	5.801748000	6.705649000
6	6.134233000	5.869471000	5.106024000
6	8.851807000	3.847261000	1.673038000
6	9.471955000	7.056327000	5.642084000
6	5.434543000	5.037465000	5.976273000
6	7.902232000	3.394993000	0.742941000
6	7.411140000	6.272652000	5.497601000
6	11.184628000	6.790569000	3.053716000
6	10.219403000	6.090081000	7.852366000
6	5.993341000	4.600509000	7.194018000
6	10.108107000	10.279063000	3.859310000
6	8.113941000	10.930217000	4.581880000
6	8.556662000	5.034995000	2.544560000
6	10.684909000	7.813850000	5.229350000
6	7.282109000	4.966979000	7.580951000
6	10.458294000	6.490093000	1.758840000
6	8.188505000	2.309330000	-0.085448000
6	9.016785000	12.010435000	4.735842000
6	8.325438000	6.567897000	0.623069000

6	8.450949000	9.984594000	-0.691274000
6	8.413914000	8.016571000	0.318594000
6	8.649545000	10.180183000	0.697371000
6	11.128505000	9.312767000	3.339459000
6	10.080424000	3.175826000	1.775403000
6	6.383109000	12.265363000	5.507640000
6	6.787050000	11.034929000	4.996301000
6	8.390100000	11.036384000	-1.603962000
6	8.616202000	13.242967000	5.252067000
6	8.542140000	12.320668000	-1.088607000
6	7.951942000	7.971425000	-2.148466000
6	9.423430000	1.661361000	0.010055000
6	8.728522000	12.533429000	0.289512000
6	7.276199000	13.350155000	5.624140000
6	10.367546000	2.089859000	0.947365000
6	8.780972000	11.479124000	1.196810000
6	11.478268000	12.363946000	4.237522000
1	11.197293000	6.510086000	7.598224000
1	10.325735000	5.012515000	8.028231000
1	9.848123000	6.572524000	8.765793000
1	12.263020000	6.914332000	2.861099000
1	11.078197000	5.942736000	3.744138000
1	9.066977000	4.954167000	3.511257000
1	7.481782000	5.127153000	2.743128000
1	10.681716000	8.809413000	5.699593000
1	11.618712000	7.313343000	5.531345000
1	10.631437000	7.291486000	1.030340000
1	10.848807000	5.557896000	1.320355000
1	8.798869000	5.944538000	-0.151547000
1	7.283419000	6.239483000	0.694651000
1	11.174926000	9.347585000	2.240447000
1	12.137761000	9.519304000	3.733031000
1	8.624806000	8.318823000	-2.941018000
1	6.912963000	8.225673000	-2.393496000
1	8.032216000	6.884994000	-2.051784000
1	12.333679000	11.740644000	3.960687000
1	11.659237000	12.810608000	5.222581000
1	11.358750000	13.159860000	3.491020000
1	6.924381000	3.877879000	0.677041000
1	7.439668000	1.958581000	-0.795698000
1	9.643230000	0.809700000	-0.633885000
1	11.319289000	1.567048000	1.043097000
1	10.806993000	3.479526000	2.533846000

1	8.831811000	11.689465000	2.258655000
1	8.794524000	13.554744000	0.662465000
1	8.220372000	10.866900000	-2.666458000
1	8.492794000	13.176232000	-1.761016000
1	6.101500000	10.191847000	4.943688000
1	5.347941000	12.389325000	5.822597000
1	6.912150000	14.296251000	6.023286000
1	9.304457000	14.080063000	5.362573000
1	5.698068000	6.197748000	4.167170000
1	4.426003000	4.725488000	5.708431000
1	5.406617000	3.955540000	7.847300000
1	7.714573000	4.616001000	8.517150000
8	6.662187000	8.184471000	3.039910000
58	4.528017000	8.903080000	2.372755000
7	5.050115000	6.896562000	0.317210000
8	4.318991000	6.679552000	1.357976000
8	5.508015000	8.095394000	0.204035000
8	5.328272000	6.015719000	-0.479818000
8	3.647997000	7.255467000	3.966875000
8	3.396970000	7.645524000	6.116954000
7	3.715813000	8.012935000	5.014675000
8	4.180668000	9.203969000	4.774952000
8	5.877826000	10.681624000	1.391631000
8	5.795478000	12.785895000	2.030299000
7	5.393155000	11.649547000	2.108584000
8	4.464143000	11.284979000	2.930560000
8	3.117208000	9.620835000	0.581566000
7	2.002165000	9.660880000	1.267928000
8	2.146949000	9.270643000	2.497643000
8	0.959464000	10.020166000	0.801324000

Table 9. [(BnTBEN)Mn^{IV}-O-$Ce^{IV}(OH_2)(NO_3)_4]^{2+}$

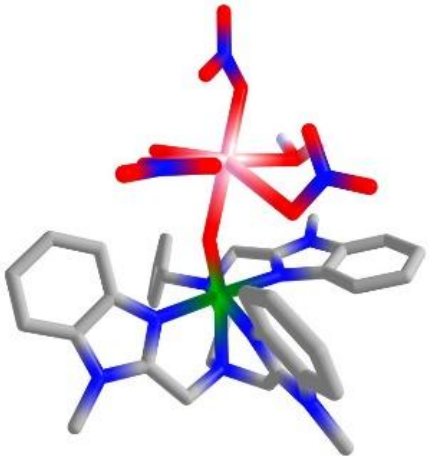

25	8.438433000	8.162450000	3.187917000
7	9.455913000	6.292818000	6.711404000
7	8.483536000	7.083895000	4.865428000
7	10.713819000	7.938196000	3.662264000
7	8.987339000	6.397328000	1.874513000
7	8.450227000	8.736126000	-0.892258000
7	8.975936000	9.873368000	4.023890000
7	8.634632000	8.982089000	1.318392000
7	10.490585000	11.487982000	4.291643000
6	8.133059000	5.827629000	6.725113000
6	6.203666000	5.990093000	5.239921000
6	8.905000000	3.883211000	1.606001000
6	9.618377000	7.030792000	5.591459000
6	5.520755000	5.188776000	6.151273000
6	8.044081000	3.444520000	0.588132000
6	7.523445000	6.323449000	5.548065000
6	11.227822000	6.742972000	2.944770000
6	10.435744000	6.033529000	7.762421000
6	6.130384000	4.716941000	7.330290000
6	10.262326000	10.244689000	3.813980000
6	8.341695000	10.923226000	4.686486000
6	8.547565000	5.069924000	2.456563000
6	10.830533000	7.763945000	5.135765000
6	7.455593000	5.022833000	7.640106000
6	10.468823000	6.488938000	1.661862000
6	8.399179000	2.358136000	-0.213131000
6	9.290975000	11.961438000	4.843546000

6	8.326035000	6.660457000	0.570136000
6	8.573148000	10.106748000	-0.654933000
6	8.473916000	8.111185000	0.302409000
6	8.678135000	10.265544000	0.748487000
6	11.229994000	9.261010000	3.232976000
6	10.112047000	3.199345000	1.821279000
6	6.716377000	12.268295000	5.777355000
6	7.041673000	11.061763000	5.167229000
6	8.568371000	11.182480000	-1.542642000
6	8.970025000	13.175836000	5.452275000
6	8.669045000	12.453659000	-0.983142000
6	8.214802000	8.138058000	-2.201639000
6	9.613277000	1.697826000	-0.004950000
6	8.748359000	12.630348000	0.411283000
6	7.660753000	13.307466000	5.913446000
6	10.467573000	2.113519000	1.020200000
6	8.750779000	11.552432000	1.293120000
6	11.727609000	12.260384000	4.213736000
1	11.412500000	6.429879000	7.469005000
1	10.520625000	4.951910000	7.924958000
1	10.117997000	6.516671000	8.695515000
1	12.306589000	6.836003000	2.736546000
1	11.104376000	5.889857000	3.625563000
1	9.010346000	4.992711000	3.447322000
1	7.463669000	5.154205000	2.603201000
1	10.864751000	8.756198000	5.611542000
1	11.765029000	7.242920000	5.398359000
1	10.649305000	7.302162000	0.947740000
1	10.824238000	5.556572000	1.195306000
1	8.761360000	6.032118000	-0.222041000
1	7.254654000	6.417050000	0.652666000
1	11.221072000	9.300572000	2.133538000
1	12.262606000	9.440698000	3.575917000
1	9.027569000	8.409324000	-2.886613000
1	7.260423000	8.503182000	-2.601970000
1	8.168139000	7.049344000	-2.108673000
1	12.530508000	11.638210000	3.807558000
1	12.007431000	12.608396000	5.215600000
1	11.580007000	13.125584000	3.554759000
1	7.075788000	3.928257000	0.447054000
1	7.716696000	2.013681000	-0.990377000
1	9.884362000	0.845011000	-0.627509000
1	11.401095000	1.581411000	1.203943000

1	10.767195000	3.495115000	2.645426000
1	8.714025000	11.729380000	2.361917000
1	8.770705000	13.641640000	0.815783000
1	8.480279000	11.041013000	-2.619257000
1	8.662464000	13.327137000	-1.634178000
1	6.309098000	10.272852000	5.042264000
1	5.703322000	12.412712000	6.151054000
1	7.360318000	14.238153000	6.393772000
1	9.696305000	13.979523000	5.567281000
1	5.719606000	6.348357000	4.338495000
1	4.483875000	4.927652000	5.943404000
1	5.555216000	4.095881000	8.016326000
1	7.926862000	4.651271000	8.549192000
8	6.746392000	8.145544000	3.071184000
58	4.622395000	8.799746000	2.304581000
8	4.144447000	6.344715000	2.564201000
7	4.793000000	5.935511000	1.540787000
8	4.967423000	4.760065000	1.284662000
8	5.297105000	6.879883000	0.802186000
8	5.427755000	9.312392000	-0.222789000
1	4.869512000	8.648242000	-0.673558000
1	4.976959000	10.162385000	-0.397198000
8	2.940130000	8.480909000	0.582045000
7	1.897256000	8.742525000	1.330864000
8	0.773788000	8.720655000	0.911707000
8	2.208794000	9.020686000	2.551840000
8	4.007229000	8.026602000	4.551891000
8	4.176821000	10.174846000	4.305948000
7	3.937542000	9.188863000	5.113159000
8	3.700535000	9.343538000	6.286707000
8	6.130068000	10.725636000	2.362128000
7	5.299404000	11.625887000	1.923693000
8	4.169076000	11.149339000	1.526834000
8	5.581997000	12.800947000	1.890409000

Table 10. [(BnTBEN)Mn^{IV}(O)]$^{2+}$ **(3B)**

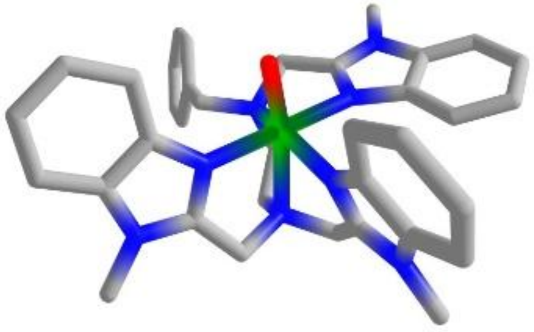

25	8.569589000	7.905550000	3.002490000
7	9.282880000	6.044355000	6.599846000
7	8.460609000	6.900700000	4.711892000
7	10.782086000	7.834491000	3.728875000
7	9.333239000	6.173500000	1.808523000
7	8.773854000	8.357966000	-1.095555000
7	8.850359000	9.657587000	3.806071000
7	8.890384000	8.708939000	1.108191000
7	10.160511000	11.374440000	4.341563000
6	7.960899000	5.589488000	6.496295000
6	6.136550000	5.863222000	4.890275000
6	9.377337000	3.650814000	1.581626000
6	9.530761000	6.822544000	5.518386000
6	5.379770000	5.036155000	5.717055000
6	8.510360000	3.129413000	0.609174000
6	7.445431000	6.136988000	5.297929000
6	11.458192000	6.714473000	3.019787000
6	10.176859000	5.749262000	7.715131000
6	5.901451000	4.495312000	6.910596000
6	10.119752000	10.107327000	3.859367000
6	8.017379000	10.674176000	4.258666000
6	8.970119000	4.837786000	2.410959000
6	10.759545000	7.601364000	5.197669000
6	7.204786000	4.761705000	7.326852000
6	10.807908000	6.386994000	1.685818000
6	8.901310000	2.051113000	-0.185888000
6	8.839837000	11.773570000	4.601794000
6	8.696273000	6.348455000	0.478965000
6	8.834937000	9.741987000	-0.917385000
6	8.795445000	7.792465000	0.136002000
6	8.896577000	9.960989000	0.482560000

6	11.240271000	9.209970000	3.420855000
6	10.633695000	3.051060000	1.759403000
6	6.096647000	11.931593000	4.852144000
6	6.627529000	10.736666000	4.374389000
6	8.830526000	10.781412000	-1.847946000
6	8.307665000	12.972483000	5.080043000
6	8.881733000	12.076047000	-1.337480000
6	8.657943000	7.686644000	-2.386141000
6	10.164208000	1.477332000	-0.014586000
6	8.922214000	12.312571000	0.050313000
6	6.919566000	13.025063000	5.199801000
6	11.028034000	1.972724000	0.965359000
6	8.928716000	11.271261000	0.975700000
6	11.333821000	12.224323000	4.522721000
8	6.968322000	7.716106000	2.649712000
1	12.196084000	9.460240000	3.911859000
1	11.351861000	12.610908000	5.549146000
1	12.243504000	11.644469000	4.340793000
1	11.298994000	13.064913000	3.817712000
1	11.386399000	9.295212000	2.332875000
1	8.932339000	13.824952000	5.344041000
1	6.455892000	13.939588000	5.568532000
1	5.016697000	12.031428000	4.957218000
1	6.008872000	9.887646000	4.084434000
1	11.681636000	7.103955000	5.538096000
1	10.712481000	8.575901000	5.709794000
1	11.391568000	5.841187000	3.682778000
1	12.531095000	6.921041000	2.872001000
1	10.966501000	7.209889000	0.978150000
1	11.282645000	5.489708000	1.256374000
1	7.883385000	4.869511000	2.563813000
1	9.438725000	4.801096000	3.401874000
1	7.510198000	3.552298000	0.491249000
1	8.214168000	1.646331000	-0.929229000
1	10.467112000	0.630755000	-0.630853000
1	12.002400000	1.509010000	1.120355000
1	11.300690000	3.407452000	2.548837000
1	9.155964000	5.693940000	-0.277998000
1	7.636119000	6.069060000	0.580153000
1	8.726100000	6.603698000	-2.246455000
1	9.470525000	8.013022000	-3.047130000
1	7.692218000	7.927347000	-2.849301000
1	8.910453000	11.486885000	2.039223000

1	8.931369000	13.341304000	0.409968000
1	8.875185000	12.921743000	-2.024187000
1	8.779563000	10.596615000	-2.920395000
1	11.182963000	6.118365000	7.493654000
1	9.809827000	6.231961000	8.630380000
1	10.221607000	4.664315000	7.871062000
1	7.600412000	4.344625000	8.252162000
1	5.269089000	3.855578000	7.525546000
1	4.352310000	4.803140000	5.438407000
1	5.745712000	6.299639000	3.973168000

Table 11. $[(BnTBEN)Mn^{IV}(OH)]^{3+}$

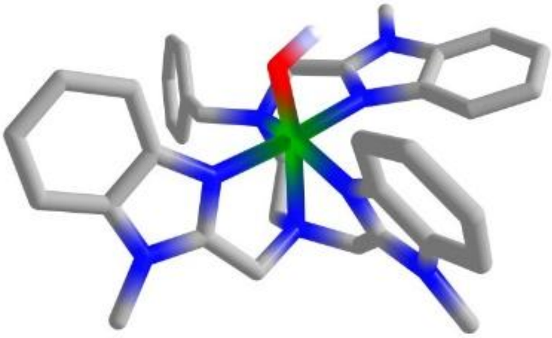

25	8.610340000	7.933050000	2.988747000
7	9.353374000	6.094780000	6.581420000
7	8.496421000	6.907526000	4.687051000
7	10.806533000	7.857540000	3.676529000
7	9.365633000	6.136731000	1.807712000
7	8.806699000	8.304725000	-1.092802000
7	8.919726000	9.688101000	3.796855000
7	8.819357000	8.659614000	1.115134000
7	10.261543000	11.403964000	4.263324000
6	8.041108000	5.605084000	6.489500000
6	6.197746000	5.789876000	4.896318000
6	9.425768000	3.615348000	1.604109000
6	9.580686000	6.861500000	5.497725000
6	5.473902000	4.953850000	5.745925000
6	8.539617000	3.066262000	0.661863000
6	7.501024000	6.120043000	5.286051000
6	11.489498000	6.711042000	3.001236000
6	10.260408000	5.828878000	7.700023000
6	6.019991000	4.453396000	6.944016000

6	10.199741000	10.145957000	3.782107000
6	8.112171000	10.697475000	4.319562000
6	9.012721000	4.794838000	2.435115000
6	10.788597000	7.661569000	5.159511000
6	7.319795000	4.770274000	7.341565000
6	10.845418000	6.360672000	1.676578000
6	8.926091000	1.979635000	-0.122548000
6	8.957010000	11.797326000	4.609932000
6	8.743975000	6.284775000	0.463653000
6	8.803954000	9.695013000	-0.910785000
6	8.801270000	7.731554000	0.123591000
6	8.796553000	9.920553000	0.488048000
6	11.294600000	9.225566000	3.334104000
6	10.698545000	3.040260000	1.760332000
6	6.244494000	11.948165000	5.090290000
6	6.736977000	10.757249000	4.559924000
6	8.791817000	10.728583000	-1.846166000
6	8.464324000	12.990733000	5.138683000
6	8.760831000	12.026843000	-1.339182000
6	8.782679000	7.639392000	-2.398296000
6	10.203459000	1.429514000	0.026522000
6	8.728643000	12.268251000	0.047165000
6	7.089367000	13.042231000	5.372261000
6	11.086847000	1.954180000	0.975705000
6	8.743701000	11.230660000	0.979034000
6	11.445927000	12.255441000	4.399876000
8	6.837029000	7.859747000	2.781415000
1	12.258968000	9.448391000	3.817981000
1	11.554497000	12.567036000	5.446011000
1	12.338608000	11.706111000	4.087348000
1	11.332215000	13.142416000	3.764074000
1	11.439848000	9.283754000	2.244613000
1	9.108721000	13.839495000	5.365491000
1	6.657443000	13.952289000	5.787921000
1	5.178309000	12.038981000	5.296430000
1	6.090014000	9.906828000	4.348809000
1	11.724546000	7.191342000	5.497197000
1	10.725793000	8.647487000	5.646568000
1	11.420262000	5.856121000	3.686436000
1	12.560572000	6.925229000	2.859674000
1	11.000499000	7.166820000	0.948524000
1	11.311067000	5.451333000	1.264259000
1	7.927072000	4.819022000	2.593815000

1	9.492911000	4.779311000	3.420434000
1	7.526831000	3.465114000	0.566545000
1	8.225704000	1.546859000	-0.836965000
1	10.503288000	0.576256000	-0.582053000
1	12.069191000	1.503025000	1.115430000
1	11.378771000	3.412620000	2.531067000
1	9.249482000	5.647671000	-0.277395000
1	7.698640000	5.946278000	0.530383000
1	9.623888000	7.997875000	-3.003908000
1	8.868186000	6.557216000	-2.265689000
1	7.839036000	7.866867000	-2.910413000
1	8.665308000	11.448925000	2.039464000
1	8.672733000	13.297255000	0.401579000
1	8.744902000	12.869280000	-2.029926000
1	8.796174000	10.539871000	-2.919201000
1	10.335042000	4.745968000	7.856558000
1	9.873545000	6.303468000	8.610983000
1	11.254141000	6.229486000	7.479024000
1	7.736852000	4.384301000	8.271094000
1	5.414279000	3.805424000	7.576945000
1	4.453161000	4.682076000	5.477970000
1	5.765849000	6.181109000	3.979119000
1	6.468298000	8.372477000	2.034480000

Table 12. [(MeTBEN)Mn^{II}(CH_3CN)]$^{2+}$ (**1M**)

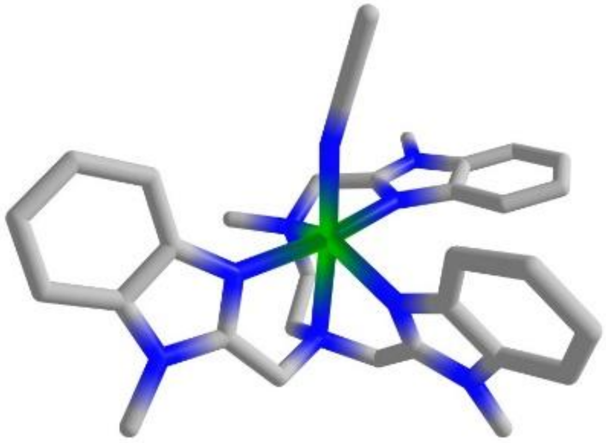

25	8.320250000	8.009922000	3.226263000
7	9.365734000	5.880391000	6.764490000
7	8.367181000	6.692999000	4.932364000
7	10.671468000	7.837201000	3.917144000

7	9.277234000	6.186378000	1.869714000
7	8.590800000	8.367191000	-1.037976000
7	8.935810000	9.901919000	4.101725000
7	8.464755000	8.718068000	1.170860000
7	10.486570000	11.515834000	4.191554000
6	8.114935000	5.261744000	6.689931000
6	6.228448000	5.335598000	5.141289000
6	9.463761000	6.724120000	5.695994000
6	5.608369000	4.387781000	5.951831000
6	7.498555000	5.778588000	5.525936000
6	11.313921000	6.704349000	3.219919000
6	10.329866000	5.665463000	7.837846000
6	6.229739000	3.888599000	7.115597000
6	10.229194000	10.200049000	3.942662000
6	8.305203000	11.083621000	4.488336000
6	9.007084000	4.824137000	2.367589000
6	10.619470000	7.621729000	5.377271000
6	7.497106000	4.314854000	7.509084000
6	10.729207000	6.479980000	1.832785000
6	9.272437000	12.116232000	4.535512000
6	8.707843000	6.348890000	0.518399000
6	8.427953000	9.744335000	-0.867449000
6	8.598465000	7.805929000	0.204358000
6	8.347035000	9.955050000	0.530320000
6	11.231220000	9.150486000	3.565130000
6	6.639259000	12.660510000	5.139896000
6	6.967910000	11.350235000	4.800359000
6	8.344436000	10.785450000	-1.793463000
6	8.948797000	13.431795000	4.872110000
6	8.171953000	12.066875000	-1.272313000
6	8.690138000	7.702712000	-2.333106000
6	8.079374000	12.290871000	0.116324000
6	7.610548000	13.682877000	5.171662000
6	8.163257000	11.247256000	1.036012000
6	11.761346000	12.217897000	4.094889000
7	6.145004000	8.200959000	3.105797000
1	11.274895000	6.160451000	7.595191000
1	10.513411000	4.590413000	7.957824000
1	9.945296000	6.074499000	8.781548000
1	12.406878000	6.843673000	3.141651000
1	11.156343000	5.809548000	3.836360000
1	9.413251000	4.703912000	3.376658000
1	7.923486000	4.667472000	2.429698000

1	10.461636000	8.594896000	5.867550000
1	11.570621000	7.214621000	5.762773000
1	10.870427000	7.382266000	1.219643000
1	11.281404000	5.659586000	1.336584000
1	9.304762000	5.813935000	-0.243708000
1	7.705312000	5.891378000	0.512620000
1	11.401917000	9.171624000	2.476954000
1	12.209031000	9.337836000	4.047996000
1	9.504902000	8.150224000	-2.916178000
1	7.748058000	7.811624000	-2.886452000
1	8.897563000	6.637868000	-2.192176000
1	12.564102000	11.503354000	3.889685000
1	11.975145000	12.732896000	5.039989000
1	11.725177000	12.953755000	3.280833000
1	8.067752000	11.431986000	2.103770000
1	7.930669000	13.308149000	0.478304000
1	8.404948000	10.615358000	-2.867948000
1	8.100046000	12.913145000	-1.954917000
1	6.222494000	10.556449000	4.790952000
1	5.608867000	12.906163000	5.396088000
1	7.308372000	14.693613000	5.444281000
1	9.694050000	14.225853000	4.905397000
1	5.746218000	5.722445000	4.244467000
1	4.617673000	4.019844000	5.685575000
1	5.705917000	3.150351000	7.722042000
1	7.970596000	3.924918000	8.409449000
1	9.447304000	4.053747000	1.709870000
6	5.058673000	8.547993000	2.889874000
6	3.705375000	8.987952000	2.630949000
1	3.123222000	8.977800000	3.563133000
1	3.717326000	10.008911000	2.223971000
1	3.224614000	8.319112000	1.903362000

Table 13. $[(MeTBEN)Mn^{III}\text{-}O\text{-}Ce^{IV}(NO_3)_4]^+$

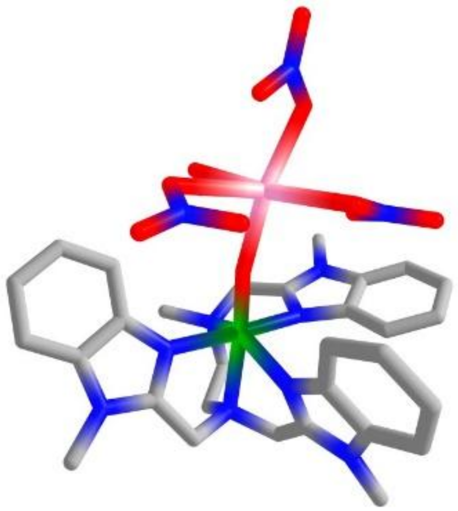

25	7.989643000	8.135206000	3.269305000
7	8.799183000	6.152635000	6.785913000
7	7.937038000	6.895265000	4.867102000
7	10.172996000	7.924341000	3.851256000
7	8.756406000	6.245566000	1.896893000
7	7.797755000	8.558033000	-0.822940000
7	8.631155000	10.029763000	4.206558000
7	8.278871000	8.861291000	1.339809000
7	10.222383000	11.605322000	4.157039000
6	7.537017000	5.557904000	6.658081000
6	5.781028000	5.550379000	4.957116000
6	8.990298000	6.934048000	5.690483000
6	5.092252000	4.656093000	5.771451000
6	7.007208000	6.019800000	5.429292000
6	10.793835000	6.734409000	3.208379000
6	9.691680000	5.977229000	7.922991000
6	5.610230000	4.226995000	7.009360000
6	9.909332000	10.296043000	3.937412000
6	8.054092000	11.228310000	4.622099000
6	8.403959000	4.902971000	2.388471000
6	10.179729000	7.756316000	5.336363000
6	6.850005000	4.662281000	7.477298000
6	10.215167000	6.456818000	1.831251000
6	9.047955000	12.238660000	4.583768000
6	8.140202000	6.494511000	0.585489000
6	7.841910000	9.938383000	-0.623133000

6	8.058296000	7.963412000	0.363963000
6	8.145262000	10.126866000	0.745495000
6	10.814748000	9.203083000	3.464447000
6	6.461635000	12.852185000	5.310311000
6	6.742427000	11.524529000	5.000662000
6	7.631570000	10.995479000	-1.505822000
6	8.770843000	13.569812000	4.899250000
6	7.747861000	12.279972000	-0.980266000
6	7.355731000	7.904166000	-2.047533000
6	8.050225000	12.485836000	0.377486000
6	7.453414000	13.853933000	5.259590000
6	8.248780000	11.423410000	1.256035000
6	11.488591000	12.277811000	3.906272000
1	10.655807000	6.451142000	7.713202000
1	9.851874000	4.907005000	8.105845000
1	9.255969000	6.435893000	8.820736000
1	11.888896000	6.854548000	3.150467000
1	10.597427000	5.876862000	3.865459000
1	8.828241000	4.742058000	3.385770000
1	7.314265000	4.821373000	2.470032000
1	10.104546000	8.749214000	5.802241000
1	11.124047000	7.295935000	5.672112000
1	10.407723000	7.308264000	1.164298000
1	10.727312000	5.577721000	1.396191000
1	8.712817000	6.010068000	-0.229986000
1	7.134729000	6.046640000	0.563406000
1	10.900693000	9.220173000	2.366907000
1	11.832027000	9.290295000	3.887197000
1	7.885295000	8.333671000	-2.906422000
1	6.272987000	8.041837000	-2.165715000
1	7.569528000	6.832560000	-1.992820000
1	12.265081000	11.537433000	3.687751000
1	11.786000000	12.852794000	4.792419000
1	11.394270000	12.957487000	3.048358000
1	8.417280000	11.615451000	2.309007000
1	8.095325000	13.503894000	0.762679000
1	7.372665000	10.830494000	-2.550939000
1	7.578289000	13.139896000	-1.627052000
1	5.974880000	10.753651000	5.048570000
1	5.444481000	13.123992000	5.589724000
1	7.183027000	14.880433000	5.506548000
1	9.530454000	14.350076000	4.861451000
1	5.371635000	5.877315000	4.004955000

1	4.119503000	4.291003000	5.444949000
1	5.028700000	3.534200000	7.617065000
1	7.250068000	4.319690000	8.430927000
1	8.772398000	4.112547000	1.710338000
8	6.256581000	8.305426000	3.162029000
58	4.232487000	8.809332000	2.660374000
8	3.609547000	7.482019000	4.653614000
7	3.897783000	8.386491000	5.525489000
8	3.926999000	8.165473000	6.717919000
8	4.174087000	9.548151000	5.023002000
8	2.519471000	9.110850000	0.947997000
8	4.296418000	11.244195000	2.840021000
7	4.850287000	11.479986000	1.690183000
8	5.046874000	12.604235000	1.284374000
8	5.178869000	10.423792000	1.025851000
8	4.028342000	6.387272000	2.122233000
7	4.737711000	6.396592000	1.048619000
8	5.049099000	7.550546000	0.586022000
8	5.128222000	5.356496000	0.524464000
8	1.817655000	9.068443000	3.011054000
7	1.504862000	9.191196000	1.760095000
8	0.374600000	9.363395000	1.376883000

Table 14. $[(MeTBEN)Mn^{III}\text{-}O\text{-}Ce^{IV}(OH_2)(NO_3)_4]^+$

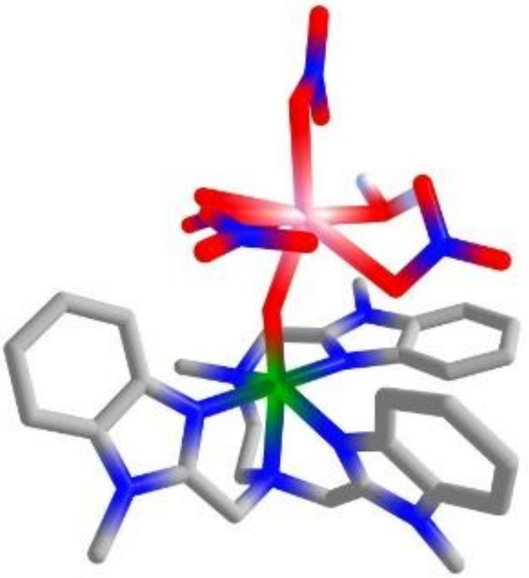

25	8.262822000	8.136369000	3.246642000
7	9.167743000	6.163109000	6.752064000
7	8.235496000	6.949730000	4.883860000
7	10.472240000	7.878512000	3.753458000
7	8.953925000	6.277906000	1.833639000
7	8.251887000	8.675150000	-0.871185000
7	8.987049000	10.010109000	4.212877000
7	8.535649000	8.920650000	1.331197000
7	10.628647000	11.533721000	4.148315000
6	7.865269000	5.649668000	6.709231000
6	5.990310000	5.777754000	5.146191000
6	9.337212000	6.928884000	5.642570000
6	5.298216000	4.941658000	6.017704000
6	7.285939000	6.142898000	5.515587000
6	11.042954000	6.677635000	3.087212000
6	10.116344000	5.933848000	7.832459000
6	5.875173000	4.469808000	7.213703000
6	10.255198000	10.250420000	3.877387000
6	8.486464000	11.198489000	4.743282000
6	8.549558000	4.950233000	2.326679000
6	10.540596000	7.703727000	5.234110000
6	7.176046000	4.809836000	7.584084000
6	10.417646000	6.423474000	1.727619000
6	9.511094000	12.176312000	4.693831000
6	8.317567000	6.575942000	0.541984000
6	8.350052000	10.046459000	-0.641234000
6	8.361415000	8.049047000	0.326014000
6	8.522925000	10.200212000	0.753901000
6	11.111636000	9.148675000	3.338148000
6	7.026926000	12.817156000	5.692077000
6	7.224731000	11.510858000	5.256943000
6	8.270702000	11.125775000	-1.520229000
6	9.314460000	13.490092000	5.124151000
6	8.373217000	12.396173000	-0.957224000
6	7.932313000	8.073069000	-2.157075000
6	8.531626000	12.567424000	0.431255000
6	8.048104000	13.788299000	5.624640000
6	8.606055000	11.483654000	1.302856000
6	11.895946000	12.180166000	3.843771000
1	11.090888000	6.355124000	7.566305000
1	10.228399000	4.855563000	8.003716000
1	9.761477000	6.411014000	8.755825000
1	12.139130000	6.767804000	2.998992000

1	10.840639000	5.821564000	3.744625000
1	8.982837000	4.770462000	3.317121000
1	7.458447000	4.915171000	2.419812000
1	10.521110000	8.695368000	5.708305000
1	11.481563000	7.208132000	5.525876000
1	10.630314000	7.264428000	1.052914000
1	10.878634000	5.522878000	1.279716000
1	8.810904000	6.037247000	-0.289493000
1	7.269713000	6.243060000	0.568564000
1	11.135838000	9.172070000	2.237962000
1	12.152315000	9.218580000	3.703626000
1	8.683210000	8.360289000	-2.904250000
1	6.941312000	8.414726000	-2.483324000
1	7.912788000	6.983797000	-2.062132000
1	12.642682000	11.426474000	3.572608000
1	12.251884000	12.730322000	4.723950000
1	11.775763000	12.878848000	3.004382000
1	8.640192000	11.647037000	2.373257000
1	8.559327000	13.575670000	0.843121000
1	8.121591000	10.988478000	-2.590655000
1	8.308773000	13.273105000	-1.600922000
1	6.421025000	10.777282000	5.282833000
1	6.050607000	13.100246000	6.084103000
1	7.840474000	14.800981000	5.969838000
1	10.098258000	14.245246000	5.073057000
1	5.532782000	6.143182000	4.231887000
1	4.277192000	4.655909000	5.768120000
1	5.290551000	3.822839000	7.867367000
1	7.619602000	4.441049000	8.508309000
8	6.531360000	8.161506000	3.085599000
58	4.622140000	8.914018000	2.423214000
8	4.154706000	6.411181000	2.344428000
7	4.884178000	6.169033000	1.326169000
8	5.250436000	5.038902000	1.022261000
8	5.266764000	7.201726000	0.650099000
8	5.302528000	9.731546000	-0.092439000
1	4.656211000	9.141261000	-0.527670000
1	4.867988000	10.606897000	-0.108186000
8	2.851116000	8.760934000	0.690265000
7	1.850729000	9.053324000	1.473243000
8	0.716772000	9.144118000	1.068218000
8	2.181518000	9.229159000	2.704585000
8	3.882095000	7.901885000	4.562182000

8	4.430366000	10.000879000	4.648262000
7	4.082293000	8.936048000	5.300071000
8	3.977917000	8.916924000	6.508785000
8	6.106703000	10.875270000	2.583701000
7	5.264862000	11.801384000	2.252697000
8	4.106807000	11.370062000	1.887771000
8	5.560509000	12.979984000	2.278507000
1	8.874375000	4.147341000	1.641121000

Table 15. [(MeTBEN)Mn^{IV}-O-$Ce^{IV}(NO_3)_4]^{2+}$ **(2M)**

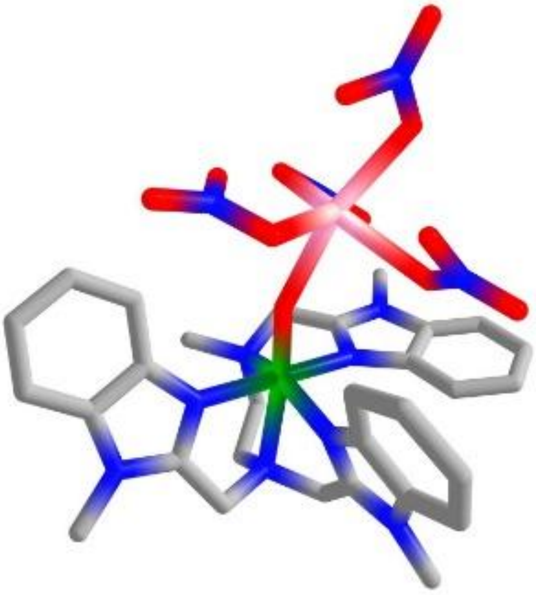

25	8.004793000	8.077100000	3.223147000
7	9.099691000	6.350981000	6.791098000
7	8.096980000	7.050849000	4.925789000
7	10.286447000	7.930291000	3.679207000
7	8.574236000	6.219748000	2.024497000
7	7.906917000	8.342984000	-0.880130000
7	8.496716000	9.816128000	3.968348000
7	8.181081000	8.746914000	1.300761000
7	9.933275000	11.512631000	4.104853000
6	7.801003000	5.821087000	6.809989000
6	5.886033000	5.834128000	5.294049000
6	9.232343000	7.067393000	5.653048000
6	5.238562000	5.011105000	6.212446000
6	7.175360000	6.257877000	5.618763000

6	10.828668000	6.715307000	3.015741000
6	10.086458000	6.162356000	7.851898000
6	5.857987000	4.606558000	7.412203000
6	9.764935000	10.229308000	3.721749000
6	7.815724000	10.874394000	4.567171000
6	8.228350000	4.922910000	2.649995000
6	10.415572000	7.825302000	5.160907000
6	7.157527000	4.997577000	7.733614000
6	10.046593000	6.347002000	1.769651000
6	8.712982000	11.968414000	4.629722000
6	7.853055000	6.376673000	0.734906000
6	8.048306000	9.725776000	-0.745039000
6	7.965114000	7.808008000	0.353581000
6	8.216855000	9.983591000	0.637369000
6	10.765023000	9.248453000	3.189436000
6	6.153774000	12.200522000	5.623978000
6	6.529404000	10.966977000	5.097906000
6	8.016277000	10.736757000	-1.705632000
6	8.339078000	13.205015000	5.156225000
6	8.162676000	12.041281000	-1.243414000
6	7.583871000	7.660396000	-2.130817000
6	8.310347000	12.315737000	0.129627000
6	7.034869000	13.301002000	5.640395000
6	8.334110000	11.303721000	1.084221000
6	11.131618000	12.335717000	3.959325000
1	11.041179000	6.609714000	7.559207000
1	10.231398000	5.089861000	8.031546000
1	9.735298000	6.641233000	8.775032000
1	11.895637000	6.837622000	2.767364000
1	10.763221000	5.900176000	3.748705000
1	8.777611000	4.800506000	3.588480000
1	7.155350000	4.901344000	2.867378000
1	10.417568000	8.838296000	5.591937000
1	11.368833000	7.349442000	5.440302000
1	10.186426000	7.111018000	0.994686000
1	10.429703000	5.395080000	1.365172000
1	8.271452000	5.705734000	-0.033937000
1	6.807686000	6.077171000	0.887235000
1	10.759294000	9.239315000	2.089132000
1	11.790154000	9.475064000	3.526544000
1	8.264007000	8.007876000	-2.916949000
1	6.543437000	7.874794000	-2.404840000
1	7.696698000	6.579880000	-2.005343000

1	11.958776000	11.725642000	3.584825000
1	11.404318000	12.762998000	4.932034000
1	10.935656000	13.146367000	3.245738000
1	8.340774000	11.558540000	2.137813000
1	8.363531000	13.352944000	0.458583000
1	7.867758000	10.521826000	-2.762979000
1	8.134638000	12.866391000	-1.954123000
1	5.853473000	10.114152000	5.126007000
1	5.149239000	12.314715000	6.029142000
1	6.691440000	14.249089000	6.052659000
1	9.022500000	14.052466000	5.193421000
1	5.402316000	6.143971000	4.371318000
1	4.222903000	4.680745000	5.999035000
1	5.309844000	3.967930000	8.104116000
1	7.635978000	4.672083000	8.656576000
1	8.480616000	4.092547000	1.971405000
8	6.308026000	8.111695000	3.156211000
58	4.177708000	8.961820000	2.565999000
8	3.377527000	7.210844000	4.095800000
7	3.454119000	7.921393000	5.175047000
8	3.154113000	7.503782000	6.264542000
8	3.906141000	9.126924000	4.983480000
8	2.696575000	9.761212000	0.875952000
8	4.045463000	11.341077000	3.209564000
7	5.018842000	11.729917000	2.460646000
8	5.417123000	12.869940000	2.422368000
8	5.566528000	10.777439000	1.761157000
8	3.985784000	6.839953000	1.360432000
7	4.678843000	7.175789000	0.323381000
8	5.123255000	8.384943000	0.325296000
8	4.931825000	6.389903000	-0.573213000
8	1.800172000	9.292420000	2.802446000
7	1.607386000	9.755940000	1.605347000
8	0.548096000	10.139079000	1.200627000

Table 16. [(MeTBEN)Mn^{IV}-O-Ce^{IV}(OH$_2$)(NO$_3$)$_4$]$^{2+}$

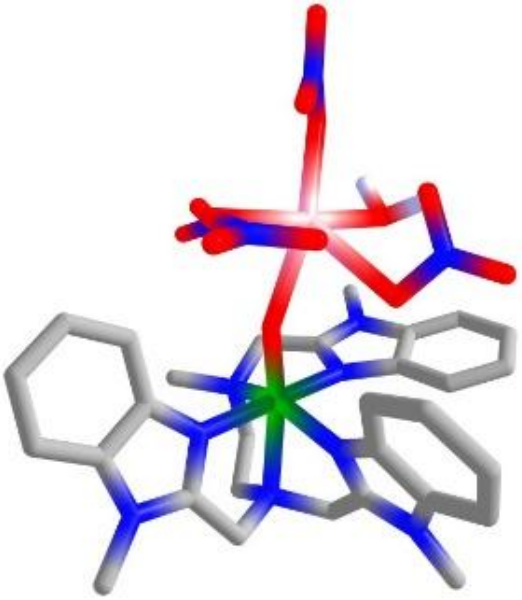

25	8.368158000	8.132784000	3.173724000
7	9.317623000	6.320405000	6.734255000
7	8.385069000	7.063431000	4.848910000
7	10.629316000	7.838880000	3.637065000
7	8.869013000	6.328705000	1.871974000
7	8.228507000	8.655887000	-0.903151000
7	8.963157000	9.821844000	4.000321000
7	8.572162000	8.908447000	1.288523000
7	10.528630000	11.395785000	4.215683000
6	7.980734000	5.895868000	6.753881000
6	6.071327000	6.052858000	5.239809000
6	9.511994000	7.006458000	5.587345000
6	5.353484000	5.317376000	6.179933000
6	7.397056000	6.359431000	5.550717000
6	11.115412000	6.617405000	2.948351000
6	10.282107000	6.072543000	7.802577000
6	5.936534000	4.879824000	7.385415000
6	10.253518000	10.157438000	3.752685000
6	8.380391000	10.886904000	4.687664000
6	8.414331000	5.035372000	2.434747000
6	10.746627000	7.690517000	5.114567000
6	7.268861000	5.153788000	7.695210000
6	10.352475000	6.365477000	1.666433000

6	9.360368000	11.901980000	4.804661000
6	8.228648000	6.579132000	0.556216000
6	8.377009000	10.026412000	-0.681437000
6	8.340244000	8.034776000	0.287539000
6	8.580642000	10.189739000	0.709779000
6	11.183339000	9.136654000	3.176476000
6	6.842085000	12.269099000	5.855472000
6	7.109002000	11.054095000	5.233075000
6	8.314817000	11.098225000	-1.571144000
6	9.096070000	13.125422000	5.422250000
6	8.457968000	12.371146000	-1.024962000
6	7.900269000	8.051347000	-2.190197000
6	8.637316000	12.552892000	0.359811000
6	7.812822000	13.288887000	5.941554000
6	8.698955000	11.477839000	1.243068000
6	11.783823000	12.133081000	4.096591000
1	11.268897000	6.442601000	7.508341000
1	10.345176000	4.994717000	7.997224000
1	9.963641000	6.588494000	8.717625000
1	12.196820000	6.677331000	2.741764000
1	10.967378000	5.779914000	3.643178000
1	8.931913000	4.838026000	3.378737000
1	7.337565000	5.082358000	2.625566000
1	10.816589000	8.689801000	5.571069000
1	11.664804000	7.144433000	5.383766000
1	10.565669000	7.158246000	0.937528000
1	10.681053000	5.412530000	1.219014000
1	8.712592000	5.971503000	-0.227466000
1	7.174003000	6.278444000	0.597147000
1	11.163819000	9.155716000	2.076752000
1	12.224644000	9.290284000	3.505682000
1	8.683212000	8.282776000	-2.922935000
1	6.939283000	8.447992000	-2.540912000
1	7.812026000	6.966899000	-2.079868000
1	12.556123000	11.487055000	3.668894000
1	12.103578000	12.477684000	5.087589000
1	11.639853000	12.999220000	3.438051000
1	8.749132000	11.659311000	2.310410000
1	8.697548000	13.565353000	0.757420000
1	8.154136000	10.952326000	-2.638694000
1	8.411926000	13.242179000	-1.677788000
1	6.348573000	10.284220000	5.152619000
1	5.852782000	12.435832000	6.280191000

1	7.555644000	14.227358000	6.431725000
1	9.846410000	13.910984000	5.502352000
1	5.609879000	6.380890000	4.314176000
1	4.310175000	5.081622000	5.974197000
1	5.334448000	4.309793000	8.092332000
1	7.718986000	4.805581000	8.624077000
8	6.677550000	8.183492000	3.069703000
58	4.563618000	8.986853000	2.323772000
8	4.195131000	6.490723000	2.340211000
7	4.888890000	6.244779000	1.298371000
8	5.237001000	5.121519000	0.972290000
8	5.262115000	7.289274000	0.623544000
8	5.268853000	9.786821000	-0.162959000
1	4.608699000	9.254658000	-0.650529000
1	4.955834000	10.705953000	-0.266613000
8	2.842758000	8.766862000	0.620524000
7	1.828046000	9.099533000	1.380681000
8	0.701665000	9.156386000	0.973615000
8	2.171191000	9.353179000	2.598716000
8	3.841826000	7.964554000	4.433389000
8	4.265387000	10.089773000	4.509376000
7	3.914261000	9.025949000	5.165269000
8	3.703735000	9.027358000	6.353473000
8	6.154182000	10.818905000	2.509953000
7	5.347568000	11.804500000	2.235241000
8	4.165160000	11.434244000	1.890577000
8	5.702465000	12.959403000	2.298927000
1	8.619666000	4.215885000	1.728110000

Table 17. [(MeTBEN)Mn^{IV}(O)]$^{2+}$ **(3M)**

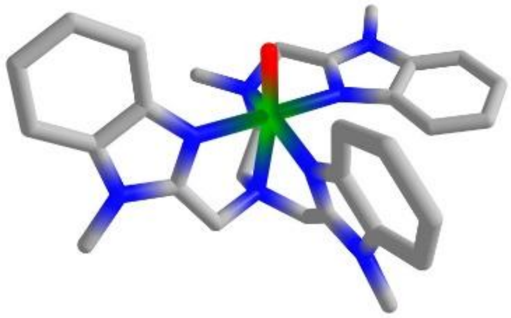

25	8.339227000	7.975263000	3.076232000
7	9.182351000	6.124256000	6.647508000

7	8.292332000	6.967491000	4.784226000
7	10.576088000	7.861256000	3.689958000
7	9.010177000	6.220210000	1.845414000
7	8.307166000	8.413539000	-1.029802000
7	8.692335000	9.720175000	3.855985000
7	8.589302000	8.762181000	1.160555000
7	10.059723000	11.414318000	4.314861000
6	7.848912000	5.692743000	6.609613000
6	5.958529000	5.979556000	5.084466000
6	9.395808000	6.880887000	5.544415000
6	5.223680000	5.182922000	5.959690000
6	7.290366000	6.231543000	5.426556000
6	11.198904000	6.723703000	2.961257000
6	10.120486000	5.828638000	7.726195000
6	5.788579000	4.650478000	7.137231000
6	9.971272000	10.147231000	3.841083000
6	7.902176000	10.753785000	4.346143000
6	8.671050000	4.903307000	2.425242000
6	10.619703000	7.638300000	5.160883000
6	7.115287000	4.895001000	7.488200000
6	10.482420000	6.400574000	1.658510000
6	8.761600000	11.838958000	4.640634000
6	8.313115000	6.404632000	0.548380000
6	8.401706000	9.796555000	-0.857547000
6	8.411012000	7.848227000	0.196830000
6	8.568207000	10.014147000	0.533648000
6	11.049914000	9.225271000	3.352492000
6	6.038881000	12.049278000	5.033159000
6	6.521809000	10.842579000	4.534439000
6	8.345540000	10.835289000	-1.787214000
6	8.277500000	13.049639000	5.139719000
6	8.453278000	12.129053000	-1.283709000
6	8.083770000	7.747818000	-2.310039000
6	8.599440000	12.364943000	0.097098000
6	6.898802000	13.128729000	5.331475000
6	8.657814000	11.323997000	1.020889000
6	11.255982000	12.244428000	4.428517000
8	6.721692000	7.816635000	2.797916000
1	11.118163000	6.192175000	7.461872000
1	10.166236000	4.744232000	7.885653000
1	9.793653000	6.317681000	8.653194000
1	12.267358000	6.908712000	2.762151000
1	11.148232000	5.855999000	3.632552000

1	9.201024000	4.765084000	3.373343000
1	7.594055000	4.869828000	2.624325000
1	10.609176000	8.617474000	5.665905000
1	11.549986000	7.130692000	5.461599000
1	10.629406000	7.214975000	0.938318000
1	10.922754000	5.488708000	1.219668000
1	8.737445000	5.747763000	-0.229987000
1	7.256619000	6.131769000	0.696316000
1	11.146164000	9.302050000	2.258312000
1	12.033129000	9.455465000	3.796673000
1	8.865779000	8.041591000	-3.021258000
1	7.101417000	8.030092000	-2.710093000
1	8.113220000	6.662722000	-2.174335000
1	12.146142000	11.643249000	4.220808000
1	11.324578000	12.650017000	5.445282000
1	11.206716000	13.071688000	3.708778000
1	8.724073000	11.541441000	2.081965000
1	8.652021000	13.393427000	0.453657000
1	8.215028000	10.650684000	-2.852926000
1	8.409771000	12.974581000	-1.969248000
1	5.872658000	10.004079000	4.283092000
1	4.967981000	12.169560000	5.194349000
1	6.472050000	14.053316000	5.719187000
1	8.930719000	13.891384000	5.366408000
1	5.533098000	6.409314000	4.179698000
1	4.179662000	4.968083000	5.732820000
1	5.171977000	4.035167000	7.791818000
1	7.544913000	4.485147000	8.401490000
1	8.946651000	4.087641000	1.737244000

Table 18. $[(MeTBEN)Mn^{IV}(OH)]^{3+}$

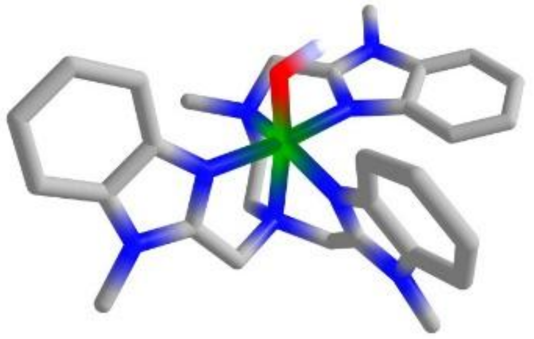

25	8.443340000	7.961381000	3.024667000

7	9.152865000	6.192700000	6.621768000
7	8.280960000	6.968468000	4.718912000
7	10.519766000	7.878746000	3.626645000
7	8.980958000	6.239388000	1.884489000
7	8.334289000	8.387719000	-1.018803000
7	8.708098000	9.713163000	3.823872000
7	8.580747000	8.739745000	1.177866000
7	10.067693000	11.422929000	4.259319000
6	7.826107000	5.734888000	6.567264000
6	5.954604000	5.923832000	5.006747000
6	9.375724000	6.918703000	5.511169000
6	5.226156000	5.134276000	5.896678000
6	7.275762000	6.228304000	5.358254000
6	11.183352000	6.718207000	2.947010000
6	10.076555000	5.939194000	7.730421000
6	5.783604000	4.654495000	7.097597000
6	9.984282000	10.169010000	3.771779000
6	7.917159000	10.720400000	4.370612000
6	8.619102000	4.927702000	2.488498000
6	10.578579000	7.694296000	5.117486000
6	7.100575000	4.946934000	7.458714000
6	10.467231000	6.359535000	1.662962000
6	8.773277000	11.817327000	4.643655000
6	8.296366000	6.386014000	0.566434000
6	8.429777000	9.775623000	-0.843427000
6	8.413680000	7.819755000	0.195692000
6	8.566713000	10.001396000	0.549588000
6	11.043623000	9.237088000	3.273385000
6	6.076096000	11.970918000	5.204637000
6	6.549906000	10.781538000	4.653879000
6	8.388197000	10.805712000	-1.781664000
6	8.299456000	13.008220000	5.194489000
6	8.475817000	12.104849000	-1.284013000
6	8.143245000	7.725065000	-2.313000000
6	8.584416000	12.349388000	0.097545000
6	6.931505000	13.061467000	5.467775000
6	8.629564000	11.314587000	1.031879000
6	11.259474000	12.269408000	4.362430000
8	6.658142000	7.902670000	2.851003000
1	11.070428000	6.324212000	7.483367000
1	10.142141000	4.858945000	7.908351000
1	9.710250000	6.437316000	8.637272000
1	12.245366000	6.932212000	2.753254000

1	11.146540000	5.874587000	3.647836000
1	9.176283000	4.776772000	3.417457000
1	7.547258000	4.916510000	2.712129000
1	10.552848000	8.685014000	5.597691000
1	11.525500000	7.214729000	5.406344000
1	10.632385000	7.131552000	0.901136000
1	10.857917000	5.409629000	1.264805000
1	8.742677000	5.708781000	-0.179104000
1	7.240760000	6.101020000	0.695485000
1	11.152660000	9.300181000	2.180130000
1	12.029435000	9.421930000	3.727808000
1	8.968294000	7.992596000	-2.984515000
1	7.191886000	8.050595000	-2.752251000
1	8.122498000	6.639978000	-2.177346000
1	12.139514000	11.717260000	4.019975000
1	11.401967000	12.575531000	5.406118000
1	11.130328000	13.160128000	3.734871000
1	8.662346000	11.545622000	2.091254000
1	8.617545000	13.380492000	0.448682000
1	8.283859000	10.613789000	-2.849083000
1	8.443023000	12.945543000	-1.976312000
1	5.894451000	9.933732000	4.459583000
1	5.016631000	12.063266000	5.442416000
1	6.514079000	13.970275000	5.900741000
1	8.952123000	13.854012000	5.408578000
1	5.512409000	6.300628000	4.088574000
1	4.192768000	4.883761000	5.657948000
1	5.174379000	4.042556000	7.762307000
1	7.526394000	4.577449000	8.391044000
1	8.859559000	4.114204000	1.787479000
1	6.288613000	8.479103000	2.154100000

84

Mn_IV_BnTBEN_Oxo_G2_Quartet.log

84

Final Energy = -3059.2988489200

C	-1.4767740	0.3339030	2.2715590
H	-2.3797790	-0.2690040	2.1443740
H	-1.4486330	0.6446670	3.3174530
N	-0.3095300	-0.4920940	1.8995610
C	-0.4650460	-1.9344810	2.1857250
H	-0.3035590	-2.1517290	3.2443360
H	-1.4899310	-2.2204140	1.9582060
C	0.5383170	-2.6891210	1.3529380

H	1.5373040	-2.3244420	1.5835420
H	0.5246050	-3.7545290	1.5964740
N	0.3373050	-2.4973180	-0.1118350
C	0.9527910	0.0385990	2.4814310
H	1.4121700	-0.7119680	3.1286580
H	0.7324310	0.8884180	3.1316940
C	-0.8263510	-3.3028210	-0.5754600
H	-0.8665850	-4.2518260	-0.0353090
H	-0.6677060	-3.5501500	-1.6275670
C	1.5175340	-3.0483080	-0.8859760
H	1.2708690	-2.8719470	-1.9313170
H	1.5060280	-4.1313200	-0.7209310
C	2.8874590	-2.5130590	-0.6069270
C	3.6296020	-2.8925540	0.5147860
C	3.5056640	-1.7191830	-1.5693520
C	4.9238650	-2.4327850	0.6966210
H	3.2070000	-3.5777110	1.2393660
C	4.8105350	-1.2808810	-1.4040810
H	2.9505380	-1.4425860	-2.4581210
C	5.5165670	-1.6209550	-0.2612850
H	5.4793400	-2.7304510	1.5763810
H	5.2687420	-0.6618250	-2.1644550
H	6.5314830	-1.2703950	-0.1241370
Mn	-0.2188650	-0.3674430	-0.3584540
O	-0.0054300	-0.2884230	-2.0015720
C	-2.0839670	-2.5423390	-0.4748070
N	-2.0804500	-1.2188710	-0.4423890
N	-3.3246650	-3.0543010	-0.5020920
C	-3.4127470	-0.8239450	-0.4829520
C	-4.2013980	-1.9844700	-0.5078150
C	-3.7120030	-4.4493410	-0.5494030
C	-4.0243740	0.4258060	-0.5276820
C	-5.5859600	-1.9533300	-0.5468250
H	-4.3764850	-4.6740750	0.2816050
H	-4.2240580	-4.6621040	-1.4853020
H	-2.8327220	-5.0787740	-0.4779130
C	-5.4067630	0.4623360	-0.5661160
H	-3.4558540	1.3417820	-0.5714820
C	-6.1772620	-0.7039910	-0.5683280
H	-6.1728530	-2.8611680	-0.5679750
H	-5.9033560	1.4222710	-0.6082310
H	-7.2554120	-0.6276950	-0.6012210
C	-1.5213700	1.4877160	1.3351870

N	-1.0286380	1.3653190	0.1052960
N	-2.0429110	2.7080750	1.5358040
C	-1.2241840	2.5691620	-0.5379340
C	-1.8739350	3.4263270	0.3598840
C	-2.6975990	3.2076330	2.7285330
C	-0.9041200	2.9807560	-1.8241750
C	-2.2259460	4.7223210	0.0144340
H	-2.2138960	4.1251120	3.0544320
H	-3.7457980	3.4090250	2.5178490
H	-2.6318480	2.4706570	3.5204390
C	-1.2521710	4.2731470	-2.1694780
H	-0.4112800	2.3043260	-2.5094490
C	-1.8994360	5.1271720	-1.2673200
H	-2.7287500	5.3815870	0.7081010
H	-1.0225190	4.6360070	-3.1620770
H	-2.1533250	6.1301530	-1.5820970
C	1.8775960	0.4722750	1.4074260
N	1.5393050	0.4046160	0.1260840
N	3.0656000	1.0703330	1.5796740
C	2.5308500	1.0598600	-0.5854270
C	3.5126010	1.4620840	0.3266930
C	3.7789530	1.2758120	2.8228420
C	2.6573710	1.3452460	-1.9383710
C	4.6642030	2.1247360	-0.0659660
H	4.7081010	0.7088140	2.8121800
H	4.0051230	2.3320320	2.9485050
H	3.1718800	0.9434160	3.6572890
C	3.7968360	2.0220370	-2.3335660
H	1.8916140	1.0327420	-2.6343570
C	4.7859700	2.3980560	-1.4170880
H	5.4234100	2.4201400	0.6450330
H	3.9309600	2.2648700	-3.3791290
H	5.6638690	2.9207330	-1.7720050

84
Mn_IV_BnTBEN_Oxo_G3_Doublet.log
84
Final Energy = -3059.2778836000

C	1.6269830	0.3631600	2.2240030
H	1.7511130	1.4433160	2.1164880
H	1.8952040	0.1130740	3.2526220
N	0.2266240	0.0129050	1.9054060
C	-0.7624440	1.0200380	2.3375740
H	-0.9748840	0.9460360	3.4064560

H	-0.3397310	2.0090390	2.1651150
C	-2.0197030	0.8081580	1.5396940
H	-2.4011760	-0.2005600	1.7024190
H	-2.8060090	1.4981050	1.8489720
N	-1.7650030	0.9435340	0.0721660
C	-0.1032380	-1.3469110	2.4163060
H	-0.8265800	-1.2730680	3.2324520
H	0.7906310	-1.7960520	2.8545740
C	-1.6704960	2.3793490	-0.3212620
H	-2.3337250	2.9930300	0.2907110
H	-2.0215640	2.4689400	-1.3513900
C	-2.8811900	0.2769130	-0.7073540
H	-2.8551430	-0.7733090	-0.4251860
H	-2.5925090	0.3466100	-1.7551240
C	-4.2430250	0.8583350	-0.4716900
C	-4.7317670	1.8825260	-1.2813090
C	-5.0549350	0.3697960	0.5514470
C	-5.9890510	2.4215680	-1.0590730
H	-4.1319190	2.2483160	-2.1065560
C	-6.3120030	0.9071610	0.7755710
H	-4.7049120	-0.4497930	1.1683780
C	-6.7790740	1.9387230	-0.0261810
H	-6.3553830	3.2126060	-1.7000280
H	-6.9310460	0.5139500	1.5711780
H	-7.7624210	2.3566220	0.1453220
Mn	0.0878740	0.0220500	-0.3762330
O	-0.0493920	-0.1019970	-1.9593760
C	-0.2654840	2.8283990	-0.2748520
N	0.7210230	1.9491610	-0.3623580
N	0.1730250	4.0970710	-0.2398520
C	1.8950840	2.6871170	-0.4119720
C	1.5542220	4.0453500	-0.3222050
C	-0.6143370	5.3105610	-0.1630380
C	3.2285760	2.3133070	-0.5502010
C	2.4995820	5.0582440	-0.3382690
H	-0.3355490	5.8771040	0.7225050
H	-0.4421460	5.9206540	-1.0469650
H	-1.6682540	5.0631290	-0.1081800
C	4.1763630	3.3208780	-0.5656360
H	3.5219470	1.2816280	-0.6771750
C	3.8214820	4.6690690	-0.4550410
H	2.2178250	6.0998590	-0.2701110
H	5.2196660	3.0584310	-0.6776000

H	4.5958550	5.4236730	-0.4734140
C	2.4525730	-0.3477950	1.2181020
N	1.9211800	-0.6212290	0.0353700
N	3.7226410	-0.7836180	1.3080620
C	2.8830570	-1.2795490	-0.7004200
C	4.0337630	-1.3774910	0.0958250
C	4.6255110	-0.6481720	2.4322540
C	2.8543590	-1.7942100	-1.9897170
C	5.1941440	-1.9825630	-0.3639410
H	5.0527220	-1.6174380	2.6772690
H	5.4272310	0.0461670	2.1877130
H	4.0841040	-0.2738630	3.2935740
C	4.0090770	-2.3998520	-2.4475550
H	1.9604440	-1.7184220	-2.5952900
C	5.1572170	-2.4908980	-1.6496770
H	6.0819660	-2.0519170	0.2491440
H	4.0299590	-2.8143920	-3.4462160
H	6.0392630	-2.9715760	-2.0503730
C	-0.6078660	-2.2148850	1.3255860
N	-0.6289200	-1.8121830	0.0678050
N	-1.0524860	-3.4800600	1.4648410
C	-1.1059700	-2.8753170	-0.6789970
C	-1.3803740	-3.9329720	0.1992240
C	-1.1744180	-4.2467820	2.6871660
C	-1.3291380	-3.0095910	-2.0444360
C	-1.8807460	-5.1491590	-0.2395490
H	-2.2170390	-4.4999680	2.8686550
H	-0.5938900	-5.1632810	2.6078240
H	-0.8017250	-3.6695420	3.5259520
C	-1.8260700	-4.2226040	-2.4856030
H	-1.1148440	-2.1942640	-2.7210080
C	-2.0972040	-5.2728330	-1.6005480
H	-2.0909320	-5.9597670	0.4444770
H	-2.0102140	-4.3655090	-3.5418870
H	-2.4853950	-6.2043020	-1.9898930

63
Mn_IV_BnTPEN_G1_Quartet.log
63
Final Energy = -2546.4560247400

C	-3.2548250	0.3508710	0.0982100
C	-2.5216380	-0.2708170	2.2408170
C	-3.8259840	-0.2985110	2.7170510
C	-4.8687370	0.0087870	1.8417890

C	-4.5792450	0.3353470	0.5151080
H	-1.6598340	-0.4939290	2.8582160
H	-4.0136320	-0.5587230	3.7523410
H	-5.8979580	-0.0095910	2.1848530
H	-5.3677290	0.5727520	-0.1902430
C	-2.8152500	0.7360900	-1.2927750
H	-2.5943730	1.8064060	-1.3270650
H	-3.5999760	0.5438060	-2.0309960
N	-1.5714100	-0.0105710	-1.6041200
C	-0.7103450	0.6363170	-2.6267530
H	-1.0938970	0.4630290	-3.6384500
H	-0.7177750	1.7120110	-2.4523890
C	0.6865510	0.0565120	-2.4987170
H	0.6641660	-1.0133040	-2.7181040
H	1.3661380	0.5216200	-3.2193070
N	1.2581300	0.2046300	-1.1078030
C	-1.8947790	-1.4260100	-1.9597280
H	-1.7113510	-1.5981080	-3.0244810
H	-2.9648500	-1.5917110	-1.8023810
C	-1.1505720	-2.4263680	-1.1183720
C	-1.1825130	-3.7900730	-1.4016500
C	-0.5624290	-4.6791170	-0.5276700
H	-1.6967160	-4.1422860	-2.2894430
C	0.0752500	-2.8166870	0.8356200
C	0.0655150	-4.1844820	0.6192220
H	-0.5765090	-5.7445890	-0.7325320
H	0.5419950	-2.3578190	1.6972420
H	0.5450170	-4.8442090	1.3330020
C	1.8079660	1.5760780	-0.8991440
H	2.3671190	1.9039120	-1.7808140
H	2.5124320	1.5183170	-0.0659670
C	2.3812370	-0.8360020	-1.0131850
H	2.8190350	-0.8983500	-2.0136160
H	1.8932500	-1.7878900	-0.8089160
C	0.7565080	2.5662160	-0.5080890
C	0.9176440	3.9358340	-0.7005970
C	-1.2117010	2.9149960	0.6930220
C	-0.0245100	4.8110880	-0.1658460
H	1.7764350	4.3010190	-1.2528020
C	-1.0995770	4.2906590	0.5573590
H	-2.0313950	2.4715260	1.2411530
H	0.0818980	5.8819580	-0.3038150
H	-1.8476140	4.9336000	1.0063860

C	3.4921810	-0.5645140	-0.0317570
C	4.7061540	-0.0465690	-0.5101700
C	3.3741480	-0.8589850	1.3333390
C	5.7751650	0.1867670	0.3569550
H	4.8139990	0.1764740	-1.5689050
C	4.4417860	-0.6268740	2.2026960
H	2.4391760	-1.2420700	1.7202270
C	5.6438270	-0.1022020	1.7183100
H	6.7071260	0.5886230	-0.0301730
H	4.3343200	-0.8582080	3.2586900
H	6.4739050	0.0757580	2.3962020
N	-2.2511440	0.0491010	0.9612030
N	-0.5058460	-1.9568670	-0.0302110
N	-0.3121490	2.0678570	0.1513300
Mn	-0.3899080	0.0334170	0.2058380
O	0.4164090	-0.0643230	1.6516340

63

Mn_IV_BnTPEN_Oxo_G1_Doublet.log

63

Final Energy = -2546.4399665600

C	3.1565640	0.4743990	-0.1783280
C	2.4082740	-0.2990230	-2.2418660
C	3.6805110	-0.2298420	-2.7676450
C	4.7190800	0.2082210	-1.9625740
C	4.4527090	0.5564580	-0.6501270
H	1.5618660	-0.6348690	-2.8244850
H	3.8447440	-0.5214580	-3.7943140
H	5.7270250	0.2675700	-2.3489080
H	5.2373470	0.8862460	0.0158090
C	2.7468410	0.8464540	1.2084010
H	2.4603360	1.8996950	1.2458750
H	3.5660170	0.7184910	1.9190640
N	1.5694560	0.0322020	1.5654480
C	0.7489560	0.6191890	2.6444340
H	1.1993030	0.4536680	3.6254800
H	0.7009760	1.6971200	2.4954020
C	-0.6079590	-0.0143630	2.5702500
H	-0.5325670	-1.0940950	2.7090290
H	-1.2727430	0.3593610	3.3506670
N	-1.2380630	0.2124670	1.2298670
C	1.9773400	-1.3627160	1.8882180
H	1.8842360	-1.5399140	2.9612400
H	3.0405740	-1.4724970	1.6672280

C	1.2401930	-2.4038390	1.1106730
C	1.3744860	-3.7509000	1.4095350
C	0.7711220	-4.6888710	0.5954010
H	1.9603220	-4.0475670	2.2684320
C	-0.0476490	-2.9043620	-0.7461440
C	0.0622700	-4.2569070	-0.5152550
H	0.8641770	-5.7434450	0.8151740
H	-0.5771300	-2.5050430	-1.6000230
H	-0.4035200	-4.9537480	-1.1961230
C	-1.7580990	1.6037130	1.1309640
H	-2.1679530	1.9272410	2.0902050
H	-2.5892630	1.5917780	0.4246280
C	-2.3849020	-0.7951940	1.1447100
H	-2.8712790	-0.7677100	2.1228540
H	-1.9094980	-1.7699910	1.0486120
C	-0.7669480	2.5738700	0.5967740
C	-0.8948230	3.9392080	0.7952700
C	1.0231690	2.9009920	-0.8330140
C	-0.0281970	4.7964070	0.1453920
H	-1.6744230	4.3122080	1.4442170
C	0.9376200	4.2686450	-0.6977630
H	1.7654520	2.4497250	-1.4760020
H	-0.1100120	5.8653020	0.2869590
H	1.6227870	4.9022090	-1.2407490
C	-3.4136080	-0.5829780	0.0820980
C	-4.5453800	0.1894940	0.3544460
C	-3.3150980	-1.2004670	-1.1623530
C	-5.5300870	0.3682110	-0.6018860
H	-4.6551200	0.6464050	1.3314360
C	-4.2996200	-1.0237790	-2.1219880
H	-2.4702280	-1.8387340	-1.3747600
C	-5.4051480	-0.2341160	-1.8464190
H	-6.3996360	0.9697990	-0.3727250
H	-4.2067740	-1.5137060	-3.0822000
H	-6.1758860	-0.0990520	-2.5939510
N	2.1496750	0.0567130	-0.9729300
N	0.5063670	-1.9893090	0.0666090
N	0.1995490	2.0630110	-0.1833580
Mn	0.2814800	0.0174050	-0.2202300
O	-0.5718370	-0.1366380	-1.5573780

63

Mn_IV_BnTPEN_Oxo_G1_Sextet.log

63

Final Energy = -2546.4156554700

C	2.7906210	1.1844890	-0.1433280
C	2.4836180	0.1502620	-2.1846520
C	3.8300240	0.2443650	-2.4849940
C	4.6750880	0.8308520	-1.5578010
C	4.1503030	1.3071140	-0.3666930
H	1.7804880	-0.3114780	-2.8685480
H	4.2029250	-0.1405650	-3.4228720
H	5.7352690	0.9120560	-1.7558550
H	4.7827210	1.7653630	0.3809460
C	2.1108290	1.7182740	1.0791460
H	1.6091650	2.6549440	0.8252390
H	2.8279050	1.9462670	1.8698900
N	1.0675190	0.7850210	1.5836410
C	0.1148260	1.5023050	2.4770390
H	0.5900140	1.7271620	3.4349260
H	-0.1121180	2.4580740	2.0077270
C	-1.1363460	0.6896220	2.6989010
H	-0.9070480	-0.2120060	3.2655930
H	-1.8416540	1.2612840	3.3082550
N	-1.7638210	0.2592230	1.4241620
C	1.6999240	-0.3672760	2.2915340
H	1.0077540	-0.7171980	3.0583520
H	2.5971150	-0.0359730	2.8171520
C	1.9936380	-1.5094110	1.3763070
C	3.0475650	-2.3824430	1.5809730
C	3.2145480	-3.4484150	0.7136210
H	3.7256560	-2.2196790	2.4066480
C	1.3103880	-2.6967010	-0.4950030
C	2.3308540	-3.6113610	-0.3415820
H	4.0341300	-4.1398710	0.8541640
H	0.6088290	-2.7417270	-1.3196240
H	2.4345020	-4.4237180	-1.0453880
C	-2.6310490	1.3101050	0.8648460
H	-3.2472850	1.7620300	1.6477340
H	-3.3196170	0.8299900	0.1654460
C	-2.5707740	-0.9758970	1.6635830
H	-3.4179070	-0.7339890	2.3139010
H	-1.9260020	-1.6656070	2.2087090
C	-1.9081110	2.3644220	0.0924170
C	-2.4352830	3.6386250	-0.0447500
C	-0.1624440	2.8886950	-1.3297270
C	-1.7957890	4.5507000	-0.8625180

H	-3.3404390	3.8981340	0.4865420
C	-0.6416340	4.1661780	-1.5259930
H	0.7404880	2.5463100	-1.8165230
H	-2.1928980	5.5496550	-0.9810640
H	-0.1117530	4.8426580	-2.1799690
C	-3.0407930	-1.6167000	0.3969040
C	-4.3273390	-1.4422430	-0.0801980
C	-2.1534820	-2.4223010	-0.3412670
C	-4.7267470	-2.0391790	-1.2741170
H	-5.0330260	-0.8444850	0.4837980
C	-2.5624230	-3.0310680	-1.5355430
H	-1.2071600	-2.6972620	0.1034450
C	-3.8422950	-2.8290160	-2.0042080
H	-5.7364440	-1.8887870	-1.6327760
H	-1.8680630	-3.6592160	-2.0768420
H	-4.1658000	-3.2922070	-2.9261130
N	1.9735530	0.6143650	-1.0424350
N	1.1386170	-1.6750660	0.3528090
N	-0.7759090	2.0039540	-0.5299230
Mn	-0.0333190	0.0278290	-0.1056600
O	-0.9753470	-0.7602950	-1.4333200

63
Mn_IV_BnTPEN_Oxo_G2_Doublet.log
63
Final Energy = -2546.4382702700

C	-2.2525600	1.9064570	0.5534750
C	-1.4784120	2.3657430	-1.5941760
C	-2.2636030	3.4960960	-1.6723550
C	-3.0678370	3.8327540	-0.5958550
C	-3.0529360	3.0326000	0.5329680
H	-0.8334920	2.0567920	-2.4050940
H	-2.2349240	4.0994050	-2.5673910
H	-3.6904540	4.7158700	-0.6297520
H	-3.6490570	3.2730910	1.4018050
C	-2.1638870	0.9659660	1.7102180
H	-2.8960990	0.1625140	1.6034880
H	-2.3846230	1.4702790	2.6531440
N	-0.8130470	0.3731390	1.6999080
C	-0.7026850	-0.8881760	2.4601420
H	-0.6035520	-0.7032580	3.5312510
H	-1.6208740	-1.4587980	2.3199000
C	0.4908250	-1.6199820	1.9165880
H	1.3802180	-0.9948580	1.9863470

H	0.6924150	-2.5433770	2.4637800
N	0.2619390	-1.9251870	0.4752150
C	0.1958190	1.3644490	2.1670610
H	0.5988820	1.0528860	3.1325590
H	-0.3078400	2.3134270	2.3593800
C	1.3000150	1.6114280	1.1929820
C	2.3540050	2.4525590	1.5142730
C	3.3006730	2.7472910	0.5535730
H	2.4085520	2.8773460	2.5071110
C	2.1014150	1.3577850	-0.9608840
C	3.1603970	2.2022840	-0.7131530
H	4.1290440	3.4028550	0.7847270
H	1.9425140	0.8993080	-1.9263970
H	3.8646040	2.4169740	-1.5035890
C	-0.8160210	-2.9653870	0.3655000
H	-0.8713290	-3.5393450	1.2919220
H	-0.5179580	-3.6765550	-0.4051140
C	1.4954950	-2.5668370	-0.1372000
H	1.1718450	-2.9191760	-1.1134130
H	1.6982370	-3.4493110	0.4789930
C	-2.1465240	-2.4314140	-0.0227410
C	-3.2872550	-3.2159300	0.0524750
C	-3.3262650	-0.7305870	-1.0530220
C	-4.4749490	-2.7239400	-0.4500350
H	-3.2261460	-4.2025070	0.4897680
C	-4.4897050	-1.4651590	-1.0324910
H	-3.2972170	0.2542630	-1.4960120
H	-5.3766200	-3.3186890	-0.4009940
H	-5.3894980	-1.0505360	-1.4617120
C	2.7467050	-1.7693780	-0.3190330
C	3.5910570	-1.4178340	0.7353850
C	3.1672970	-1.5068170	-1.6230240
C	4.7978300	-0.7820310	0.4899830
H	3.3308190	-1.6730910	1.7548810
C	4.3865020	-0.8967750	-1.8710420
H	2.5294800	-1.7959750	-2.4499860
C	5.2006230	-0.5237120	-0.8129970
H	5.4379350	-0.5117590	1.3196130
H	4.6976190	-0.7109730	-2.8907330
H	6.1516670	-0.0426530	-1.0010160
N	-1.4836590	1.5791780	-0.5056760
N	1.1982690	1.0451180	-0.0181080
N	-2.1749560	-1.1936590	-0.5362620

Mn	-0.4262340	-0.1339270	-0.4199960
O	0.0007180	-0.3993500	-1.9315960

63

Mn_IV_BnTPEN_Oxo_G2_Quartet.log

63

Final Energy = -2546.4592051400

C	-1.8880740	1.9998530	0.9495020
C	-1.8028240	2.4873490	-1.3341470
C	-2.3999640	3.7135000	-1.1317430
C	-2.7381170	4.0851250	0.1596510
C	-2.4777840	3.2220160	1.2112170
H	-1.5003070	2.1286560	-2.3095550
H	-2.5873690	4.3622240	-1.9741730
H	-3.1986960	5.0449260	0.3492600
H	-2.7279060	3.4857840	2.2289370
C	-1.6461850	0.9577080	1.9985130
H	-2.5244480	0.3100040	2.0553750
H	-1.5341810	1.4111710	2.9847120
N	-0.4845620	0.1135890	1.6476560
C	-0.5901360	-1.2799600	2.1454020
H	-0.3634470	-1.3415930	3.2125010
H	-1.6221990	-1.6018240	2.0248390
C	0.3746030	-2.1502760	1.3768740
H	1.3902680	-1.8595490	1.6284120
H	0.2656290	-3.1974380	1.6653470
N	0.2461830	-2.0132170	-0.1100030
C	0.7984860	0.7516940	2.0445840
H	1.4639050	-0.0064570	2.4603230
H	0.6287230	1.4623860	2.8548820
C	1.4842950	1.4281260	0.9023390
C	2.5018650	2.3483800	1.0942180
C	3.0680340	2.9665760	-0.0046400
H	2.8231150	2.5834130	2.0993400
C	1.6072040	1.7169730	-1.4035940
C	2.6033610	2.6571210	-1.2746390
H	3.8558810	3.6953410	0.1289740
H	1.1969980	1.4105780	-2.3574910
H	3.0080490	3.1313820	-2.1563180
C	-0.7954460	-2.9324760	-0.6487900
H	-0.6421610	-3.9392210	-0.2554520
H	-0.6421330	-2.9799020	-1.7293920
C	1.5235660	-2.4501160	-0.8087230
H	1.3220200	-2.3001180	-1.8681530

H	1.6021560	-3.5272190	-0.6322970
C	-2.1795240	-2.4553390	-0.4126830
C	-3.2564260	-3.3158440	-0.3001700
C	-3.5784210	-0.6124850	-0.3140120
C	-4.5310480	-2.7907410	-0.1984050
H	-3.0844860	-4.3825410	-0.2982260
C	-4.6940870	-1.4157390	-0.2154930
H	-3.6723790	0.4632270	-0.3289300
H	-5.3866390	-3.4456920	-0.1091710
H	-5.6702500	-0.9594590	-0.1486070
C	2.8080460	-1.7820670	-0.4352940
C	3.5465780	-2.1774890	0.6823320
C	3.3535460	-0.8213450	-1.2827700
C	4.7595810	-1.5772390	0.9787150
H	3.1818570	-2.9790880	1.3135400
C	4.5708260	-0.2251640	-0.9947260
H	2.8170440	-0.5527850	-2.1853530
C	5.2695500	-0.5915660	0.1458730
H	5.3153270	-1.8929700	1.8516630
H	4.9761670	0.5210540	-1.6659470
H	6.2200540	-0.1269080	0.3732070
N	-1.5618460	1.6553250	-0.3132610
N	1.0746500	1.1045360	-0.3340080
N	-2.3396990	-1.1203040	-0.4002480
Mn	-0.5595410	-0.0839830	-0.5135370
O	-0.4517710	-0.1920930	-2.1726360

63

Mn_IV_BnTPEN_Oxo_G2_Sextet.log

63

Final Energy = -2546.4114470400

C	-1.8140470	2.1493120	0.9692810
C	-1.6895750	2.7560100	-1.2530450
C	-2.0399450	4.0612180	-0.9599830
C	-2.2756280	4.4036720	0.3611740
C	-2.1592100	3.4343950	1.3452320
H	-1.4826320	2.4367220	-2.2684380
H	-2.1189090	4.7903900	-1.7529780
H	-2.5410560	5.4182000	0.6258550
H	-2.3293040	3.6673280	2.3871740
C	-1.6951520	1.0097320	1.9291460
H	-2.5707970	0.3634360	1.8352200
H	-1.6560740	1.3514090	2.9645900
N	-0.4988000	0.1753250	1.6229410

C	-0.5967190	-1.1360160	2.3271020
H	-0.4412490	-0.9970350	3.3990690
H	-1.6194190	-1.4923020	2.1998520
C	0.3974680	-2.1184760	1.7697620
H	1.4103610	-1.7480180	1.9154260
H	0.3304360	-3.0632420	2.3184030
N	0.2057480	-2.3334010	0.3194150
C	0.7553310	0.9049820	1.9968420
H	1.4682780	0.1819770	2.3952510
H	0.5449120	1.6003610	2.8104490
C	1.3972620	1.6054720	0.8437900
C	2.2512180	2.6810460	1.0130040
C	2.8512110	3.2464020	-0.0984080
H	2.4335950	3.0673150	2.0056840
C	1.7230290	1.6593340	-1.4602630
C	2.5863420	2.7273900	-1.3562740
H	3.5158710	4.0916680	0.0160900
H	1.4659820	1.2201000	-2.4168010
H	3.0331990	3.1438050	-2.2465440
C	-0.9458210	-3.2212430	0.0677970
H	-1.0682150	-3.9462960	0.8773810
H	-0.7243490	-3.8111680	-0.8243030
C	1.4225110	-2.9815060	-0.2801740
H	1.1533730	-3.2235680	-1.3071610
H	1.5957050	-3.9275860	0.2449550
C	-2.2434410	-2.5297220	-0.2004270
C	-3.4421040	-3.2215860	-0.1196110
C	-3.3529100	-0.6527960	-1.0100260
C	-4.6131060	-2.6018280	-0.5085020
H	-3.4397090	-4.2421930	0.2369310
C	-4.5665110	-1.2972030	-0.9783510
H	-3.2609590	0.3682180	-1.3507710
H	-5.5538520	-3.1324360	-0.4534690
H	-5.4552690	-0.7802620	-1.3075270
C	2.6673790	-2.1485810	-0.2793490
C	3.5632490	-2.1773740	0.7896430
C	2.9793790	-1.3648160	-1.3889590
C	4.7185800	-1.4118850	0.7668750
H	3.3622230	-2.8203410	1.6389370
C	4.1390850	-0.6060500	-1.4188640
H	2.3025150	-1.3560140	-2.2373460
C	5.0068100	-0.6211930	-0.3368040
H	5.4015300	-1.4442380	1.6055930

H	4.3675700	-0.0063300	-2.2907620
H	5.9131800	-0.0302030	-0.3590680
N	-1.5828120	1.8193830	-0.3086940
N	1.1468490	1.1107790	-0.3822770
N	-2.2142850	-1.2512190	-0.6113720
Mn	-0.4428600	-0.1845980	-0.4712570
O	-0.3138040	-0.3753690	-2.3179990

63

Mn_IV_BnTPEN_Oxo_G3_Doublet.log

63

Final Energy = -2546.4441380600

C	3.2456660	0.1051560	0.7872270
C	3.4341950	-0.4184660	-1.4733520
C	4.8055580	-0.5125190	-1.3714330
C	5.4066670	-0.2928260	-0.1429840
C	4.6146340	0.0125410	0.9499690
H	2.9133540	-0.5868250	-2.4055240
H	5.3850330	-0.7611830	-2.2480210
H	6.4796830	-0.3678260	-0.0347060
H	5.0428920	0.1765140	1.9286020
C	2.2954790	0.4642920	1.8808230
H	2.1406260	1.5451330	1.8987180
H	2.6856520	0.1886380	2.8622130
N	1.0012820	-0.1865160	1.5954780
C	-0.1462350	0.4771790	2.2520650
H	-0.1982640	0.2274710	3.3141220
H	-0.0037570	1.5550010	2.1841890
C	-1.3947250	0.0364790	1.5438520
H	-1.5192380	-1.0425600	1.6455870
H	-2.2844720	0.5021500	1.9691590
N	-1.3086310	0.3269810	0.0792490
C	1.0642190	-1.6394090	1.9188350
H	0.4572690	-1.8501260	2.8013740
H	2.0872690	-1.8883510	2.2058780
C	0.6677710	-2.5288620	0.7840800
C	0.5019220	-3.8933890	0.9639770
C	0.2082160	-4.6887650	-0.1262580
H	0.6109600	-4.3152880	1.9534220
C	0.2496930	-2.7382660	-1.4876480
C	0.0899230	-4.1009770	-1.3767860
H	0.0756500	-5.7548370	-0.0036300
H	0.1516440	-2.2152560	-2.4295430
H	-0.1313260	-4.6853530	-2.2575500

C	-1.6370910	1.7487030	-0.2211970
H	-2.4666640	2.0906960	0.3990960
H	-1.9856780	1.7819440	-1.2557990
C	-2.2670350	-0.5737810	-0.6760270
H	-1.9918650	-1.5943740	-0.4189470
H	-2.0501090	-0.4209040	-1.7324000
C	-0.4627480	2.6565450	-0.1196170
C	-0.5992260	4.0174650	0.0931200
C	1.8234060	2.8785260	-0.4010560
C	0.5250940	4.8203060	0.0483910
H	-1.5790990	4.4315840	0.2833800
C	1.7567460	4.2422860	-0.2149160
H	2.7652840	2.3885540	-0.6054750
H	0.4413410	5.8857780	0.2125430
H	2.6587400	4.8329590	-0.2719780
C	-3.7163190	-0.3292990	-0.3793220
C	-4.4681770	0.5213510	-1.1880540
C	-4.3433560	-0.9629660	0.6926990
C	-5.8077490	0.7517680	-0.9192770
H	-4.0034380	0.9933410	-2.0461730
C	-5.6822440	-0.7322650	0.9642340
H	-3.7834610	-1.6556440	1.3101330
C	-6.4154400	0.1299150	0.1616540
H	-6.3790040	1.4108950	-1.5595530
H	-6.1559140	-1.2341280	1.7976090
H	-7.4623140	0.3068740	0.3708300
N	2.6694960	-0.1060830	-0.4143520
N	0.5248320	-1.9641440	-0.4243340
N	0.7373470	2.0941100	-0.3416600
Mn	0.6717720	0.0555850	-0.5571780
O	0.4301960	0.1013410	-2.1320950

63

Mn_IV_BnTPEN_Oxo_G3_Quartet.log

63

Final Energy = -2546.4649867100

C	3.2829040	0.1057220	0.7371470
C	3.4304160	-0.3171060	-1.5566560
C	4.8040300	-0.4123820	-1.4864110
C	5.4228610	-0.2433380	-0.2582490
C	4.6552550	0.0142780	0.8659880
H	2.8737110	-0.4365220	-2.4770810
H	5.3719370	-0.6189290	-2.3811460
H	6.4982950	-0.3179500	-0.1733440

H	5.1082200	0.1414420	1.8389850
C	2.3596720	0.4345100	1.8716430
H	2.2086410	1.5154630	1.9163280
H	2.7925650	0.1435230	2.8302090
N	1.0512390	-0.2060530	1.6330970
C	-0.0966440	0.4742080	2.2760300
H	-0.1578880	0.2340130	3.3399620
H	0.0590850	1.5493530	2.2032520
C	-1.3650860	0.0422170	1.5822650
H	-1.5071130	-1.0314730	1.7145530
H	-2.2363350	0.5314330	2.0204390
N	-1.3203280	0.2890150	0.1043060
C	1.0991610	-1.6561330	1.9566040
H	0.4872570	-1.8661390	2.8357590
H	2.1184390	-1.9194930	2.2447890
C	0.6958440	-2.5341310	0.8164060
C	0.5218260	-3.8997670	0.9720810
C	0.2239330	-4.6720060	-0.1341970
H	0.6286550	-4.3409200	1.9531100
C	0.2743600	-2.7047910	-1.4694140
C	0.1086260	-4.0671900	-1.3778660
H	0.0860340	-5.7394450	-0.0290930
H	0.1817300	-2.1510580	-2.3947210
H	-0.1149580	-4.6391770	-2.2659100
C	-1.6684210	1.6906400	-0.2469710
H	-2.5345120	2.0274540	0.3249750
H	-1.9644970	1.6860700	-1.2987520
C	-2.2637650	-0.6451600	-0.6180130
H	-2.0048030	-1.6524930	-0.2969970
H	-2.0129780	-0.5498360	-1.6746450
C	-0.5222280	2.6279020	-0.1143630
C	-0.6944020	3.9870190	0.0791210
C	1.7707340	2.9017880	-0.3227990
C	0.4113660	4.8162010	0.0620400
H	-1.6895230	4.3787920	0.2334290
C	1.6638480	4.2651330	-0.1543380
H	2.7303200	2.4339350	-0.4906970
H	0.2973680	5.8808760	0.2118380
H	2.5537710	4.8754120	-0.1878250
C	-3.7184870	-0.3764450	-0.3706770
C	-4.4530870	0.4046570	-1.2607320
C	-4.3647190	-0.9132970	0.7417580
C	-5.7962840	0.6601940	-1.0349970

H -3.9703110 0.8038150 -2.1454590
C -5.7073030 -0.6574190 0.9701870
H -3.8175000 -1.5512760 1.4256740
C -6.4243570 0.1336600 0.0842750
H -6.3542370 1.2648780 -1.7377550
H -6.1962090 -1.0846360 1.8358110
H -7.4739790 0.3298470 0.2598950
N 2.6946110 -0.0589400 -0.4677720
N 0.5532280 -1.9571560 -0.3876500
N 0.7000900 2.0947080 -0.2893970
Mn 0.6840560 0.0534380 -0.4938930
O 0.4106520 0.1102870 -2.1369410
63
Mn_IV_BnTPEN_Oxo_G3_Sextet.log
63
Final Energy = -2546.4131072400
C 3.4210250 -0.0187650 0.9463500
C 3.9294840 -0.4275020 -1.2725510
C 5.2721250 -0.5685370 -0.9820890
C 5.6843800 -0.4288410 0.3332210
C 4.7466300 -0.1559820 1.3154360
H 3.5500630 -0.5308750 -2.2819210
H 5.9751930 -0.7876170 -1.7720930
H 6.7279320 -0.5393590 0.5949600
H 5.0340040 -0.0525860 2.3524730
C 2.3307630 0.3250070 1.9130640
H 2.1888840 1.4077240 1.9430660
H 2.5776160 0.0124570 2.9295510
N 1.0466620 -0.2845250 1.4789910
C -0.0985250 0.3554000 2.1848730
H -0.0939460 0.0623490 3.2368150
H 0.0650570 1.4327190 2.1564990
C -1.4063070 -0.0136440 1.5370830
H -1.5620250 -1.0922760 1.6004340
H -2.2305750 0.4498740 2.0873630
N -1.4232420 0.3543900 0.1072390
C 1.0866800 -1.7661360 1.7013170
H 0.6167310 -2.0083150 2.6558070
H 2.1346840 -2.0581280 1.7971190
C 0.4962630 -2.5663930 0.5858770
C 0.0830780 -3.8766440 0.7567880
C -0.3458500 -4.5956710 -0.3444510
H 0.1076760 -4.3212930 1.7414580

C	0.0432960	-2.6802060	-1.6965160
C	-0.3558570	-3.9942580	-1.5941200
H	-0.6706020	-5.6205570	-0.2284190
H	0.0300420	-2.1457340	-2.6384800
H	-0.6796080	-4.5270640	-2.4754530
C	-1.6727470	1.7878390	-0.1114890
H	-2.4005550	2.1855830	0.6015800
H	-2.1335630	1.8926340	-1.0968980
C	-2.4099370	-0.4717820	-0.6520750
H	-2.1531620	-1.5144350	-0.4643800
H	-2.2311780	-0.2732890	-1.7098360
C	-0.4485330	2.6512010	-0.1291970
C	-0.5325110	4.0115200	0.1196850
C	1.8138720	2.8615790	-0.6405430
C	0.5908660	4.8024110	-0.0296650
H	-1.4799730	4.4357240	0.4212120
C	1.7851220	4.2200700	-0.4281680
H	2.7205340	2.3530430	-0.9407650
H	0.5359740	5.8657130	0.1598000
H	2.6832550	4.8030340	-0.5656960
C	-3.8491280	-0.2257060	-0.2977310
C	-4.6176430	0.6748220	-1.0324830
C	-4.4414100	-0.8871770	0.7771700
C	-5.9374610	0.9255520	-0.6901390
H	-4.1817490	1.1723130	-1.8916190
C	-5.7601780	-0.6375090	1.1227390
H	-3.8694620	-1.6171420	1.3386870
C	-6.5095740	0.2737470	0.3923920
H	-6.5215320	1.6246180	-1.2743610
H	-6.2059730	-1.1621810	1.9577450
H	-7.5406650	0.4658690	0.6590760
N	3.0236460	-0.1525180	-0.3286320
N	0.4574760	-1.9850140	-0.6273370
N	0.7226900	2.0911660	-0.4819300
Mn	0.8172500	0.0312770	-0.6050200
O	0.5496890	0.2118020	-2.4394020
74			
Mn_IV_MeTBEN_Oxo_G1_Quartet.log			
74			
Final Energy =	-2828.1972252900		
C	-0.8203980	0.4696340	2.2666570
H	-1.8436700	0.0980230	2.1713360
H	-0.6922370	0.7804370	3.3054100

N	0.1112090	-0.6097480	1.8823150
C	-0.3503600	-1.9766430	2.2107900
H	-0.1846090	-2.2128410	3.2645880
H	-1.4251100	-2.0193230	2.0405290
C	0.3895370	-2.9638870	1.3414820
H	1.4627050	-2.8895890	1.5193140
H	0.1039680	-3.9882050	1.5882350
N	0.1757720	-2.7131790	-0.1182780
C	1.4814140	-0.3414020	2.3924060
H	1.7377240	-1.0473230	3.1861820
H	1.5107480	0.6478340	2.8543930
C	-1.1278290	-3.2587460	-0.5777450
H	-1.3639660	-4.1822270	-0.0443430
H	-1.0286840	-3.5204490	-1.6333610
Mn	0.1324910	-0.5786320	-0.3670060
O	0.2709000	-0.5688030	-2.0226200
C	-2.1851330	-2.2372430	-0.4493520
N	-1.8762230	-0.9510750	-0.3715590
N	-3.5101040	-2.4456860	-0.4929970
C	-3.0784940	-0.2576950	-0.3960420
C	-4.1151180	-1.2017140	-0.4571600
C	-4.2051910	-3.7112680	-0.6102700
C	-3.3788510	1.1014140	-0.3949290
C	-5.4536690	-0.8455540	-0.4867590
H	-4.9225410	-3.8134820	0.2003450
H	-4.7300680	-3.7612510	-1.5619490
H	-3.4941890	-4.5277290	-0.5577890
C	-4.7140210	1.4617800	-0.4251470
H	-2.6098350	1.8590390	-0.4083320
C	-5.7354730	0.5078080	-0.4626410
H	-6.2378630	-1.5884390	-0.5338040
H	-4.9729230	2.5118820	-0.4315970
H	-6.7657700	0.8352470	-0.4864090
C	-0.6019690	1.5823760	1.3044380
N	-0.1476490	1.3208560	0.0802220
N	-0.8376310	2.8921950	1.4731620
C	-0.0740690	2.5222820	-0.5929810
C	-0.5151340	3.5255010	0.2798360
C	-1.3709020	3.5504540	2.6487120
C	0.3385250	2.8201840	-1.8843570
C	-0.5674680	4.8584560	-0.0984290
H	-0.7132400	4.3648110	2.9418450
H	-2.3608390	3.9476590	2.4334070

H	-1.4437170	2.8420730	3.4658060
C	0.2887150	4.1487080	-2.2628370
H	0.6748190	2.0342390	-2.5474370
C	-0.1560120	5.1475770	-1.3872290
H	-0.9107630	5.6312330	0.5751000
H	0.5992830	4.4270910	-3.2607330
H	-0.1791100	6.1736110	-1.7281960
C	2.4629540	-0.3906060	1.2839040
N	2.0719770	-0.4698170	0.0182870
N	3.7974130	-0.3165260	1.3922800
C	3.2179300	-0.4307730	-0.7598450
C	4.3152240	-0.3364420	0.1046830
C	4.5782110	-0.2110070	2.6078420
C	3.3942810	-0.4777490	-2.1360100
C	5.6199330	-0.2824570	-0.3586840
H	5.3332800	-0.9930420	2.6257600
H	5.0671410	0.7598280	2.6552750
H	3.9324150	-0.3251860	3.4710490
C	4.6955810	-0.4225200	-2.6023660
H	2.5377720	-0.5515860	-2.7914840
C	5.7872400	-0.3269200	-1.7317530
H	6.4624880	-0.2065660	0.3145890
H	4.8762470	-0.4532700	-3.6684000
H	6.7872260	-0.2857880	-2.1416910
C	1.2555290	-3.3517340	-0.9022460
H	1.1566800	-3.0479440	-1.9411160
H	2.2195160	-3.0245360	-0.5257220
H	1.1943910	-4.4380280	-0.8225420

74

Mn_IV_MeTBEN_Oxo_G2_Quartet.log

74

Final Energy = -2828.1972255500

C	-0.8204640	0.4695170	2.2666090
H	-1.8436820	0.0977590	2.1713150
H	-0.6923140	0.7803470	3.3053600
N	0.1112920	-0.6097290	1.8822400
C	-0.3501260	-1.9767060	2.2106580
H	-0.1843450	-2.2129080	3.2644490
H	-1.4248680	-2.0195020	2.0403960
C	0.3898880	-2.9638490	1.3413180
H	1.4630500	-2.8894660	1.5191750
H	0.1044110	-3.9882020	1.5880220
N	0.1761360	-2.7131110	-0.1184340

C	1.4814290	-0.3412080	2.3923870
H	1.7377880	-1.0470200	3.1862420
H	1.5105950	0.6480860	2.8542730
C	-1.1273910	-3.2588160	-0.5779620
H	-1.3633920	-4.1823570	-0.0446010
H	-1.0281880	-3.5204580	-1.6335780
Mn	0.1326320	-0.5785760	-0.3670790
O	0.2711100	-0.5687840	-2.0226850
C	-2.1848160	-2.2374600	-0.4494970
N	-1.8760340	-0.9512610	-0.3716630
N	-3.5097620	-2.4460360	-0.4930220
C	-3.0783760	-0.2580040	-0.3959540
C	-4.1149040	-1.2021290	-0.4569970
C	-4.2047650	-3.7116240	-0.6105330
C	-3.3788890	1.1010700	-0.3948030
C	-5.4535010	-0.8461140	-0.4865730
H	-4.9215890	-3.8143470	0.2004870
H	-4.7302560	-3.7611230	-1.5618970
H	-3.4936360	-4.5280290	-0.5589380
C	-4.7140950	1.4612890	-0.4249350
H	-2.6099710	1.8587890	-0.4082580
C	-5.7354490	0.5072130	-0.4624030
H	-6.2376030	-1.5890980	-0.5335930
H	-4.9731160	2.5113630	-0.4313260
H	-6.7657790	0.8345530	-0.4861160
C	-0.6022320	1.5823230	1.3044170
N	-0.1477770	1.3208830	0.0802340
N	-0.8383310	2.8920820	1.4730540
C	-0.0744100	2.5223030	-0.5929940
C	-0.5158040	3.5254220	0.2797550
C	-1.3717350	3.5503570	2.6485310
C	0.3382510	2.8202940	-1.8843230
C	-0.5683580	4.8583570	-0.0984740
H	-0.7128540	4.3631870	2.9432150
H	-2.3605810	3.9497360	2.4322560
H	-1.4471360	2.8414100	3.4648810
C	0.2881670	4.1488090	-2.2628120
H	0.6747820	2.0344210	-2.5473710
C	-0.1568580	5.1475780	-1.3872400
H	-0.9118430	5.6310320	0.5750730
H	0.5987620	4.4272610	-3.2606790
H	-0.1801400	6.1736150	-1.7281880
C	2.4630120	-0.3904270	1.2839510

N	2.0720900	-0.4695670	0.0183170
N	3.7974680	-0.3164460	1.3924250
C	3.2180820	-0.4304120	-0.7597490
C	4.3153340	-0.3361080	0.1048400
C	4.5780710	-0.2108770	2.6081130
C	3.3944910	-0.4772810	-2.1359110
C	5.6200550	-0.2819560	-0.3584850
H	5.3350400	-0.9910820	2.6246580
H	5.0646450	0.7610710	2.6571600
H	3.9326460	-0.3282070	3.4711780
C	4.6957990	-0.4219260	-2.6022120
H	2.5380110	-0.5511410	-2.7914220
C	5.7874130	-0.3262840	-1.7315500
H	6.4625880	-0.2058910	0.3147930
H	4.8765090	-0.4525660	-3.6682420
H	6.7874100	-0.2849790	-2.1414460
C	1.2559770	-3.3515700	-0.9023670
H	1.1569810	-3.0480010	-1.9412850
H	2.2199220	-3.0240850	-0.5259880
H	1.1951000	-4.4378690	-0.8224420

74

Mn_IV_MeTBEN_Oxo_G2_Sextet.log

74

Final Energy = -2828.1502375000

C	-0.6885700	0.6368560	2.3748040
H	-1.6542650	0.1267930	2.3871890
H	-0.4897410	0.9610050	3.3990150
N	0.3364580	-0.3312970	1.9120670
C	0.1267380	-1.6636760	2.5395620
H	0.4106060	-1.6339490	3.5938650
H	-0.9446090	-1.8692410	2.5045220
C	0.8910000	-2.7359320	1.8070730
H	1.9567060	-2.5025790	1.7794410
H	0.7982680	-3.6851530	2.3446000
N	0.4228110	-2.8469070	0.4164960
C	1.7048940	0.2008690	2.1957410
H	2.0986790	-0.2521630	3.1082670
H	1.6301560	1.2726520	2.3936070
C	-0.8944380	-3.4947670	0.3221080
H	-1.1589950	-3.9927680	1.2604940
H	-0.8609170	-4.2885650	-0.4273590
Mn	0.1553090	-0.5464700	-0.2793250
O	0.1405650	-0.8562770	-2.0937130

C	-1.9605930	-2.5322580	-0.0551820
N	-1.7313490	-1.2462510	-0.3352750
N	-3.2579860	-2.8398670	-0.1696260
C	-2.9581900	-0.6921980	-0.6779820
C	-3.9302180	-1.6906760	-0.5634580
C	-3.8792590	-4.1272720	0.0661080
C	-3.3060790	0.5875590	-1.0878540
C	-5.2692120	-1.4609060	-0.8226710
H	-4.5776660	-4.0554310	0.8969110
H	-4.4156850	-4.4418760	-0.8259500
H	-3.1212450	-4.8654560	0.3006640
C	-4.6445340	0.8252750	-1.3523100
H	-2.5617520	1.3602300	-1.2171880
C	-5.6085580	-0.1766580	-1.2184280
H	-6.0120120	-2.2400460	-0.7241280
H	-4.9500710	1.8105610	-1.6769020
H	-6.6430510	0.0520860	-1.4347910
C	-0.7181480	1.7644430	1.4089910
N	-0.3237890	1.5739240	0.1630440
N	-1.1350390	3.0260840	1.6419230
C	-0.4843810	2.7886070	-0.4726190
C	-1.0013630	3.7128310	0.4464020
C	-1.6384640	3.5895870	2.8766330
C	-0.2298590	3.1622200	-1.7871110
C	-1.2791960	5.0267550	0.1013340
H	-1.0319020	4.4437220	3.1694660
H	-2.6692680	3.9127640	2.7467380
H	-1.6022550	2.8449750	3.6636690
C	-0.5057810	4.4713480	-2.1372820
H	0.1664260	2.4508200	-2.5001060
C	-1.0200190	5.3863240	-1.2099380
H	-1.6776410	5.7345630	0.8152460
H	-0.3212670	4.7996800	-3.1512900
H	-1.2210970	6.4006530	-1.5272340
C	2.6109020	-0.0062160	1.0407210
N	2.1418950	-0.2946830	-0.1685590
N	3.9482510	0.1137700	1.0430520
C	3.2334850	-0.3661040	-1.0140960
C	4.3811390	-0.1063470	-0.2564790
C	4.8113260	0.4326560	2.1625800
C	3.3195110	-0.6393200	-2.3726320
C	5.6496340	-0.1012810	-0.8141380
H	5.5668850	-0.3413530	2.2739400

H	5.3007500	1.3891660	1.9924260
H	4.2287310	0.4918420	3.0745820
C	4.5836250	-0.6360780	-2.9333810
H	2.4309670	-0.8436190	-2.9555370
C	5.7263600	-0.3710550	-2.1691280
H	6.5319430	0.1035000	-0.2238930
H	4.6945620	-0.8422810	-3.9892400
H	6.6947830	-0.3774580	-2.6505000
C	1.4097100	-3.5239860	-0.4339810
H	1.0766990	-3.4892880	-1.4686230
H	2.3618940	-3.0032760	-0.3640900
H	1.5606030	-4.5655330	-0.1371720

74

Mn_IV_MeTBEN_Oxo_G3_Sextet.log

74

Final Energy = -2828.1502394000

C	-0.6863710	0.6383030	2.3763780
H	-1.6522110	0.1285490	2.3893080
H	-0.4871990	0.9629340	3.4004050
N	0.3380820	-0.3304740	1.9137720
C	0.1288330	-1.6621360	2.5428600
H	0.4149000	-1.6317180	3.5965580
H	-0.9426910	-1.8670880	2.5102040
C	0.8911000	-2.7355110	1.8098740
H	1.9569360	-2.5029910	1.7805490
H	0.7984460	-3.6843140	2.3481670
N	0.4205910	-2.8467850	0.4202320
C	1.7068680	0.2018020	2.1952730
H	2.1017010	-0.2504080	3.1077610
H	1.6325730	1.2737800	2.3922660
C	-0.8973840	-3.4935050	0.3282550
H	-1.1626850	-3.9868770	1.2688850
H	-0.8648560	-4.2908970	-0.4174850
Mn	0.1546570	-0.5466110	-0.2780690
O	0.1388720	-0.8590140	-2.0921800
C	-1.9623750	-2.5312690	-0.0529290
N	-1.7323180	-1.2454410	-0.3332710
N	-3.2595720	-2.8387280	-0.1700220
C	-2.9584590	-0.6911390	-0.6779570
C	-3.9309490	-1.6893020	-0.5646020
C	-3.8818610	-4.1252430	0.0683020
C	-3.3054010	0.5885470	-1.0889120
C	-5.2694760	-1.4592350	-0.8259160

H	-4.5595630	-4.0590840	0.9166830
H	-4.4415240	-4.4271390	-0.8136810
H	-3.1222410	-4.8699910	0.2754720
C	-4.6433950	0.8265820	-1.3553660
H	-2.5606630	1.3609570	-1.2174990
C	-5.6079230	-0.1750240	-1.2225670
H	-6.0125020	-2.2382350	-0.7280470
H	-4.9481560	1.8118790	-1.6806570
H	-6.6420440	0.0539790	-1.4404080
C	-0.7160280	1.7654400	1.4100750
N	-0.3232630	1.5739970	0.1637460
N	-1.1311680	3.0276840	1.6429380
C	-0.4833000	2.7885780	-0.4722840
C	-0.9983820	3.7136950	0.4469030
C	-1.6340900	3.5919710	2.8774990
C	-0.2297480	3.1613660	-1.7872010
C	-1.2753400	5.0277130	0.1015310
H	-1.0317440	4.4507430	3.1652900
H	-2.6673300	3.9084300	2.7502340
H	-1.5902500	2.8503080	3.6669240
C	-0.5047600	4.4706090	-2.1376650
H	0.1650850	2.4492260	-2.5002640
C	-1.0171680	5.3864660	-1.2101750
H	-1.6725750	5.7361650	0.8154750
H	-0.3209950	4.7983440	-3.1520000
H	-1.2176860	6.4008300	-1.5277110
C	2.6115050	-0.0064810	1.0393950
N	2.1411040	-0.2957480	-0.1691590
N	3.9489170	0.1128630	1.0401990
C	3.2317770	-0.3684320	-1.0157850
C	4.3803420	-0.1086160	-0.2595600
C	4.8134030	0.4322130	2.1584930
C	3.3161970	-0.6427780	-2.3741990
C	5.6482360	-0.1045840	-0.8186150
H	5.5684540	-0.3422860	2.2699260
H	5.3034230	1.3881590	1.9868750
H	4.2317910	0.4928400	3.0710230
C	4.5797020	-0.6405540	-2.9363210
H	2.4268860	-0.8470940	-2.9559330
C	5.7233680	-0.3754590	-2.1734780
H	6.5313030	0.1002470	-0.2295270
H	4.6894430	-0.8476380	-3.9921320
H	6.6912730	-0.3826760	-2.6558790

C	1.4054140	-3.5245150	-0.4321290
H	1.0709030	-3.4886590	-1.4662500
H	2.3583430	-3.0050450	-0.3632710
H	1.5554200	-4.5664390	-0.1362120

TS_MnBnTBEN_Thioanisole_Quartet
100
2 4

C	-0.19929300	1.86024700	-2.60496500
H	0.89143300	1.83521700	-2.76180100
H	-0.63625200	2.23892700	-3.54293200
N	-0.64666200	0.47600000	-2.32192200
C	0.02903700	-0.47196700	-3.23822000
H	-0.36483900	-0.36424400	-4.26265100
H	1.08997000	-0.18762400	-3.29166500
C	-0.13326100	-1.89599100	-2.77668400
H	-1.20283300	-2.13297100	-2.68264200
H	0.25900600	-2.57828700	-3.55493500
N	0.49956900	-2.15024100	-1.47669500
C	-2.12890400	0.36107500	-2.43079400
H	-2.39569600	-0.39708000	-3.18445000
H	-2.55203300	1.30246500	-2.81988000
C	1.96226600	-2.15649400	-1.59288200
H	2.29592500	-2.52891300	-2.58013100
H	2.36924700	-2.87857800	-0.86231100
C	0.07139300	-3.46403100	-0.92448400
H	0.62025700	-3.59643000	0.01884900
H	0.41254800	-4.26806500	-1.60516700
C	-1.40009800	-3.61217200	-0.67214200
C	-2.24713200	-4.16199200	-1.64612700
C	-1.94921800	-3.23880100	0.56005400
C	-3.61215400	-4.30934200	-1.40622900
H	-1.82743100	-4.48291200	-2.60438300
C	-3.31153000	-3.40165900	0.80958800
H	-1.30007100	-2.80076200	1.32670900
C	-4.14740100	-3.92774000	-0.17490300
H	-4.25755500	-4.73384600	-2.17850200
H	-3.72547900	-3.10175200	1.77609100
H	-5.21607900	-4.04658400	0.02048300
Mn	-0.02406400	0.00626700	-0.20564400
O	0.25963400	-0.45356200	1.57349600
C	2.58033300	-0.84262000	-1.28773500
N	1.94917800	0.15023100	-0.66692700

N	3.88638800	-0.56122900	-1.50767100
C	2.90385900	1.13467600	-0.43977600
C	4.13016400	0.69596700	-0.98103700
C	4.85410600	-1.41506400	-2.16201000
C	2.82946200	2.35761700	0.23576100
C	5.29904000	1.45165800	-0.89638700
H	4.81441900	-1.29880700	-3.25300500
H	5.85812200	-1.15213200	-1.81488600
H	4.67336900	-2.46445600	-1.90503200
C	3.99543200	3.10986100	0.33193100
H	1.89853800	2.69707700	0.68996700
C	5.20773100	2.66869700	-0.22897400
H	6.24168900	1.10604100	-1.32359100
H	3.96994500	4.06498600	0.85979800
H	6.09894900	3.29173200	-0.13339700
C	-0.50223700	2.72729200	-1.43517300
N	-0.40391900	2.23313400	-0.21669000
N	-0.86985600	4.03758300	-1.44535700
C	-0.73537000	3.25640500	0.64068000
C	-1.02036600	4.41147200	-0.11835700
C	-1.06408600	4.91364800	-2.57850800
C	-0.81038100	3.28145800	2.03711800
C	-1.37749600	5.62193000	0.47612700
H	-1.01875400	4.34230400	-3.50914800
H	-2.04625000	5.39698200	-2.51459000
H	-0.28849700	5.68954800	-2.60665400
C	-1.16740900	4.48550500	2.63412200
H	-0.59861700	2.38129900	2.61908000
C	-1.44421100	5.63401200	1.86698800
H	-1.59278800	6.51485700	-0.11336700
H	-1.23658000	4.54607400	3.72211400
H	-1.71958500	6.55940500	2.37680000
C	-2.75433200	0.04967000	-1.11948100
N	-2.04330000	-0.12094600	-0.01313500
N	-4.08695300	-0.00104500	-0.88622700
C	-2.95249100	-0.24590200	1.02229700
C	-4.25034000	-0.18867100	0.47792900
C	-5.16233900	0.08022000	-1.84879100
C	-2.76912000	-0.41464800	2.39734100
C	-5.39758000	-0.31984900	1.25854000
H	-5.87183100	0.86413600	-1.55813300
H	-4.76739700	0.31976100	-2.83944500
H	-5.69502400	-0.87831700	-1.90577500

C	-3.91017500	-0.53286100	3.18412200
H	-1.76440900	-0.45773500	2.81519400
C	-5.20140400	-0.49268300	2.62585700
H	-6.39770300	-0.28589600	0.82325600
H	-3.80342900	-0.66409200	4.26291800
H	-6.06969200	-0.59490800	3.27976500
C	2.82200400	-1.43893000	2.11744600
C	4.12632700	-1.02939700	1.78883500
C	5.05920100	-1.97987400	1.38959600
C	4.70378100	-3.33149900	1.31441500
C	3.40437400	-3.73172000	1.64271500
C	2.45523200	-2.79421500	2.03891700
H	4.40256200	0.02589800	1.86089800
H	6.07481700	-1.66333900	1.13931700
H	5.44086400	-4.07392200	1.00255200
H	3.12546500	-4.78637000	1.58510600
H	1.43855400	-3.10908300	2.28713500
S	1.65857500	-0.19130600	2.52839800
C	0.90221100	-0.82770900	4.02053400
H	1.67504600	-0.95457000	4.78708200
H	0.17016800	-0.08338900	4.35214600
H	0.39838700	-1.77681600	3.8008410090

Mn_IV_BnTBEN_Oxo_G1_Doublet.log

84

Final Energy = -3058.8821019500

C	1.8973300	0.9997450	-1.8827420
H	2.7363180	0.3404150	-1.6253320
H	2.2167790	1.5956530	-2.7470790
N	0.6943550	0.1816860	-2.1399250
C	0.9362170	-1.0996830	-2.8461870
H	1.0447520	-0.9509030	-3.9293110
H	1.8785920	-1.5259210	-2.4876810
C	-0.2506090	-1.9969980	-2.5474920
H	-1.1865710	-1.5532550	-2.9061080
H	-0.1419640	-2.9683550	-3.0468480
N	-0.3910040	-2.1947770	-1.0620620
C	-0.3630600	0.9661840	-2.8287810
H	-0.6228450	0.5425120	-3.8060990
H	0.0007540	1.9799510	-3.0405440
C	0.6763420	-3.1136240	-0.5767820
H	0.9475930	-3.8624140	-1.3307170
H	0.3035170	-3.6656770	0.2892070
C	-1.7805440	-2.7722960	-0.7942890

H	-1.8416740	-3.7040630	-1.3737470
H	-2.4945050	-2.0571650	-1.2166580
C	-2.1032720	-3.0463090	0.6484830
C	-1.7393870	-4.2286870	1.2844620
C	-2.7499970	-2.0293210	1.4121640
C	-1.9492020	-4.3896810	2.6641550
H	-1.2830160	-5.0408720	0.7183640
C	-2.9553210	-2.2016600	2.8088990
H	-3.1206250	-1.1174050	0.9545030
C	-2.5398970	-3.3652310	3.4285090
H	-1.6419410	-5.3174110	3.1464790
H	-3.4593430	-1.4129190	3.3661010
H	-2.6949130	-3.5089070	4.4972970
Mn	-0.0046660	-0.3374660	-0.1648410
O	-0.6520920	-0.6246350	1.3268590
C	1.8594770	-2.2803290	-0.1149740
N	1.7327720	-1.2194390	0.6419110
N	3.2497480	-2.5204930	-0.4513740
C	3.0872240	-0.7234860	0.8252810
C	3.9618900	-1.5495330	0.1444100
C	3.7745830	-3.6116910	-1.2820810
C	3.5274450	0.3836750	1.5450940
C	5.3373390	-1.3169990	0.1424400
H	3.8056890	-3.3001100	-2.3128820
H	4.7679040	-3.8634380	-0.9534190
H	3.1382310	-4.4738670	-1.1868160
C	4.9093800	0.6409120	1.5599210
H	2.8362420	1.0176240	2.0695130
C	5.8083790	-0.2053870	0.8621920
H	6.0122390	-1.9621400	-0.3892130
H	5.2873420	1.4865400	2.1047670
H	6.8619270	0.0053740	0.8833110
C	1.5036300	1.8405150	-0.6839650
N	0.9473220	1.3103260	0.3752220
N	1.6579770	3.2730140	-0.5366690
C	0.7150490	2.4253910	1.2766820
C	1.1654540	3.5857100	0.6736710
C	2.2373380	4.1994010	-1.5174780
C	0.1454980	2.4259710	2.5466740
C	1.0718800	4.8236710	1.3100020
H	1.4625290	4.5697580	-2.1678050
H	2.6963330	5.0240150	-1.0002890
H	2.9811160	3.6861060	-2.1011830

C	0.0398130	3.6608190	3.2097730
H	-0.2001600	1.5163280	3.0026580
C	0.5007700	4.8523520	2.5938660
H	1.4226300	5.7223270	0.8371200
H	-0.3942980	3.7018330	4.1919300
H	0.4123290	5.7883520	3.1143540
C	-1.5642350	1.0304030	-1.9036200
N	-1.4535160	0.9752980	-0.5993880
N	-2.9497730	1.1386310	-2.3089630
C	-2.8180950	1.0520120	-0.1030960
C	-3.6790440	1.1505790	-1.1802700
C	-3.4546520	1.2184930	-3.6853890
C	-3.2779010	1.0367940	1.2105740
C	-5.0601390	1.2400500	-1.0069290
H	-3.6491050	0.2263580	-4.0573930
H	-4.3652710	1.7918120	-3.6981340
H	-2.7216200	1.6989420	-4.3095460
C	-4.6661040	1.1259580	1.4133640
H	-2.5969540	0.9597720	2.0383840
C	-5.5512210	1.2269460	0.3100830
H	-5.7247630	1.3158150	-1.8476240
H	-5.0593560	1.1173110	2.4133670
H	-6.6098220	1.2940570	0.4822220

84
Mn_IV_BnTBEN_Oxo_G1_Quartet.log
84
Final Energy = -3059.0150118300

C	2.1103300	0.3659220	-2.1424730
H	2.4179710	-0.6834690	-2.1499650
H	2.5747480	0.8553490	-3.0037280
N	0.6293290	0.4311630	-2.1960820
C	-0.0124940	-0.6261810	-3.0117550
H	0.0306980	-0.3859570	-4.0808340
H	0.5414780	-1.5536770	-2.8638830
C	-1.4642590	-0.7707280	-2.5820190
H	-1.9849430	0.1831290	-2.6913400
H	-1.9742710	-1.4891020	-3.2336960
N	-1.6253260	-1.1983720	-1.1500610
C	0.1725490	1.7965420	-2.5856370
H	-0.2574670	1.7808360	-3.5924300
H	1.0339280	2.4701390	-2.6252360
C	-1.2933540	-2.6429070	-1.0088570
H	-1.5495940	-3.1921370	-1.9211710

H	-1.8985750	-3.0623230	-0.2006750
C	-3.0765590	-0.9132050	-0.7841610
H	-3.6752080	-1.0929380	-1.6833140
H	-3.1213160	0.1545350	-0.5657370
C	-3.6637930	-1.7267760	0.3418110
C	-4.6361360	-2.6930500	0.0425280
C	-3.2936000	-1.5290870	1.6800250
C	-5.2285350	-3.4493160	1.0569550
H	-4.9317430	-2.8543250	-0.9913570
C	-3.8817800	-2.2872490	2.6941280
H	-2.5226530	-0.8054230	1.9172850
C	-4.8510470	-3.2478440	2.3872460
H	-5.9813680	-4.1917600	0.8077140
H	-3.5828660	-2.1258830	3.7263050
H	-5.3088610	-3.8341240	3.1791330
Mn	-0.0496290	0.0129570	-0.0358460
O	-0.5405330	-0.1184290	1.5311850
C	0.1370160	-2.8073980	-0.6330300
N	0.8464480	-1.7987250	-0.1219820
N	0.8392280	-3.9613490	-0.6464950
C	2.0840180	-2.3270310	0.2634760
C	2.0873220	-3.6955990	-0.0833230
C	0.3923780	-5.2747840	-1.1032890
C	3.1956390	-1.7610140	0.8979310
C	3.1802030	-4.5298630	0.1533820
H	1.1098080	-5.6681600	-1.8265410
H	0.3147400	-5.9548850	-0.2514860
H	-0.5816360	-5.1834460	-1.5806810
C	4.2906910	-2.5887180	1.1376280
H	3.2040480	-0.7307880	1.2247600
C	4.2884780	-3.9486400	0.7668820
H	3.1665080	-5.5802980	-0.1167650
H	5.1637520	-2.1753380	1.6330720
H	5.1620250	-4.5587270	0.9741120
C	2.4996130	0.9951490	-0.8392600
N	1.6448250	0.9537790	0.1939700
N	3.6393750	1.6300340	-0.5026540
C	2.2476500	1.6055970	1.2652820
C	3.5189650	2.0339340	0.8319480
C	4.8156280	1.8651790	-1.3385490
C	1.8022490	1.8499140	2.5662910
C	4.3965580	2.7185010	1.6744830
H	4.9915010	2.9398980	-1.4217560

H	5.6829590	1.3806460	-0.8846890
H	4.6488520	1.4461340	-2.3293180
C	2.6739070	2.5349730	3.4090850
H	0.8239200	1.5077340	2.8834620
C	3.9474480	2.9611720	2.9715930
H	5.3754850	3.0452300	1.3410400
H	2.3695620	2.7461860	4.4295310
H	4.5947030	3.4906180	3.6636960
C	-0.8025400	2.3196100	-1.5826900
N	-1.0201960	1.6907990	-0.4235870
N	-1.5126810	3.4621140	-1.6709260
C	-1.9150200	2.4774700	0.3085390
C	-2.2383210	3.5977790	-0.4834140
C	-1.5318670	4.4173130	-2.7765490
C	-2.4686560	2.3158720	1.5812290
C	-3.1228170	4.5869350	-0.0524830
H	-2.5479830	4.5002980	-3.1688840
H	-1.1917670	5.3922930	-2.4200380
H	-0.8679870	4.0736700	-3.5683750
C	-3.3521930	3.3010000	2.0179450
H	-2.2018600	1.4635580	2.1921740
C	-3.6762380	4.4155220	1.2159070
H	-3.3669890	5.4460120	-0.6679620
H	-3.8007030	3.2103730	3.0025060
H	-4.3695230	5.1595600	1.5956400

84
Mn_IV_BnTBEN_Oxo_G1_Sextet.log
84
Final Energy = -3058.9975752500

C	0.2648620	1.9499030	-2.2061890
H	1.3542010	2.0292760	-2.1461790
H	-0.0565110	2.4877210	-3.1044290
N	-0.0932610	0.5108310	-2.2770290
C	0.8193040	-0.2309850	-3.1824010
H	0.5836960	-0.0191550	-4.2326570
H	1.8353620	0.1249850	-3.0022790
C	0.7184910	-1.7217470	-2.9105160
H	-0.3127520	-2.0635610	-3.0350100
H	1.3294940	-2.2726120	-3.6389960
N	1.1290660	-2.0290000	-1.5251290
C	-1.5147310	0.3357680	-2.6952330
H	-1.5626870	-0.1948770	-3.6513230
H	-1.9712420	1.3161220	-2.8674300

C	2.5922410	-1.8958010	-1.3607560
H	3.1033470	-1.9867960	-2.3266960
H	2.9588480	-2.7120240	-0.7307990
C	0.6738270	-3.4007480	-1.1424080
H	1.1699980	-4.1475910	-1.7782970
H	-0.3997250	-3.4376140	-1.3593110
C	0.9069620	-3.7025320	0.3061270
C	1.8272110	-4.6327410	0.7130770
C	0.0421550	-2.9729310	1.3031710
C	2.0117510	-4.9561900	2.0822050
H	2.4283950	-5.1496630	-0.0324710
C	0.2617750	-3.4094060	2.7214230
H	-1.0101170	-3.1603740	1.0239920
C	1.2128290	-4.3301390	3.0700720
H	2.7602880	-5.6882070	2.3672240
H	-0.3517150	-2.9247610	3.4756030
H	1.3554940	-4.5920920	4.1152740
Mn	0.1017360	-0.3505560	-0.0778990
O	0.2455940	-1.5476880	1.2895180
C	2.9534150	-0.6127120	-0.6857090
N	2.0647490	0.1591270	-0.0568310
N	4.2095890	-0.1224270	-0.5724570
C	2.7807710	1.2098030	0.5292300
C	4.1403060	1.0396350	0.1951630
C	5.4448620	-0.6908960	-1.1050360
C	2.3815730	2.2812470	1.3348720
C	5.1354200	1.9218270	0.6175580
H	5.9334630	0.0411020	-1.7521580
H	6.1105140	-0.9555910	-0.2798610
H	5.2199060	-1.5858200	-1.6825200
C	3.3699850	3.1659480	1.7624910
H	1.3502270	2.4139240	1.6312470
C	4.7234910	2.9947940	1.4066570
H	6.1773000	1.7791900	0.3513100
H	3.0892440	4.0058670	2.3906660
H	5.4616100	3.7075640	1.7610760
C	-0.3411480	2.5062950	-0.9569730
N	-0.5188440	1.7156840	0.0922720
N	-0.7671840	3.7742530	-0.7211930
C	-1.1084500	2.4888070	1.0836710
C	-1.2627840	3.8003440	0.5838600
C	-0.7172430	4.9256430	-1.6169280
C	-1.5127140	2.1593850	2.3809950

C	-1.8199850	4.8258860	1.3504370
H	-1.7217670	5.3372660	-1.7388770
H	-0.0548690	5.6882730	-1.1997200
H	-0.3361020	4.6164220	-2.5888040
C	-2.0708140	3.1783190	3.1499740
H	-1.3917660	1.1504620	2.7629710
C	-2.2215770	4.4878690	2.6428800
H	-1.9357920	5.8339650	0.9664690
H	-2.3979460	2.9635100	4.1627290
H	-2.6609660	5.2525760	3.2760690
C	-2.3133180	-0.3776640	-1.6525450
N	-1.8105930	-0.7508880	-0.4722860
N	-3.6395720	-0.6225300	-1.7278750
C	-2.8818220	-1.2077690	0.3007540
C	-4.0405400	-1.1506780	-0.4992910
C	-4.5346470	-0.3369430	-2.8465890
C	-2.9597550	-1.6069600	1.6382560
C	-5.2960110	-1.5359510	-0.0276220
H	-5.0814020	-1.2443340	-3.1110810
H	-5.2388010	0.4493180	-2.5631430
H	-3.9533380	-0.0092240	-3.7071660
C	-4.2111520	-1.9861720	2.1190030
H	-2.0851650	-1.5896530	2.2754200
C	-5.3582150	-1.9630780	1.2981570
H	-6.1812320	-1.4928580	-0.6530970
H	-4.3071240	-2.2986880	3.1542410
H	-6.3141170	-2.2685760	1.7120300

84
Mn_IV_BnTBEN_Oxo_G2_Doublet.log
84
Final Energy = -3058.7857655500

C	-1.4427200	0.4998490	2.0036020
H	-2.4040930	-0.0150890	1.8632090
H	-1.3965100	0.8139450	3.0560670
N	-0.3437280	-0.4358060	1.6618370
C	-0.5636900	-1.8498270	2.0640340
H	-0.3506730	-1.9945120	3.1336530
H	-1.6186930	-2.1067740	1.9116570
C	0.3581120	-2.7053540	1.2183820
H	1.4081140	-2.4282010	1.3783260
H	0.2629840	-3.7696510	1.4752880
N	0.0497480	-2.5095330	-0.2401440
C	0.9567080	0.0604990	2.2054730

H	1.3643420	-0.6616880	2.9262930
H	0.7782760	0.9818560	2.7781970
C	-1.2112820	-3.2372970	-0.5888580
H	-1.3018790	-4.1445320	0.0246380
H	-1.1427790	-3.5664120	-1.6325140
C	1.1964810	-3.0504920	-1.0727420
H	1.1129160	-2.6089460	-2.0767100
H	1.0457820	-4.1337330	-1.1635500
C	2.5281750	-2.7301630	-0.4616060
C	2.8109140	-2.9687140	0.8744350
C	3.5720910	-2.2329030	-1.3107730
C	4.1164360	-2.7796360	1.3701680
H	2.0261630	-3.3117840	1.5515100
C	4.8934760	-2.0506480	-0.7948040
H	3.3967300	-1.9657230	-2.3499750
C	5.1595570	-2.3393800	0.5269580
H	4.3227600	-2.9883950	2.4215520
H	5.6681130	-1.6633530	-1.4582240
H	6.1626510	-2.2005690	0.9329210
Mn	-0.2913390	-0.4650370	-0.4888620
O	-0.1190250	-0.4051440	-2.1593780
C	-2.4716920	-2.3560980	-0.5085140
N	-2.4749230	-1.0469320	-0.5139050
N	-3.8552360	-2.8626260	-0.4140150
C	-3.8976060	-0.6774360	-0.4205460
C	-4.6626730	-1.8119370	-0.3646300
C	-4.2633290	-4.2744520	-0.3807350
C	-4.4682470	0.5919410	-0.3879000
C	-6.0501200	-1.7676390	-0.2722330
H	-4.2951720	-4.6126930	0.6338970
H	-5.2332230	-4.3758450	-0.8211050
H	-3.5586380	-4.8624730	-0.9307730
C	-5.8735030	0.6799280	-0.2944990
H	-3.8612180	1.4719720	-0.4321740
C	-6.6635430	-0.4983080	-0.2367330
H	-6.6312990	-2.6650320	-0.2296070
H	-6.3460770	1.6395220	-0.2670690
H	-7.7285340	-0.4228910	-0.1659870
C	-1.3593440	1.7078150	1.0520410
N	-1.1950670	1.6330680	-0.2446390
N	-1.4528430	3.1215760	1.4670500
C	-1.1794420	3.0337910	-0.6996150
C	-1.3384020	3.8664580	0.3759990

C	-1.6389000	3.6137750	2.8396480
C	-1.0324270	3.5288440	-1.9924120
C	-1.3628410	5.2513130	0.2452760
H	-0.6834530	3.7626580	3.2977480
H	-2.1718950	4.5412950	2.8167780
H	-2.1967790	2.8956390	3.4035190
C	-1.0517650	4.9289060	-2.1675150
H	-0.9088250	2.8693770	-2.8259140
C	-1.2167580	5.7891510	-1.0501540
H	-1.4880610	5.8859630	1.0975900
H	-0.9415170	5.3443390	-3.1473930
H	-1.2303190	6.8496860	-1.1915090
C	1.9908400	0.3804580	1.1101200
N	1.8385270	0.1250060	-0.1648300
N	3.3007520	1.0206830	1.3423680
C	3.0799230	0.6087520	-0.7926820
C	3.9055820	1.1303200	0.1674220
C	3.8368150	1.4570730	2.6397120
C	3.4449100	0.5829310	-2.1358620
C	5.1537050	1.6640620	-0.1372400
H	4.3871340	0.6553510	3.0861310
H	4.4842860	2.2963860	2.4939890
H	3.0292190	1.7371280	3.2833410
C	4.7039410	1.1150650	-2.4864080
H	2.7921920	0.1730420	-2.8780560
C	5.5573440	1.6549760	-1.4884400
H	5.7851310	2.0672460	0.6267260
H	5.0170680	1.1116490	-3.5095590
H	6.5104810	2.0565500	-1.7626080

84
Mn_IV_BnTBEN_Oxo_G2_Quartet.log
84
Final Energy = -3059.2988489200

C	-1.4767740	0.3339030	2.2715590
H	-2.3797790	-0.2690040	2.1443740
H	-1.4486330	0.6446670	3.3174530
N	-0.3095300	-0.4920940	1.8995610
C	-0.4650460	-1.9344810	2.1857250
H	-0.3035590	-2.1517290	3.2443360
H	-1.4899310	-2.2204140	1.9582060
C	0.5383170	-2.6891210	1.3529380
H	1.5373040	-2.3244420	1.5835420
H	0.5246050	-3.7545290	1.5964740

N	0.3373050	-2.4973180	-0.1118350
C	0.9527910	0.0385990	2.4814310
H	1.4121700	-0.7119680	3.1286580
H	0.7324310	0.8884180	3.1316940
C	-0.8263510	-3.3028210	-0.5754600
H	-0.8665850	-4.2518260	-0.0353090
H	-0.6677060	-3.5501500	-1.6275670
C	1.5175340	-3.0483080	-0.8859760
H	1.2708690	-2.8719470	-1.9313170
H	1.5060280	-4.1313200	-0.7209310
C	2.8874590	-2.5130590	-0.6069270
C	3.6296020	-2.8925540	0.5147860
C	3.5056640	-1.7191830	-1.5693520
C	4.9238650	-2.4327850	0.6966210
H	3.2070000	-3.5777110	1.2393660
C	4.8105350	-1.2808810	-1.4040810
H	2.9505380	-1.4425860	-2.4581210
C	5.5165670	-1.6209550	-0.2612850
H	5.4793400	-2.7304510	1.5763810
H	5.2687420	-0.6618250	-2.1644550
H	6.5314830	-1.2703950	-0.1241370
Mn	-0.2188650	-0.3674430	-0.3584540
O	-0.0054300	-0.2884230	-2.0015720
C	-2.0839670	-2.5423390	-0.4748070
N	-2.0804500	-1.2188710	-0.4423890
N	-3.3246650	-3.0543010	-0.5020920
C	-3.4127470	-0.8239450	-0.4829520
C	-4.2013980	-1.9844700	-0.5078150
C	-3.7120030	-4.4493410	-0.5494030
C	-4.0243740	0.4258060	-0.5276820
C	-5.5859600	-1.9533300	-0.5468250
H	-4.3764850	-4.6740750	0.2816050
H	-4.2240580	-4.6621040	-1.4853020
H	-2.8327220	-5.0787740	-0.4779130
C	-5.4067630	0.4623360	-0.5661160
H	-3.4558540	1.3417820	-0.5714820
C	-6.1772620	-0.7039910	-0.5683280
H	-6.1728530	-2.8611680	-0.5679750
H	-5.9033560	1.4222710	-0.6082310
H	-7.2554120	-0.6276950	-0.6012210
C	-1.5213700	1.4877160	1.3351870
N	-1.0286380	1.3653190	0.1052960
N	-2.0429110	2.7080750	1.5358040

C	-1.2241840	2.5691620	-0.5379340
C	-1.8739350	3.4263270	0.3598840
C	-2.6975990	3.2076330	2.7285330
C	-0.9041200	2.9807560	-1.8241750
C	-2.2259460	4.7223210	0.0144340
H	-2.2138960	4.1251120	3.0544320
H	-3.7457980	3.4090250	2.5178490
H	-2.6318480	2.4706570	3.5204390
C	-1.2521710	4.2731470	-2.1694780
H	-0.4112800	2.3043260	-2.5094490
C	-1.8994360	5.1271720	-1.2673200
H	-2.7287500	5.3815870	0.7081010
H	-1.0225190	4.6360070	-3.1620770
H	-2.1533250	6.1301530	-1.5820970
C	1.8775960	0.4722750	1.4074260
N	1.5393050	0.4046160	0.1260840
N	3.0656000	1.0703330	1.5796740
C	2.5308500	1.0598600	-0.5854270
C	3.5126010	1.4620840	0.3266930
C	3.7789530	1.2758120	2.8228420
C	2.6573710	1.3452460	-1.9383710
C	4.6642030	2.1247360	-0.0659660
H	4.7081010	0.7088140	2.8121800
H	4.0051230	2.3320320	2.9485050
H	3.1718800	0.9434160	3.6572890
C	3.7968360	2.0220370	-2.3335660
H	1.8916140	1.0327420	-2.6343570
C	4.7859700	2.3980560	-1.4170880
H	5.4234100	2.4201400	0.6450330
H	3.9309600	2.2648700	-3.3791290
H	5.6638690	2.9207330	-1.7720050

84

Mn_IV_BnTBEN_Oxo_G2_Sextet.log

84

Final Energy = -3058.9750825900

C	-1.2453800	0.4769600	2.4708660
H	-2.1935510	-0.0640880	2.4048340
H	-1.0763950	0.7200530	3.5236660
N	-0.1663460	-0.4237730	1.9564870
C	-0.3661280	-1.8089520	2.4804890
H	-0.1370940	-1.8449200	3.5516250
H	-1.4246510	-2.0504690	2.3653590
C	0.5057900	-2.7977090	1.7290860

H	1.5525810	-2.5073780	1.8155060
H	0.4082540	-3.7900690	2.1916130
N	0.1696840	-2.8375610	0.2896860
C	1.1882640	0.1095240	2.3417430
H	1.7320300	-0.6453590	2.9155330
H	1.0659150	0.9718580	3.0039460
C	-1.1121680	-3.5489700	0.0680180
H	-1.3576930	-4.1888770	0.9248790
H	-1.0097490	-4.2179940	-0.7924860
C	1.2271510	-3.5560250	-0.5070560
H	0.8908320	-3.5214520	-1.5451610
H	1.2411790	-4.6082970	-0.1880260
C	2.6187750	-2.9826750	-0.4160500
C	3.5054660	-3.3783230	0.5979160
C	3.0671970	-2.0801390	-1.3867990
C	4.7941740	-2.8450940	0.6657580
H	3.1910580	-4.1134620	1.3345250
C	4.3585940	-1.5526540	-1.3301250
H	2.3939330	-1.7813510	-2.1847110
C	5.2212760	-1.9245590	-0.2967220
H	5.4652840	-3.1546460	1.4620000
H	4.6829640	-0.8412280	-2.0834280
H	6.2229390	-1.5069450	-0.2463480
Mn	-0.2963890	-0.4589110	-0.2585640
O	-0.1846220	-0.6536570	-2.0929420
C	-2.2411040	-2.6148430	-0.2200740
N	-2.0838660	-1.2965500	-0.4234690
N	-3.5283380	-2.9826720	-0.3539100
C	-3.3492000	-0.7779560	-0.7382500
C	-4.2684570	-1.8428920	-0.6867740
C	-4.0894240	-4.3252450	-0.2188690
C	-3.7730620	0.5097760	-1.0740190
C	-5.6278260	-1.6758100	-0.9429820
H	-4.8912910	-4.3073340	0.5222180
H	-4.4834960	-4.6530840	-1.1837640
H	-3.3145290	-5.0164580	0.1072510
C	-5.1319070	0.6865890	-1.3337650
H	-3.0773280	1.3341830	-1.1420280
C	-6.0449570	-0.3837840	-1.2661910
H	-6.3270170	-2.5037790	-0.8987880
H	-5.4928090	1.6752960	-1.5995560
H	-7.0943380	-0.2028450	-1.4763160
C	-1.2988870	1.6840070	1.5908380

N	-1.0561070	1.5478190	0.2953900
N	-1.5566010	2.9728820	1.9382190
C	-1.1365900	2.8173580	-0.2591060
C	-1.4661150	3.7290510	0.7685020
C	-1.8866110	3.5125700	3.2535040
C	-0.9532920	3.2527760	-1.5754930
C	-1.6308710	5.0943420	0.5261420
H	-1.1709560	4.2951220	3.5165780
H	-2.8972290	3.9284280	3.2383730
H	-1.8397270	2.7197120	3.9981950
C	-1.1137470	4.6144430	-1.8228060
H	-0.6950630	2.5519030	-2.3628550
C	-1.4473570	5.5184160	-0.7900430
H	-1.8860360	5.7926340	1.3162540
H	-0.9791430	4.9923430	-2.8317660
H	-1.5635840	6.5717060	-1.0264300
C	1.9658880	0.5214900	1.1330020
N	1.4939540	0.3564820	-0.1047540
N	3.1474120	1.1744840	1.1265080
C	2.3864760	0.9754260	-0.9754320
C	3.4483130	1.4837880	-0.2031610
C	3.9885880	1.5029680	2.2740530
C	2.3647100	1.1260770	-2.3628350
C	4.5364120	2.1399840	-0.7780280
H	4.9640740	1.0257340	2.1550780
H	4.1108440	2.5864530	2.3406570
H	3.5208810	1.1356580	3.1863390
C	3.4444960	1.7873740	-2.9434750
H	1.5425200	0.7353540	-2.9534310
C	4.5134350	2.2818980	-2.1652690
H	5.3584050	2.5208890	-0.1816900
H	3.4668340	1.9246000	-4.0200780
H	5.3377890	2.7872580	-2.6585990

84
Mn_IV_BnTBEN_Oxo_G3_Doublet.log
84
Final Energy = -3059.2778836000

C	1.6269830	0.3631600	2.2240030
H	1.7511130	1.4433160	2.1164880
H	1.8952040	0.1130740	3.2526220
N	0.2266240	0.0129050	1.9054060
C	-0.7624440	1.0200380	2.3375740
H	-0.9748840	0.9460360	3.4064560

H	-0.3397310	2.0090390	2.1651150
C	-2.0197030	0.8081580	1.5396940
H	-2.4011760	-0.2005600	1.7024190
H	-2.8060090	1.4981050	1.8489720
N	-1.7650030	0.9435340	0.0721660
C	-0.1032380	-1.3469110	2.4163060
H	-0.8265800	-1.2730680	3.2324520
H	0.7906310	-1.7960520	2.8545740
C	-1.6704960	2.3793490	-0.3212620
H	-2.3337250	2.9930300	0.2907110
H	-2.0215640	2.4689400	-1.3513900
C	-2.8811900	0.2769130	-0.7073540
H	-2.8551430	-0.7733090	-0.4251860
H	-2.5925090	0.3466100	-1.7551240
C	-4.2430250	0.8583350	-0.4716900
C	-4.7317670	1.8825260	-1.2813090
C	-5.0549350	0.3697960	0.5514470
C	-5.9890510	2.4215680	-1.0590730
H	-4.1319190	2.2483160	-2.1065560
C	-6.3120030	0.9071610	0.7755710
H	-4.7049120	-0.4497930	1.1683780
C	-6.7790740	1.9387230	-0.0261810
H	-6.3553830	3.2126060	-1.7000280
H	-6.9310460	0.5139500	1.5711780
H	-7.7624210	2.3566220	0.1453220
Mn	0.0878740	0.0220500	-0.3762330
O	-0.0493920	-0.1019970	-1.9593760
C	-0.2654840	2.8283990	-0.2748520
N	0.7210230	1.9491610	-0.3623580
N	0.1730250	4.0970710	-0.2398520
C	1.8950840	2.6871170	-0.4119720
C	1.5542220	4.0453500	-0.3222050
C	-0.6143370	5.3105610	-0.1630380
C	3.2285760	2.3133070	-0.5502010
C	2.4995820	5.0582440	-0.3382690
H	-0.3355490	5.8771040	0.7225050
H	-0.4421460	5.9206540	-1.0469650
H	-1.6682540	5.0631290	-0.1081800
C	4.1763630	3.3208780	-0.5656360
H	3.5219470	1.2816280	-0.6771750
C	3.8214820	4.6690690	-0.4550410
H	2.2178250	6.0998590	-0.2701110
H	5.2196660	3.0584310	-0.6776000

H	4.5958550	5.4236730	-0.4734140
C	2.4525730	-0.3477950	1.2181020
N	1.9211800	-0.6212290	0.0353700
N	3.7226410	-0.7836180	1.3080620
C	2.8830570	-1.2795490	-0.7004200
C	4.0337630	-1.3774910	0.0958250
C	4.6255110	-0.6481720	2.4322540
C	2.8543590	-1.7942100	-1.9897170
C	5.1941440	-1.9825630	-0.3639410
H	5.0527220	-1.6174380	2.6772690
H	5.4272310	0.0461670	2.1877130
H	4.0841040	-0.2738630	3.2935740
C	4.0090770	-2.3998520	-2.4475550
H	1.9604440	-1.7184220	-2.5952900
C	5.1572170	-2.4908980	-1.6496770
H	6.0819660	-2.0519170	0.2491440
H	4.0299590	-2.8143920	-3.4462160
H	6.0392630	-2.9715760	-2.0503730
C	-0.6078660	-2.2148850	1.3255860
N	-0.6289200	-1.8121830	0.0678050
N	-1.0524860	-3.4800600	1.4648410
C	-1.1059700	-2.8753170	-0.6789970
C	-1.3803740	-3.9329720	0.1992240
C	-1.1744180	-4.2467820	2.6871660
C	-1.3291380	-3.0095910	-2.0444360
C	-1.8807460	-5.1491590	-0.2395490
H	-2.2170390	-4.4999680	2.8686550
H	-0.5938900	-5.1632810	2.6078240
H	-0.8017250	-3.6695420	3.5259520
C	-1.8260700	-4.2226040	-2.4856030
H	-1.1148440	-2.1942640	-2.7210080
C	-2.0972040	-5.2728330	-1.6005480
H	-2.0909320	-5.9597670	0.4444770
H	-2.0102140	-4.3655090	-3.5418870
H	-2.4853950	-6.2043020	-1.9898930

84
Mn_IV_BnTBEN_Oxo_G3_Quartet.log
84
Final Energy = -3058.8560062300

C	-1.7501040	-0.0461870	1.9520750
H	-2.2122320	-1.0293310	1.7818570
H	-2.0017540	0.2542690	2.9791450
N	-0.2843150	-0.1660310	1.7593690

C	0.3202400	-1.4330710	2.2479650
H	0.4683950	-1.4061410	3.3377900
H	-0.3663400	-2.2623420	2.0401430
C	1.6472580	-1.6009490	1.5354950
H	2.3172830	-0.7581060	1.7489990
H	2.1637560	-2.5141580	1.8626910
N	1.4316480	-1.6493510	0.0482480
C	0.4216050	1.0080270	2.3562430
H	1.0980080	0.6749790	3.1555280
H	-0.3162310	1.6600460	2.8453450
C	0.8725220	-2.9842820	-0.3348670
H	1.2642280	-3.7601430	0.3395990
H	1.2260940	-3.2303590	-1.3407470
C	2.5856620	-0.9546010	-0.6491940
H	2.7518120	0.0047080	-0.1373180
H	2.2605110	-0.7411440	-1.6753910
C	3.8318680	-1.7889280	-0.6298240
C	4.2369160	-2.5475020	-1.7171860
C	4.6762170	-1.7359890	0.5287900
C	5.4831670	-3.2051440	-1.7012520
H	3.5968810	-2.6356020	-2.5972740
C	5.9405980	-2.4044490	0.5285370
H	4.3845860	-1.2120100	1.4356930
C	6.3408600	-3.1144330	-0.5836370
H	5.7913340	-3.7843970	-2.5736380
H	6.5608890	-2.3563050	1.4247000
H	7.3013570	-3.6317430	-0.5941390
Mn	-0.0079960	-0.1977310	-0.3741130
O	0.2648010	-0.0782970	-2.0279640
C	-0.6654780	-3.0078710	-0.4096940
N	-1.4038110	-2.0943760	-0.9879210
N	-1.5255510	-4.0639940	0.1600200
C	-2.7885300	-2.5580010	-0.7958870
C	-2.7840140	-3.7392570	-0.1029520
C	-1.0698470	-5.2579680	0.8864230
C	-3.9739630	-1.9602390	-1.2147470
C	-3.9568410	-4.4123240	0.2241080
H	-1.0093870	-5.0380030	1.9318220
H	-1.7637040	-6.0572770	0.7296770
H	-0.1047490	-5.5477080	0.5265110
C	-5.1860740	-2.6118020	-0.9025590
H	-3.9662410	-1.0370530	-1.7556420
C	-5.1776440	-3.8363030	-0.1840440

H	-3.9334200	-5.3345280	0.7662250
H	-6.1162940	-2.1808210	-1.2089050
H	-6.1011670	-4.3239940	0.0486870
C	-2.2895730	0.9639950	0.9224610
N	-2.0076350	0.9571330	-0.3559830
N	-3.2012540	2.0835210	1.2309980
C	-2.7520890	2.1018890	-0.9078810
C	-3.4414490	2.7271400	0.0968030
C	-3.7438410	2.4228690	2.5543810
C	-2.8076340	2.5529450	-2.2237380
C	-4.2359820	3.8458280	-0.1329020
H	-3.0820170	3.1040250	3.0472450
H	-4.7047540	2.8793320	2.4395400
H	-3.8396970	1.5322580	3.1396360
C	-3.6030150	3.6856820	-2.4984850
H	-2.2650310	2.0569990	-3.0012460
C	-4.3163340	4.3313880	-1.4544640
H	-4.7673650	4.3210530	0.6650280
H	-3.6688790	4.0598850	-3.4987520
H	-4.9172320	5.1897830	-1.6712110
C	1.1805910	1.8536340	1.3167770
N	1.0663510	1.7289160	0.0185670
N	2.1447460	2.9249470	1.6367640
C	1.9786260	2.7440920	-0.5350060
C	2.5877380	3.4194540	0.4889150
C	2.5413650	3.3635100	2.9825930
C	2.2448690	3.0424260	-1.8684710
C	3.5055140	4.4401730	0.2623510
H	3.3876210	2.7952700	3.3079450
H	2.7971880	4.4021940	2.9583020
H	1.7279810	3.2122920	3.6611510
C	3.1705930	4.0720590	-2.1406880
H	1.7633710	2.5085220	-2.6609390
C	3.8001800	4.7701190	-1.0767000
H	3.9713840	4.9566640	1.0754320
H	3.3992280	4.3280190	-3.1541520
H	4.5009740	5.5495920	-1.2916620

84

Mn_IV_BnTBEN_Oxo_G3_Sextet.log

84

Final Energy = -3058.9753182700

C	-1.6324240	-0.1061500	2.4141920
H	-1.6714860	-1.1988100	2.4360050

H	-1.8097430	0.2528450	3.4329710
N	-0.2785210	0.3041940	1.9381360
C	0.7808350	-0.5415770	2.5664620
H	0.9106710	-0.2645250	3.6184770
H	0.4280920	-1.5748750	2.5429530
C	2.0976480	-0.4140240	1.8156030
H	2.4260060	0.6277050	1.7958070
H	2.8701350	-0.9830770	2.3473730
N	1.9413360	-0.8670100	0.4168580
C	-0.0621220	1.7630630	2.2301030
H	0.5508140	1.8767320	3.1289290
H	-1.0294100	2.2294550	2.4398850
C	1.8409150	-2.3378440	0.3175460
H	2.1499150	-2.8166500	1.2541560
H	2.5293620	-2.7093220	-0.4462250
C	2.9844640	-0.3095170	-0.5017120
H	2.9512640	0.7746360	-0.3827120
H	2.6516230	-0.5332940	-1.5175670
C	4.3874640	-0.8256490	-0.2736370
C	4.9056990	-1.8473870	-1.0825350
C	5.1951670	-0.2997460	0.7462870
C	6.1905240	-2.3519970	-0.8635630
H	4.3081170	-2.2407700	-1.9018990
C	6.4787190	-0.8018990	0.9695900
H	4.8274500	0.5170130	1.3613250
C	6.9776330	-1.8342760	0.1683370
H	6.5763630	-3.1418810	-1.5018570
H	7.0918200	-0.3821860	1.7622230
H	7.9775000	-2.2228940	0.3397360
Mn	-0.1924090	0.0437330	-0.2466150
O	0.0284730	-0.1004970	-2.0710260
C	0.4529940	-2.7694830	-0.0406060
N	-0.5479350	-1.9111590	-0.3061210
N	0.0444000	-4.0467600	-0.1459370
C	-1.6741250	-2.6833740	-0.6227080
C	-1.3064590	-4.0377640	-0.5107230
C	0.8364670	-5.2547990	0.0759880
C	-2.9702100	-2.3285000	-1.0028970
C	-2.1997490	-5.0806010	-0.7457080
H	0.4564160	-5.7869680	0.9512390
H	0.7660190	-5.8948950	-0.8058630
H	1.8784200	-4.9847800	0.2382160
C	-3.8714560	-3.3657150	-1.2421180

H	-3.2625510	-1.2944820	-1.1244820
C	-3.4965790	-4.7172780	-1.1126480
H	-1.9064230	-6.1203950	-0.6507100
H	-4.8865930	-3.1230250	-1.5404550
H	-4.2291820	-5.4940360	-1.3075770
C	-2.6317560	0.4194000	1.4317570
N	-2.2557020	0.6688810	0.1834740
N	-3.9478260	0.6784500	1.6388960
C	-3.3843690	1.1279670	-0.4843570
C	-4.4657100	1.1319900	0.4239540
C	-4.7230510	0.4967580	2.8621710
C	-3.5602190	1.5243970	-1.8137470
C	-5.7505030	1.5297600	0.0492820
H	-5.2063890	1.4388150	3.1309360
H	-5.4811980	-0.2750020	2.7067110
H	-4.0619200	0.1898380	3.6713700
C	-4.8402270	1.9224710	-2.1939770
H	-2.7296200	1.5120140	-2.5123150
C	-5.9152600	1.9259060	-1.2780820
H	-6.5805190	1.5298930	0.7480040
H	-5.0186500	2.2360770	-3.2180400
H	-6.8969740	2.2430080	-1.6164550
C	0.5510500	2.4485050	1.0533850
N	0.5543410	1.8813210	-0.1568720
N	1.1133280	3.6751100	1.0278070
C	1.1516740	2.7840570	-1.0339760
C	1.5097950	3.9257710	-0.2899610
C	1.2942260	4.6015250	2.1438690
C	1.4175040	2.6989190	-2.4025690
C	2.1387970	5.0254150	-0.8751380
H	2.3608230	4.7688120	2.3108510
H	0.8025490	5.5482630	1.9098950
H	0.8475610	4.1822730	3.0440680
C	2.0458060	3.7936550	-2.9921830
H	1.1448830	1.8143840	-2.9688580
C	2.3991430	4.9362760	-2.2422130
H	2.4125750	5.9038650	-0.3007910
H	2.2700170	3.7689590	-4.0540470
H	2.8869160	5.7672110	-2.7421700

63
Mn_IV_BnTPEN_Oxo_G1_Doublet.log
63
Final Energy = -2546.4399665600

C	3.1565640	0.4743990	-0.1783280
C	2.4082740	-0.2990230	-2.2418660
C	3.6805110	-0.2298420	-2.7676450
C	4.7190800	0.2082210	-1.9625740
C	4.4527090	0.5564580	-0.6501270
H	1.5618660	-0.6348690	-2.8244850
H	3.8447440	-0.5214580	-3.7943140
H	5.7270250	0.2675700	-2.3489080
H	5.2373470	0.8862460	0.0158090
C	2.7468410	0.8464540	1.2084010
H	2.4603360	1.8996950	1.2458750
H	3.5660170	0.7184910	1.9190640
N	1.5694560	0.0322020	1.5654480
C	0.7489560	0.6191890	2.6444340
H	1.1993030	0.4536680	3.6254800
H	0.7009760	1.6971200	2.4954020
C	-0.6079590	-0.0143630	2.5702500
H	-0.5325670	-1.0940950	2.7090290
H	-1.2727430	0.3593610	3.3506670
N	-1.2380630	0.2124670	1.2298670
C	1.9773400	-1.3627160	1.8882180
H	1.8842360	-1.5399140	2.9612400
H	3.0405740	-1.4724970	1.6672280
C	1.2401930	-2.4038390	1.1106730
C	1.3744860	-3.7509000	1.4095350
C	0.7711220	-4.6888710	0.5954010
H	1.9603220	-4.0475670	2.2684320
C	-0.0476490	-2.9043620	-0.7461440
C	0.0622700	-4.2569070	-0.5152550
H	0.8641770	-5.7434450	0.8151740
H	-0.5771300	-2.5050430	-1.6000230
H	-0.4035200	-4.9537480	-1.1961230
C	-1.7580990	1.6037130	1.1309640
H	-2.1679530	1.9272410	2.0902050
H	-2.5892630	1.5917780	0.4246280
C	-2.3849020	-0.7951940	1.1447100
H	-2.8712790	-0.7677100	2.1228540
H	-1.9094980	-1.7699910	1.0486120
C	-0.7669480	2.5738700	0.5967740
C	-0.8948230	3.9392080	0.7952700
C	1.0231690	2.9009920	-0.8330140
C	-0.0281970	4.7964070	0.1453920
H	-1.6744230	4.3122080	1.4442170

C	0.9376200	4.2686450	-0.6977630
H	1.7654520	2.4497250	-1.4760020
H	-0.1100120	5.8653020	0.2869590
H	1.6227870	4.9022090	-1.2407490
C	-3.4136080	-0.5829780	0.0820980
C	-4.5453800	0.1894940	0.3544460
C	-3.3150980	-1.2004670	-1.1623530
C	-5.5300870	0.3682110	-0.6018860
H	-4.6551200	0.6464050	1.3314360
C	-4.2996200	-1.0237790	-2.1219880
H	-2.4702280	-1.8387340	-1.3747600
C	-5.4051480	-0.2341160	-1.8464190
H	-6.3996360	0.9697990	-0.3727250
H	-4.2067740	-1.5137060	-3.0822000
H	-6.1758860	-0.0990520	-2.5939510
N	2.1496750	0.0567130	-0.9729300
N	0.5063670	-1.9893090	0.0666090
N	0.1995490	2.0630110	-0.1833580
Mn	0.2814800	0.0174050	-0.2202300
O	-0.5718370	-0.1366380	-1.5573780

63
Mn_IV_BnTPEN_Oxo_G1_Quartet.log
63
Final Energy = -2546.2408819800

C	-3.2548250	0.3508710	0.0982100
C	-2.5216380	-0.2708170	2.2408170
C	-3.8259840	-0.2985110	2.7170510
C	-4.8687370	0.0087870	1.8417890
C	-4.5792450	0.3353470	0.5151080
H	-1.6598340	-0.4939290	2.8582160
H	-4.0136320	-0.5587230	3.7523410
H	-5.8979580	-0.0095910	2.1848530
H	-5.3677290	0.5727520	-0.1902430
C	-2.8152500	0.7360900	-1.2927750
H	-2.5943730	1.8064060	-1.3270650
H	-3.5999760	0.5438060	-2.0309960
N	-1.5714100	-0.0105710	-1.6041200
C	-0.7103450	0.6363170	-2.6267530
H	-1.0938970	0.4630290	-3.6384500
H	-0.7177750	1.7120110	-2.4523890
C	0.6865510	0.0565120	-2.4987170
H	0.6641660	-1.0133040	-2.7181040
H	1.3661380	0.5216200	-3.2193070

N	1.2581300	0.2046300	-1.1078030
C	-1.8947790	-1.4260100	-1.9597280
H	-1.7113510	-1.5981080	-3.0244810
H	-2.9648500	-1.5917110	-1.8023810
C	-1.1505720	-2.4263680	-1.1183720
C	-1.1825130	-3.7900730	-1.4016500
C	-0.5624290	-4.6791170	-0.5276700
H	-1.6967160	-4.1422860	-2.2894430
C	0.0752500	-2.8166870	0.8356200
C	0.0655150	-4.1844820	0.6192220
H	-0.5765090	-5.7445890	-0.7325320
H	0.5419950	-2.3578190	1.6972420
H	0.5450170	-4.8442090	1.3330020
C	1.8079660	1.5760780	-0.8991440
H	2.3671190	1.9039120	-1.7808140
H	2.5124320	1.5183170	-0.0659670
C	2.3812370	-0.8360020	-1.0131850
H	2.8190350	-0.8983500	-2.0136160
H	1.8932500	-1.7878900	-0.8089160
C	0.7565080	2.5662160	-0.5080890
C	0.9176440	3.9358340	-0.7005970
C	-1.2117010	2.9149960	0.6930220
C	-0.0245100	4.8110880	-0.1658460
H	1.7764350	4.3010190	-1.2528020
C	-1.0995770	4.2906590	0.5573590
H	-2.0313950	2.4715260	1.2411530
H	0.0818980	5.8819580	-0.3038150
H	-1.8476140	4.9336000	1.0063860
C	3.4921810	-0.5645140	-0.0317570
C	4.7061540	-0.0465690	-0.5101700
C	3.3741480	-0.8589850	1.3333390
C	5.7751650	0.1867670	0.3569550
H	4.8139990	0.1764740	-1.5689050
C	4.4417860	-0.6268740	2.2026960
H	2.4391760	-1.2420700	1.7202270
C	5.6438270	-0.1022020	1.7183100
H	6.7071260	0.5886230	-0.0301730
H	4.3343200	-0.8582080	3.2586900
H	6.4739050	0.0757580	2.3962020
N	-2.2511440	0.0491010	0.9612030
N	-0.5058460	-1.9568670	-0.0302110
N	-0.3121490	2.0678570	0.1513300
Mn	-0.3899080	0.0334170	0.2058380

O 0.4164090 -0.0643230 1.6516340
63
Mn_IV_BnTPEN_Oxo_G1_Sextet.log
63
Final Energy = -2546.4156554700

C	2.7906210	1.1844890	-0.1433280
C	2.4836180	0.1502620	-2.1846520
C	3.8300240	0.2443650	-2.4849940
C	4.6750880	0.8308520	-1.5578010
C	4.1503030	1.3071140	-0.3666930
H	1.7804880	-0.3114780	-2.8685480
H	4.2029250	-0.1405650	-3.4228720
H	5.7352690	0.9120560	-1.7558550
H	4.7827210	1.7653630	0.3809460
C	2.1108290	1.7182740	1.0791460
H	1.6091650	2.6549440	0.8252390
H	2.8279050	1.9462670	1.8698900
N	1.0675190	0.7850210	1.5836410
C	0.1148260	1.5023050	2.4770390
H	0.5900140	1.7271620	3.4349260
H	-0.1121180	2.4580740	2.0077270
C	-1.1363460	0.6896220	2.6989010
H	-0.9070480	-0.2120060	3.2655930
H	-1.8416540	1.2612840	3.3082550
N	-1.7638210	0.2592230	1.4241620
C	1.6999240	-0.3672760	2.2915340
H	1.0077540	-0.7171980	3.0583520
H	2.5971150	-0.0359730	2.8171520
C	1.9936380	-1.5094110	1.3763070
C	3.0475650	-2.3824430	1.5809730
C	3.2145480	-3.4484150	0.7136210
H	3.7256560	-2.2196790	2.4066480
C	1.3103880	-2.6967010	-0.4950030
C	2.3308540	-3.6113610	-0.3415820
H	4.0341300	-4.1398710	0.8541640
H	0.6088290	-2.7417270	-1.3196240
H	2.4345020	-4.4237180	-1.0453880
C	-2.6310490	1.3101050	0.8648460
H	-3.2472850	1.7620300	1.6477340
H	-3.3196170	0.8299900	0.1654460
C	-2.5707740	-0.9758970	1.6635830
H	-3.4179070	-0.7339890	2.3139010
H	-1.9260020	-1.6656070	2.2087090

C	-1.9081110	2.3644220	0.0924170
C	-2.4352830	3.6386250	-0.0447500
C	-0.1624440	2.8886950	-1.3297270
C	-1.7957890	4.5507000	-0.8625180
H	-3.3404390	3.8981340	0.4865420
C	-0.6416340	4.1661780	-1.5259930
H	0.7404880	2.5463100	-1.8165230
H	-2.1928980	5.5496550	-0.9810640
H	-0.1117530	4.8426580	-2.1799690
C	-3.0407930	-1.6167000	0.3969040
C	-4.3273390	-1.4422430	-0.0801980
C	-2.1534820	-2.4223010	-0.3412670
C	-4.7267470	-2.0391790	-1.2741170
H	-5.0330260	-0.8444850	0.4837980
C	-2.5624230	-3.0310680	-1.5355430
H	-1.2071600	-2.6972620	0.1034450
C	-3.8422950	-2.8290160	-2.0042080
H	-5.7364440	-1.8887870	-1.6327760
H	-1.8680630	-3.6592160	-2.0768420
H	-4.1658000	-3.2922070	-2.9261130
N	1.9735530	0.6143650	-1.0424350
N	1.1386170	-1.6750660	0.3528090
N	-0.7759090	2.0039540	-0.5299230
Mn	-0.0333190	0.0278290	-0.1056600
O	-0.9753470	-0.7602950	-1.4333200

63
Mn_IV_BnTPEN_Oxo_G2_Doublet.log
63
Final Energy = -2546.4382702700

C	-2.2525600	1.9064570	0.5534750
C	-1.4784120	2.3657430	-1.5941760
C	-2.2636030	3.4960960	-1.6723550
C	-3.0678370	3.8327540	-0.5958550
C	-3.0529360	3.0326000	0.5329680
H	-0.8334920	2.0567920	-2.4050940
H	-2.2349240	4.0994050	-2.5673910
H	-3.6904540	4.7158700	-0.6297520
H	-3.6490570	3.2730910	1.4018050
C	-2.1638870	0.9659660	1.7102180
H	-2.8960990	0.1625140	1.6034880
H	-2.3846230	1.4702790	2.6531440
N	-0.8130470	0.3731390	1.6999080
C	-0.7026850	-0.8881760	2.4601420

H	-0.6035520	-0.7032580	3.5312510
H	-1.6208740	-1.4587980	2.3199000
C	0.4908250	-1.6199820	1.9165880
H	1.3802180	-0.9948580	1.9863470
H	0.6924150	-2.5433770	2.4637800
N	0.2619390	-1.9251870	0.4752150
C	0.1958190	1.3644490	2.1670610
H	0.5988820	1.0528860	3.1325590
H	-0.3078400	2.3134270	2.3593800
C	1.3000150	1.6114280	1.1929820
C	2.3540050	2.4525590	1.5142730
C	3.3006730	2.7472910	0.5535730
H	2.4085520	2.8773460	2.5071110
C	2.1014150	1.3577850	-0.9608840
C	3.1603970	2.2022840	-0.7131530
H	4.1290440	3.4028550	0.7847270
H	1.9425140	0.8993080	-1.9263970
H	3.8646040	2.4169740	-1.5035890
C	-0.8160210	-2.9653870	0.3655000
H	-0.8713290	-3.5393450	1.2919220
H	-0.5179580	-3.6765550	-0.4051140
C	1.4954950	-2.5668370	-0.1372000
H	1.1718450	-2.9191760	-1.1134130
H	1.6982370	-3.4493110	0.4789930
C	-2.1465240	-2.4314140	-0.0227410
C	-3.2872550	-3.2159300	0.0524750
C	-3.3262650	-0.7305870	-1.0530220
C	-4.4749490	-2.7239400	-0.4500350
H	-3.2261460	-4.2025070	0.4897680
C	-4.4897050	-1.4651590	-1.0324910
H	-3.2972170	0.2542630	-1.4960120
H	-5.3766200	-3.3186890	-0.4009940
H	-5.3894980	-1.0505360	-1.4617120
C	2.7467050	-1.7693780	-0.3190330
C	3.5910570	-1.4178340	0.7353850
C	3.1672970	-1.5068170	-1.6230240
C	4.7978300	-0.7820310	0.4899830
H	3.3308190	-1.6730910	1.7548810
C	4.3865020	-0.8967750	-1.8710420
H	2.5294800	-1.7959750	-2.4499860
C	5.2006230	-0.5237120	-0.8129970
H	5.4379350	-0.5117590	1.3196130
H	4.6976190	-0.7109730	-2.8907330

H	6.1516670	-0.0426530	-1.0010160
N	-1.4836590	1.5791780	-0.5056760
N	1.1982690	1.0451180	-0.0181080
N	-2.1749560	-1.1936590	-0.5362620
Mn	-0.4262340	-0.1339270	-0.4199960
O	0.0007180	-0.3993500	-1.9315960

63

Mn_IV_BnTPEN_Oxo_G2_Quartet.log

63

Final Energy = -2546.4592051400

C	-1.8880740	1.9998530	0.9495020
C	-1.8028240	2.4873490	-1.3341470
C	-2.3999640	3.7135000	-1.1317430
C	-2.7381170	4.0851250	0.1596510
C	-2.4777840	3.2220160	1.2112170
H	-1.5003070	2.1286560	-2.3095550
H	-2.5873690	4.3622240	-1.9741730
H	-3.1986960	5.0449260	0.3492600
H	-2.7279060	3.4857840	2.2289370
C	-1.6461850	0.9577080	1.9985130
H	-2.5244480	0.3100040	2.0553750
H	-1.5341810	1.4111710	2.9847120
N	-0.4845620	0.1135890	1.6476560
C	-0.5901360	-1.2799600	2.1454020
H	-0.3634470	-1.3415930	3.2125010
H	-1.6221990	-1.6018240	2.0248390
C	0.3746030	-2.1502760	1.3768740
H	1.3902680	-1.8595490	1.6284120
H	0.2656290	-3.1974380	1.6653470
N	0.2461830	-2.0132170	-0.1100030
C	0.7984860	0.7516940	2.0445840
H	1.4639050	-0.0064570	2.4603230
H	0.6287230	1.4623860	2.8548820
C	1.4842950	1.4281260	0.9023390
C	2.5018650	2.3483800	1.0942180
C	3.0680340	2.9665760	-0.0046400
H	2.8231150	2.5834130	2.0993400
C	1.6072040	1.7169730	-1.4035940
C	2.6033610	2.6571210	-1.2746390
H	3.8558810	3.6953410	0.1289740
H	1.1969980	1.4105780	-2.3574910
H	3.0080490	3.1313820	-2.1563180
C	-0.7954460	-2.9324760	-0.6487900

H -0.6421610 -3.9392210 -0.2554520
H -0.6421330 -2.9799020 -1.7293920
C 1.5235660 -2.4501160 -0.8087230
H 1.3220200 -2.3001180 -1.8681530
H 1.6021560 -3.5272190 -0.6322970
C -2.1795240 -2.4553390 -0.4126830
C -3.2564260 -3.3158440 -0.3001700
C -3.5784210 -0.6124850 -0.3140120
C -4.5310480 -2.7907410 -0.1984050
H -3.0844860 -4.3825410 -0.2982260
C -4.6940870 -1.4157390 -0.2154930
H -3.6723790 0.4632270 -0.3289300
H -5.3866390 -3.4456920 -0.1091710
H -5.6702500 -0.9594590 -0.1486070
C 2.8080460 -1.7820670 -0.4352940
C 3.5465780 -2.1774890 0.6823320
C 3.3535460 -0.8213450 -1.2827700
C 4.7595810 -1.5772390 0.9787150
H 3.1818570 -2.9790880 1.3135400
C 4.5708260 -0.2251640 -0.9947260
H 2.8170440 -0.5527850 -2.1853530
C 5.2695500 -0.5915660 0.1458730
H 5.3153270 -1.8929700 1.8516630
H 4.9761670 0.5210540 -1.6659470
H 6.2200540 -0.1269080 0.3732070
N -1.5618460 1.6553250 -0.3132610
N 1.0746500 1.1045360 -0.3340080
N -2.3396990 -1.1203040 -0.4002480
Mn -0.5595410 -0.0839830 -0.5135370
O -0.4517710 -0.1920930 -2.1726360
63
Mn_IV_BnTPEN_Oxo_G2_Sextet.log
63
Final Energy = -2546.4114470400
C -1.8140470 2.1493120 0.9692810
C -1.6895750 2.7560100 -1.2530450
C -2.0399450 4.0612180 -0.9599830
C -2.2756280 4.4036720 0.3611740
C -2.1592100 3.4343950 1.3452320
H -1.4826320 2.4367220 -2.2684380
H -2.1189090 4.7903900 -1.7529780
H -2.5410560 5.4182000 0.6258550
H -2.3293040 3.6673280 2.3871740

C	-1.6951520	1.0097320	1.9291460
H	-2.5707970	0.3634360	1.8352200
H	-1.6560740	1.3514090	2.9645900
N	-0.4988000	0.1753250	1.6229410
C	-0.5967190	-1.1360160	2.3271020
H	-0.4412490	-0.9970350	3.3990690
H	-1.6194190	-1.4923020	2.1998520
C	0.3974680	-2.1184760	1.7697620
H	1.4103610	-1.7480180	1.9154260
H	0.3304360	-3.0632420	2.3184030
N	0.2057480	-2.3334010	0.3194150
C	0.7553310	0.9049820	1.9968420
H	1.4682780	0.1819770	2.3952510
H	0.5449120	1.6003610	2.8104490
C	1.3972620	1.6054720	0.8437900
C	2.2512180	2.6810460	1.0130040
C	2.8512110	3.2464020	-0.0984080
H	2.4335950	3.0673150	2.0056840
C	1.7230290	1.6593340	-1.4602630
C	2.5863420	2.7273900	-1.3562740
H	3.5158710	4.0916680	0.0160900
H	1.4659820	1.2201000	-2.4168010
H	3.0331990	3.1438050	-2.2465440
C	-0.9458210	-3.2212430	0.0677970
H	-1.0682150	-3.9462960	0.8773810
H	-0.7243490	-3.8111680	-0.8243030
C	1.4225110	-2.9815060	-0.2801740
H	1.1533730	-3.2235680	-1.3071610
H	1.5957050	-3.9275860	0.2449550
C	-2.2434410	-2.5297220	-0.2004270
C	-3.4421040	-3.2215860	-0.1196110
C	-3.3529100	-0.6527960	-1.0100260
C	-4.6131060	-2.6018280	-0.5085020
H	-3.4397090	-4.2421930	0.2369310
C	-4.5665110	-1.2972030	-0.9783510
H	-3.2609590	0.3682180	-1.3507710
H	-5.5538520	-3.1324360	-0.4534690
H	-5.4552690	-0.7802620	-1.3075270
C	2.6673790	-2.1485810	-0.2793490
C	3.5632490	-2.1773740	0.7896430
C	2.9793790	-1.3648160	-1.3889590
C	4.7185800	-1.4118850	0.7668750
H	3.3622230	-2.8203410	1.6389370

C	4.1390850	-0.6060500	-1.4188640
H	2.3025150	-1.3560140	-2.2373460
C	5.0068100	-0.6211930	-0.3368040
H	5.4015300	-1.4442380	1.6055930
H	4.3675700	-0.0063300	-2.2907620
H	5.9131800	-0.0302030	-0.3590680
N	-1.5828120	1.8193830	-0.3086940
N	1.1468490	1.1107790	-0.3822770
N	-2.2142850	-1.2512190	-0.6113720
Mn	-0.4428600	-0.1845980	-0.4712570
O	-0.3138040	-0.3753690	-2.3179990

63

Mn_IV_BnTPEN_Oxo_G3_Doublet.log

63

Final Energy = -2546.4441380600

C	3.2456660	0.1051560	0.7872270
C	3.4341950	-0.4184660	-1.4733520
C	4.8055580	-0.5125190	-1.3714330
C	5.4066670	-0.2928260	-0.1429840
C	4.6146340	0.0125410	0.9499690
H	2.9133540	-0.5868250	-2.4055240
H	5.3850330	-0.7611830	-2.2480210
H	6.4796830	-0.3678260	-0.0347060
H	5.0428920	0.1765140	1.9286020
C	2.2954790	0.4642920	1.8808230
H	2.1406260	1.5451330	1.8987180
H	2.6856520	0.1886380	2.8622130
N	1.0012820	-0.1865160	1.5954780
C	-0.1462350	0.4771790	2.2520650
H	-0.1982640	0.2274710	3.3141220
H	-0.0037570	1.5550010	2.1841890
C	-1.3947250	0.0364790	1.5438520
H	-1.5192380	-1.0425600	1.6455870
H	-2.2844720	0.5021500	1.9691590
N	-1.3086310	0.3269810	0.0792490
C	1.0642190	-1.6394090	1.9188350
H	0.4572690	-1.8501260	2.8013740
H	2.0872690	-1.8883510	2.2058780
C	0.6677710	-2.5288620	0.7840800
C	0.5019220	-3.8933890	0.9639770
C	0.2082160	-4.6887650	-0.1262580
H	0.6109600	-4.3152880	1.9534220
C	0.2496930	-2.7382660	-1.4876480

C	0.0899230	-4.1009770	-1.3767860
H	0.0756500	-5.7548370	-0.0036300
H	0.1516440	-2.2152560	-2.4295430
H	-0.1313260	-4.6853530	-2.2575500
C	-1.6370910	1.7487030	-0.2211970
H	-2.4666640	2.0906960	0.3990960
H	-1.9856780	1.7819440	-1.2557990
C	-2.2670350	-0.5737810	-0.6760270
H	-1.9918650	-1.5943740	-0.4189470
H	-2.0501090	-0.4209040	-1.7324000
C	-0.4627480	2.6565450	-0.1196170
C	-0.5992260	4.0174650	0.0931200
C	1.8234060	2.8785260	-0.4010560
C	0.5250940	4.8203060	0.0483910
H	-1.5790990	4.4315840	0.2833800
C	1.7567460	4.2422860	-0.2149160
H	2.7652840	2.3885540	-0.6054750
H	0.4413410	5.8857780	0.2125430
H	2.6587400	4.8329590	-0.2719780
C	-3.7163190	-0.3292990	-0.3793220
C	-4.4681770	0.5213510	-1.1880540
C	-4.3433560	-0.9629660	0.6926990
C	-5.8077490	0.7517680	-0.9192770
H	-4.0034380	0.9933410	-2.0461730
C	-5.6822440	-0.7322650	0.9642340
H	-3.7834610	-1.6556440	1.3101330
C	-6.4154400	0.1299150	0.1616540
H	-6.3790040	1.4108950	-1.5595530
H	-6.1559140	-1.2341280	1.7976090
H	-7.4623140	0.3068740	0.3708300
N	2.6694960	-0.1060830	-0.4143520
N	0.5248320	-1.9641440	-0.4243340
N	0.7373470	2.0941100	-0.3416600
Mn	0.6717720	0.0555850	-0.5571780
O	0.4301960	0.1013410	-2.1320950

63

Mn_IV_BnTPEN_Oxo_G3_Quartet.log

63

Final Energy = -2546.4649867100

C	3.2829040	0.1057220	0.7371470
C	3.4304160	-0.3171060	-1.5566560
C	4.8040300	-0.4123820	-1.4864110
C	5.4228610	-0.2433380	-0.2582490

C	4.6552550	0.0142780	0.8659880
H	2.8737110	-0.4365220	-2.4770810
H	5.3719370	-0.6189290	-2.3811460
H	6.4982950	-0.3179500	-0.1733440
H	5.1082200	0.1414420	1.8389850
C	2.3596720	0.4345100	1.8716430
H	2.2086410	1.5154630	1.9163280
H	2.7925650	0.1435230	2.8302090
N	1.0512390	-0.2060530	1.6330970
C	-0.0966440	0.4742080	2.2760300
H	-0.1578880	0.2340130	3.3399620
H	0.0590850	1.5493530	2.2032520
C	-1.3650860	0.0422170	1.5822650
H	-1.5071130	-1.0314730	1.7145530
H	-2.2363350	0.5314330	2.0204390
N	-1.3203280	0.2890150	0.1043060
C	1.0991610	-1.6561330	1.9566040
H	0.4872570	-1.8661390	2.8357590
H	2.1184390	-1.9194930	2.2447890
C	0.6958440	-2.5341310	0.8164060
C	0.5218260	-3.8997670	0.9720810
C	0.2239330	-4.6720060	-0.1341970
H	0.6286550	-4.3409200	1.9531100
C	0.2743600	-2.7047910	-1.4694140
C	0.1086260	-4.0671900	-1.3778660
H	0.0860340	-5.7394450	-0.0290930
H	0.1817300	-2.1510580	-2.3947210
H	-0.1149580	-4.6391770	-2.2659100
C	-1.6684210	1.6906400	-0.2469710
H	-2.5345120	2.0274540	0.3249750
H	-1.9644970	1.6860700	-1.2987520
C	-2.2637650	-0.6451600	-0.6180130
H	-2.0048030	-1.6524930	-0.2969970
H	-2.0129780	-0.5498360	-1.6746450
C	-0.5222280	2.6279020	-0.1143630
C	-0.6944020	3.9870190	0.0791210
C	1.7707340	2.9017880	-0.3227990
C	0.4113660	4.8162010	0.0620400
H	-1.6895230	4.3787920	0.2334290
C	1.6638480	4.2651330	-0.1543380
H	2.7303200	2.4339350	-0.4906970
H	0.2973680	5.8808760	0.2118380
H	2.5537710	4.8754120	-0.1878250

C	-3.7184870	-0.3764450	-0.3706770
C	-4.4530870	0.4046570	-1.2607320
C	-4.3647190	-0.9132970	0.7417580
C	-5.7962840	0.6601940	-1.0349970
H	-3.9703110	0.8038150	-2.1454590
C	-5.7073030	-0.6574190	0.9701870
H	-3.8175000	-1.5512760	1.4256740
C	-6.4243570	0.1336600	0.0842750
H	-6.3542370	1.2648780	-1.7377550
H	-6.1962090	-1.0846360	1.8358110
H	-7.4739790	0.3298470	0.2598950
N	2.6946110	-0.0589400	-0.4677720
N	0.5532280	-1.9571560	-0.3876500
N	0.7000900	2.0947080	-0.2893970
Mn	0.6840560	0.0534380	-0.4938930
O	0.4106520	0.1102870	-2.1369410

63

Mn_IV_BnTPEN_Oxo_G3_Sextet.log

63

Final Energy = -2546.4131072400

C	3.4210250	-0.0187650	0.9463500
C	3.9294840	-0.4275020	-1.2725510
C	5.2721250	-0.5685370	-0.9820890
C	5.6843800	-0.4288410	0.3332210
C	4.7466300	-0.1559820	1.3154360
H	3.5500630	-0.5308750	-2.2819210
H	5.9751930	-0.7876170	-1.7720930
H	6.7279320	-0.5393590	0.5949600
H	5.0340040	-0.0525860	2.3524730
C	2.3307630	0.3250070	1.9130640
H	2.1888840	1.4077240	1.9430660
H	2.5776160	0.0124570	2.9295510
N	1.0466620	-0.2845250	1.4789910
C	-0.0985250	0.3554000	2.1848730
H	-0.0939460	0.0623490	3.2368150
H	0.0650570	1.4327190	2.1564990
C	-1.4063070	-0.0136440	1.5370830
H	-1.5620250	-1.0922760	1.6004340
H	-2.2305750	0.4498740	2.0873630
N	-1.4232420	0.3543900	0.1072390
C	1.0866800	-1.7661360	1.7013170
H	0.6167310	-2.0083150	2.6558070
H	2.1346840	-2.0581280	1.7971190

C	0.4962630	-2.5663930	0.5858770
C	0.0830780	-3.8766440	0.7567880
C	-0.3458500	-4.5956710	-0.3444510
H	0.1076760	-4.3212930	1.7414580
C	0.0432960	-2.6802060	-1.6965160
C	-0.3558570	-3.9942580	-1.5941200
H	-0.6706020	-5.6205570	-0.2284190
H	0.0300420	-2.1457340	-2.6384800
H	-0.6796080	-4.5270640	-2.4754530
C	-1.6727470	1.7878390	-0.1114890
H	-2.4005550	2.1855830	0.6015800
H	-2.1335630	1.8926340	-1.0968980
C	-2.4099370	-0.4717820	-0.6520750
H	-2.1531620	-1.5144350	-0.4643800
H	-2.2311780	-0.2732890	-1.7098360
C	-0.4485330	2.6512010	-0.1291970
C	-0.5325110	4.0115200	0.1196850
C	1.8138720	2.8615790	-0.6405430
C	0.5908660	4.8024110	-0.0296650
H	-1.4799730	4.4357240	0.4212120
C	1.7851220	4.2200700	-0.4281680
H	2.7205340	2.3530430	-0.9407650
H	0.5359740	5.8657130	0.1598000
H	2.6832550	4.8030340	-0.5656960
C	-3.8491280	-0.2257060	-0.2977310
C	-4.6176430	0.6748220	-1.0324830
C	-4.4414100	-0.8871770	0.7771700
C	-5.9374610	0.9255520	-0.6901390
H	-4.1817490	1.1723130	-1.8916190
C	-5.7601780	-0.6375090	1.1227390
H	-3.8694620	-1.6171420	1.3386870
C	-6.5095740	0.2737470	0.3923920
H	-6.5215320	1.6246180	-1.2743610
H	-6.2059730	-1.1621810	1.9577450
H	-7.5406650	0.4658690	0.6590760
N	3.0236460	-0.1525180	-0.3286320
N	0.4574760	-1.9850140	-0.6273370
N	0.7226900	2.0911660	-0.4819300
Mn	0.8172500	0.0312770	-0.6050200
O	0.5496890	0.2118020	-2.4394020

74

Mn_IV_MeTBEN_Oxo_G1_Doublet.log

74

Final Energy = -2827.7375055100

C	-0.9492270	0.2923490	1.9601330
H	-2.0061450	0.0758520	1.7476210
H	-0.8705990	0.4834270	3.0398470
N	-0.1293210	-0.8730920	1.5483820
C	-0.7429340	-2.2034590	1.7997880
H	-0.6289260	-2.4988650	2.8534160
H	-1.8194710	-2.1462190	1.5996230
C	-0.0473760	-3.1972180	0.8912650
H	1.0294550	-3.2344420	1.1009070
H	-0.4392970	-4.2136370	1.0364230
N	-0.2204400	-2.7909070	-0.5459870
C	1.2290830	-0.8045080	2.1672740
H	1.3907460	-1.6739650	2.8193560
H	1.2792440	0.0736480	2.8268760
C	-1.6130130	-3.1098170	-0.9937340
H	-1.9752850	-4.0099660	-0.4778360
H	-1.5858380	-3.3475260	-2.0637720
Mn	0.0169730	-0.7173520	-0.5925160
O	0.2795100	-0.5533910	-2.2442110
C	-2.5902330	-1.9281830	-0.8509650
N	-2.3513870	-0.7034170	-1.2470140
N	-3.9351000	-2.0088800	-0.2472120
C	-3.5698730	0.0459320	-0.8962470
C	-4.4713370	-0.7974140	-0.3032250
C	-4.5649200	-3.2128320	0.3137930
C	-3.8515590	1.3946040	-1.0956300
C	-5.7180120	-0.3620750	0.1347160
H	-4.3249230	-3.2892870	1.3537240
H	-5.6264800	-3.1475930	0.1965990
H	-4.2017980	-4.0779620	-0.2005920
C	-5.1062700	1.8758420	-0.6651440
H	-3.1400210	2.0449350	-1.5600470
C	-6.0383990	0.9987000	-0.0507360
H	-6.4093750	-1.0359750	0.5959920
H	-5.3564690	2.9070760	-0.8024670
H	-6.9876870	1.3715780	0.2728620
C	-0.4961830	1.5134980	1.1384610
N	-0.3584820	1.5341960	-0.1632940
N	-0.1528610	2.8341370	1.7021450
C	0.0875710	2.9041630	-0.4690710
C	0.1881560	3.6229590	0.6922910
C	-0.1834850	3.2067050	3.1238180

C	0.3836190	3.4687300	-1.7066640
C	0.5901400	4.9547470	0.7083770
H	0.7700530	3.0058700	3.5657810
H	-0.4061820	4.2493310	3.2145790
H	-0.9366160	2.6361030	3.6259200
C	0.7952690	4.8181910	-1.7332520
H	0.3017120	2.8981420	-2.6081190
C	0.8984350	5.5603600	-0.5273160
H	0.6628320	5.5002990	1.6259760
H	1.0308680	5.2845020	-2.6670330
H	1.2113200	6.5831850	-0.5561520
C	2.3628470	-0.6807890	1.1324440
N	2.2534500	-0.1014630	-0.0364740
N	3.7340200	-1.1979330	1.3117300
C	3.5869030	-0.2387760	-0.6466540
C	4.4185470	-0.9032780	0.2149450
C	4.2469430	-1.9097700	2.4911780
C	4.0254100	0.2028670	-1.8919160
C	5.7461330	-1.1772080	-0.0980860
H	4.0934360	-2.9614800	2.3677230
H	5.2929280	-1.7123440	2.5999790
H	3.7278170	-1.5736260	3.3643420
C	5.3663300	-0.0554780	-2.2474450
H	3.3672840	0.7211870	-2.5575840
C	6.2256970	-0.7446360	-1.3517400
H	6.3809450	-1.6977530	0.5881690
H	5.7373520	0.2696690	-3.1969310
H	7.2411810	-0.9365540	-1.6289770
C	0.7451120	-3.5230560	-1.3781930
H	1.0258810	-2.9179490	-2.2148010
H	1.6136650	-3.7538310	-0.7974520
H	0.2986080	-4.4303120	-1.7280790

74

Mn_IV_MeTBEN_Oxo_G1_Quartet.log

74

Final Energy = -2828.1972252900

C	-0.8203980	0.4696340	2.2666570
H	-1.8436700	0.0980230	2.1713360
H	-0.6922370	0.7804370	3.3054100
N	0.1112090	-0.6097480	1.8823150
C	-0.3503600	-1.9766430	2.2107900
H	-0.1846090	-2.2128410	3.2645880
H	-1.4251100	-2.0193230	2.0405290

C	0.3895370	-2.9638870	1.3414820
H	1.4627050	-2.8895890	1.5193140
H	0.1039680	-3.9882050	1.5882350
N	0.1757720	-2.7131790	-0.1182780
C	1.4814140	-0.3414020	2.3924060
H	1.7377240	-1.0473230	3.1861820
H	1.5107480	0.6478340	2.8543930
C	-1.1278290	-3.2587460	-0.5777450
H	-1.3639660	-4.1822270	-0.0443430
H	-1.0286840	-3.5204490	-1.6333610
Mn	0.1324910	-0.5786320	-0.3670060
O	0.2709000	-0.5688030	-2.0226200
C	-2.1851330	-2.2372430	-0.4493520
N	-1.8762230	-0.9510750	-0.3715590
N	-3.5101040	-2.4456860	-0.4929970
C	-3.0784940	-0.2576950	-0.3960420
C	-4.1151180	-1.2017140	-0.4571600
C	-4.2051910	-3.7112680	-0.6102700
C	-3.3788510	1.1014140	-0.3949290
C	-5.4536690	-0.8455540	-0.4867590
H	-4.9225410	-3.8134820	0.2003450
H	-4.7300680	-3.7612510	-1.5619490
H	-3.4941890	-4.5277290	-0.5577890
C	-4.7140210	1.4617800	-0.4251470
H	-2.6098350	1.8590390	-0.4083320
C	-5.7354730	0.5078080	-0.4626410
H	-6.2378630	-1.5884390	-0.5338040
H	-4.9729230	2.5118820	-0.4315970
H	-6.7657700	0.8352470	-0.4864090
C	-0.6019690	1.5823760	1.3044380
N	-0.1476490	1.3208560	0.0802220
N	-0.8376310	2.8921950	1.4731620
C	-0.0740690	2.5222820	-0.5929810
C	-0.5151340	3.5255010	0.2798360
C	-1.3709020	3.5504540	2.6487120
C	0.3385250	2.8201840	-1.8843570
C	-0.5674680	4.8584560	-0.0984290
H	-0.7132400	4.3648110	2.9418450
H	-2.3608390	3.9476590	2.4334070
H	-1.4437170	2.8420730	3.4658060
C	0.2887150	4.1487080	-2.2628370
H	0.6748190	2.0342390	-2.5474370
C	-0.1560120	5.1475770	-1.3872290

H	-0.9107630	5.6312330	0.5751000
H	0.5992830	4.4270910	-3.2607330
H	-0.1791100	6.1736110	-1.7281960
C	2.4629540	-0.3906060	1.2839040
N	2.0719770	-0.4698170	0.0182870
N	3.7974130	-0.3165260	1.3922800
C	3.2179300	-0.4307730	-0.7598450
C	4.3152240	-0.3364420	0.1046830
C	4.5782110	-0.2110070	2.6078420
C	3.3942810	-0.4777490	-2.1360100
C	5.6199330	-0.2824570	-0.3586840
H	5.3332800	-0.9930420	2.6257600
H	5.0671410	0.7598280	2.6552750
H	3.9324150	-0.3251860	3.4710490
C	4.6955810	-0.4225200	-2.6023660
H	2.5377720	-0.5515860	-2.7914840
C	5.7872400	-0.3269200	-1.7317530
H	6.4624880	-0.2065660	0.3145890
H	4.8762470	-0.4532700	-3.6684000
H	6.7872260	-0.2857880	-2.1416910
C	1.2555290	-3.3517340	-0.9022460
H	1.1566800	-3.0479440	-1.9411160
H	2.2195160	-3.0245360	-0.5257220
H	1.1943910	-4.4380280	-0.8225420

74

Mn_IV_MeTBEN_Oxo_G1_Sextet.log

74

Final Energy = -2827.8883775300

C	-0.7648180	0.5423740	2.3699100
H	-1.7320270	0.0329940	2.3358830
H	-0.5856860	0.8456060	3.4063120
N	0.2837460	-0.4147310	1.9086470
C	0.0600330	-1.7709180	2.4952690
H	0.3277590	-1.7733450	3.5575850
H	-1.0095190	-1.9818090	2.4269890
C	0.8584700	-2.8235100	1.7420220
H	1.9259460	-2.5931220	1.7771510
H	0.7267270	-3.7985930	2.2297880
N	0.4539350	-2.8701090	0.3195950
C	1.6444260	0.1189110	2.2600670
H	2.0196650	-0.3834890	3.1562960
H	1.5563980	1.1820920	2.5031670
C	-0.8527670	-3.5383380	0.1339110

H	-1.1158620	-4.1396180	1.0126490
H	-0.7880390	-4.2342310	-0.7089120
Mn	0.1714880	-0.5690210	-0.2860140
O	0.2023930	-0.7718380	-2.1200840
C	-1.9354620	-2.5462630	-0.1582600
N	-1.6966190	-1.2429540	-0.3885630
N	-3.2476680	-2.8283250	-0.2508680
C	-2.9318600	-0.6459410	-0.6733300
C	-3.9192780	-1.6450250	-0.5781640
C	-3.8949080	-4.1271330	-0.0773030
C	-3.2765810	0.6641760	-1.0130680
C	-5.2715480	-1.3868340	-0.7924330
H	-4.6732650	-4.0419770	0.6838870
H	-4.3363540	-4.4429680	-1.0256010
H	-3.1600110	-4.8637930	0.2417540
C	-4.6279690	0.9321320	-1.2298850
H	-2.5276870	1.4367040	-1.1223390
C	-5.6093180	-0.0723230	-1.1181200
H	-6.0241220	-2.1637960	-0.7145480
H	-4.9289470	1.9402360	-1.4973440
H	-6.6503140	0.1783310	-1.2958390
C	-0.7661490	1.6976520	1.4185590
N	-0.3333590	1.5218980	0.1761730
N	-1.1863850	2.9680580	1.6470820
C	-0.4673530	2.7467290	-0.4654520
C	-1.0132020	3.6707000	0.4528280
C	-1.7449850	3.5298400	2.8730660
C	-0.1743700	3.1259530	-1.7791850
C	-1.2836340	4.9951460	0.1033100
H	-1.1564150	4.3983540	3.1774420
H	-2.7820000	3.8293650	2.7020110
H	-1.7150540	2.7823100	3.6645300
C	-0.4425740	4.4463050	-2.1342290
H	0.2382180	2.4121640	-2.4850630
C	-0.9865960	5.3640630	-1.2087650
H	-1.7047140	5.7028960	0.8096110
H	-0.2311550	4.7797070	-3.1457060
H	-1.1809790	6.3836790	-1.5272720
C	2.5814480	-0.0289670	1.1057520
N	2.1299870	-0.2821430	-0.1265810
N	3.9240190	0.1117430	1.1222170
C	3.2347670	-0.3064710	-0.9743350
C	4.3779050	-0.0547220	-0.1901490

C	4.7824750	0.4084800	2.2671120
C	3.3353180	-0.5316810	-2.3491840
C	5.6598890	-0.0102800	-0.7395640
H	5.5576050	-0.3568470	2.3440440
H	5.2431590	1.3900380	2.1333110
H	4.1888850	0.4106370	3.1798710
C	4.6128140	-0.4893190	-2.9033990
H	2.4517500	-0.7310230	-2.9466360
C	5.7541120	-0.2324870	-2.1130350
H	6.5378150	0.1872450	-0.1340400
H	4.7353800	-0.6575090	-3.9688260
H	6.7306700	-0.2070580	-2.5863340
C	1.4936090	-3.5121010	-0.5141870
H	1.1968470	-3.4477890	-1.5622520
H	2.4349000	-2.9760660	-0.3888220
H	1.6391280	-4.5640680	-0.2342050

74

Mn_IV_MeTBEN_Oxo_G2_Doublet.log

74

Final Energy = -2827.7812107400

C	-0.7952370	0.5441520	1.9994390
H	-1.8788420	0.4196500	1.8601330
H	-0.6373760	0.8422180	3.0457240
N	-0.1214840	-0.7398880	1.6874530
C	-0.8511680	-1.9619600	2.1160590
H	-0.7087170	-2.1513080	3.1904190
H	-1.9259450	-1.8124850	1.9586280
C	-0.3114310	-3.1171800	1.2969590
H	0.7661900	-3.2458700	1.4614960
H	-0.7951740	-4.0641620	1.5744410
N	-0.5228380	-2.8537110	-0.1681780
C	1.2694290	-0.7494350	2.2335890
H	1.3787940	-1.5549430	2.9728430
H	1.4448190	0.1849650	2.7857540
C	-1.9633660	-3.0692860	-0.5143660
H	-2.3851590	-3.8641370	0.1164780
H	-2.0202950	-3.4236590	-1.5504180
Mn	-0.0803660	-0.8347300	-0.4615970
O	0.1044500	-0.8805510	-2.1312230
C	-2.8068150	-1.7817770	-0.4642060
N	-2.3239110	-0.5653330	-0.4961590
N	-4.2796790	-1.7365640	-0.3729720
C	-3.5079810	0.3076340	-0.4242020

C	-4.6395560	-0.4604690	-0.3527770
C	-5.1826720	-2.8950770	-0.3150720
C	-3.5667840	1.6985180	-0.4228610
C	-5.9116020	0.0975050	-0.2748460
H	-5.3393450	-3.1744990	0.7058480
H	-6.1202400	-2.6392350	-0.7627440
H	-4.7457100	-3.7147690	-0.8461740
C	-4.8391530	2.3036800	-0.3449860
H	-2.6764230	2.2892800	-0.4790330
C	-6.0101520	1.5042820	-0.2710690
H	-6.7844010	-0.5190030	-0.2197170
H	-4.9218620	3.3704730	-0.3416250
H	-6.9711920	1.9710210	-0.2122670
C	-0.2680110	1.6132790	1.0244530
N	-0.1412090	1.4538370	-0.2687810
N	0.1692440	2.9697520	1.4097920
C	0.3938800	2.7381930	-0.7518770
C	0.5536570	3.5943180	0.3050370
C	0.1770370	3.5265220	2.7702500
C	0.7161280	3.1142160	-2.0529450
C	1.0451540	4.8860850	0.1460420
H	1.1187880	3.3205250	3.2345450
H	0.0263960	4.5848270	2.7233850
H	-0.6084010	3.0794800	3.3430860
C	1.2180740	4.4172120	-2.2565670
H	0.5874280	2.4374340	-2.8716640
C	1.3824200	5.3021150	-1.1585340
H	1.1631110	5.5408510	0.9840560
H	1.4761480	4.7399850	-3.2435400
H	1.7636630	6.2885240	-1.3214790
C	2.3501390	-0.8606700	1.1421250
N	2.1158630	-1.0699210	-0.1288130
N	3.8037470	-0.7471130	1.3741280
C	3.4491000	-1.0955130	-0.7541900
C	4.4078810	-0.8961060	0.2031620
C	4.4614820	-0.5117660	2.6675320
C	3.7805060	-1.2850470	-2.0929440
C	5.7654210	-0.8705270	-0.1000550
H	4.6742450	-1.4502090	3.1354590
H	5.3744410	0.0239140	2.5111570
H	3.8145200	0.0623060	3.2974390
C	5.1476500	-1.2660370	-2.4418930
H	3.0234270	-1.4400500	-2.8330150

C	6.1389420	-1.0590450	-1.4467890
H	6.5002070	-0.7133590	0.6617110
H	5.4387230	-1.4083480	-3.4616600
H	7.1734660	-1.0460340	-1.7197210
C	0.3157640	-3.7708300	-0.9533780
H	0.5613850	-3.3158720	-1.8901730
H	1.2147710	-3.9828880	-0.4132700
H	-0.2184520	-4.6812900	-1.1282320

74

Mn_IV_MeTBEN_Oxo_G2_Quartet.log

74

Final Energy = -2828.1972255500

C	-0.8204640	0.4695170	2.2666090
H	-1.8436820	0.0977590	2.1713150
H	-0.6923140	0.7803470	3.3053600
N	0.1112920	-0.6097290	1.8822400
C	-0.3501260	-1.9767060	2.2106580
H	-0.1843450	-2.2129080	3.2644490
H	-1.4248680	-2.0195020	2.0403960
C	0.3898880	-2.9638490	1.3413180
H	1.4630500	-2.8894660	1.5191750
H	0.1044110	-3.9882020	1.5880220
N	0.1761360	-2.7131110	-0.1184340
C	1.4814290	-0.3412080	2.3923870
H	1.7377880	-1.0470200	3.1862420
H	1.5105950	0.6480860	2.8542730
C	-1.1273910	-3.2588160	-0.5779620
H	-1.3633920	-4.1823570	-0.0446010
H	-1.0281880	-3.5204580	-1.6335780
Mn	0.1326320	-0.5785760	-0.3670790
O	0.2711100	-0.5687840	-2.0226850
C	-2.1848160	-2.2374600	-0.4494970
N	-1.8760340	-0.9512610	-0.3716630
N	-3.5097620	-2.4460360	-0.4930220
C	-3.0783760	-0.2580040	-0.3959540
C	-4.1149040	-1.2021290	-0.4569970
C	-4.2047650	-3.7116240	-0.6105330
C	-3.3788890	1.1010700	-0.3948030
C	-5.4535010	-0.8461140	-0.4865730
H	-4.9215890	-3.8143470	0.2004870
H	-4.7302560	-3.7611230	-1.5618970
H	-3.4936360	-4.5280290	-0.5589380
C	-4.7140950	1.4612890	-0.4249350

H	-2.6099710	1.8587890	-0.4082580
C	-5.7354490	0.5072130	-0.4624030
H	-6.2376030	-1.5890980	-0.5335930
H	-4.9731160	2.5113630	-0.4313260
H	-6.7657790	0.8345530	-0.4861160
C	-0.6022320	1.5823230	1.3044170
N	-0.1477770	1.3208830	0.0802340
N	-0.8383310	2.8920820	1.4730540
C	-0.0744100	2.5223030	-0.5929940
C	-0.5158040	3.5254220	0.2797550
C	-1.3717350	3.5503570	2.6485310
C	0.3382510	2.8202940	-1.8843230
C	-0.5683580	4.8583570	-0.0984740
H	-0.7128540	4.3631870	2.9432150
H	-2.3605810	3.9497360	2.4322560
H	-1.4471360	2.8414100	3.4648810
C	0.2881670	4.1488090	-2.2628120
H	0.6747820	2.0344210	-2.5473710
C	-0.1568580	5.1475780	-1.3872400
H	-0.9118430	5.6310320	0.5750730
H	0.5987620	4.4272610	-3.2606790
H	-0.1801400	6.1736150	-1.7281880
C	2.4630120	-0.3904270	1.2839510
N	2.0720900	-0.4695670	0.0183170
N	3.7974680	-0.3164460	1.3924250
C	3.2180820	-0.4304120	-0.7597490
C	4.3153340	-0.3361080	0.1048400
C	4.5780710	-0.2108770	2.6081130
C	3.3944910	-0.4772810	-2.1359110
C	5.6200550	-0.2819560	-0.3584850
H	5.3350400	-0.9910820	2.6246580
H	5.0646450	0.7610710	2.6571600
H	3.9326460	-0.3282070	3.4711780
C	4.6957990	-0.4219260	-2.6022120
H	2.5380110	-0.5511410	-2.7914220
C	5.7874130	-0.3262840	-1.7315500
H	6.4625880	-0.2058910	0.3147930
H	4.8765090	-0.4525660	-3.6682420
H	6.7874100	-0.2849790	-2.1414460
C	1.2559770	-3.3515700	-0.9023670
H	1.1569810	-3.0480010	-1.9412850
H	2.2199220	-3.0240850	-0.5259880
H	1.1951000	-4.4378690	-0.8224420

74
Mn_IV_MeTBEN_Oxo_G2_Sextet.log
74
Final Energy = -2828.1502375000

C	-0.6885700	0.6368560	2.3748040
H	-1.6542650	0.1267930	2.3871890
H	-0.4897410	0.9610050	3.3990150
N	0.3364580	-0.3312970	1.9120670
C	0.1267380	-1.6636760	2.5395620
H	0.4106060	-1.6339490	3.5938650
H	-0.9446090	-1.8692410	2.5045220
C	0.8910000	-2.7359320	1.8070730
H	1.9567060	-2.5025790	1.7794410
H	0.7982680	-3.6851530	2.3446000
N	0.4228110	-2.8469070	0.4164960
C	1.7048940	0.2008690	2.1957410
H	2.0986790	-0.2521630	3.1082670
H	1.6301560	1.2726520	2.3936070
C	-0.8944380	-3.4947670	0.3221080
H	-1.1589950	-3.9927680	1.2604940
H	-0.8609170	-4.2885650	-0.4273590
Mn	0.1553090	-0.5464700	-0.2793250
O	0.1405650	-0.8562770	-2.0937130
C	-1.9605930	-2.5322580	-0.0551820
N	-1.7313490	-1.2462510	-0.3352750
N	-3.2579860	-2.8398670	-0.1696260
C	-2.9581900	-0.6921980	-0.6779820
C	-3.9302180	-1.6906760	-0.5634580
C	-3.8792590	-4.1272720	0.0661080
C	-3.3060790	0.5875590	-1.0878540
C	-5.2692120	-1.4609060	-0.8226710
H	-4.5776660	-4.0554310	0.8969110
H	-4.4156850	-4.4418760	-0.8259500
H	-3.1212450	-4.8654560	0.3006640
C	-4.6445340	0.8252750	-1.3523100
H	-2.5617520	1.3602300	-1.2171880
C	-5.6085580	-0.1766580	-1.2184280
H	-6.0120120	-2.2400460	-0.7241280
H	-4.9500710	1.8105610	-1.6769020
H	-6.6430510	0.0520860	-1.4347910
C	-0.7181480	1.7644430	1.4089910
N	-0.3237890	1.5739240	0.1630440
N	-1.1350390	3.0260840	1.6419230

C	-0.4843810	2.7886070	-0.4726190
C	-1.0013630	3.7128310	0.4464020
C	-1.6384640	3.5895870	2.8766330
C	-0.2298590	3.1622200	-1.7871110
C	-1.2791960	5.0267550	0.1013340
H	-1.0319020	4.4437220	3.1694660
H	-2.6692680	3.9127640	2.7467380
H	-1.6022550	2.8449750	3.6636690
C	-0.5057810	4.4713480	-2.1372820
H	0.1664260	2.4508200	-2.5001060
C	-1.0200190	5.3863240	-1.2099380
H	-1.6776410	5.7345630	0.8152460
H	-0.3212670	4.7996800	-3.1512900
H	-1.2210970	6.4006530	-1.5272340
C	2.6109020	-0.0062160	1.0407210
N	2.1418950	-0.2946830	-0.1685590
N	3.9482510	0.1137700	1.0430520
C	3.2334850	-0.3661040	-1.0140960
C	4.3811390	-0.1063470	-0.2564790
C	4.8113260	0.4326560	2.1625800
C	3.3195110	-0.6393200	-2.3726320
C	5.6496340	-0.1012810	-0.8141380
H	5.5668850	-0.3413530	2.2739400
H	5.3007500	1.3891660	1.9924260
H	4.2287310	0.4918420	3.0745820
C	4.5836250	-0.6360780	-2.9333810
H	2.4309670	-0.8436190	-2.9555370
C	5.7263600	-0.3710550	-2.1691280
H	6.5319430	0.1035000	-0.2238930
H	4.6945620	-0.8422810	-3.9892400
H	6.6947830	-0.3774580	-2.6505000
C	1.4097100	-3.5239860	-0.4339810
H	1.0766990	-3.4892880	-1.4686230
H	2.3618940	-3.0032760	-0.3640900
H	1.5606030	-4.5655330	-0.1371720

74
Mn_IV_MeTBEN_Oxo_G3_Doublet.log
74
Final Energy = -2827.7373099600

C	-0.9273830	0.3269290	1.9544350
H	-1.9940570	0.1466330	1.7574350
H	-0.8297850	0.5387040	3.0287260
N	-0.1571980	-0.8780500	1.5608920

C	-0.8185100	-2.1779100	1.8483530
H	-0.7044600	-2.4540780	2.9071820
H	-1.8941760	-2.0837090	1.6577200
C	-0.1713960	-3.2175920	0.9555610
H	0.9054340	-3.2915970	1.1552130
H	-0.6003300	-4.2147020	1.1275110
N	-0.3443750	-2.8368510	-0.4886850
C	1.2094740	-0.8481310	2.1644700
H	1.3448120	-1.7085110	2.8343420
H	1.3003620	0.0418370	2.8035680
C	-1.7529100	-3.1117430	-0.9151840
H	-2.1435250	-3.9847590	-0.3715270
H	-1.7462620	-3.3761540	-1.9769770
Mn	-0.0282990	-0.7755140	-0.5843300
O	0.2223760	-0.6584050	-2.2418440
C	-2.6825860	-1.8904880	-0.7893340
N	-2.3857970	-0.6766950	-1.1799460
N	-4.0404680	-1.9152020	-0.2103320
C	-3.5782750	0.1226760	-0.8508970
C	-4.5247370	-0.6824740	-0.2751450
C	-4.7299790	-3.0919440	0.3380780
C	-3.7999260	1.4819100	-1.0542890
C	-5.7600300	-0.1956740	0.1402910
H	-4.5124390	-3.1783780	1.3821590
H	-5.7855840	-2.9825920	0.2015510
H	-4.3938030	-3.9714010	-0.1702920
C	-5.0412030	2.0149030	-0.6464180
H	-3.0535760	2.1021050	-1.5050880
C	-6.0200940	1.1772630	-0.0498710
H	-6.4871500	-0.8402560	0.5882980
H	-5.2457380	3.0556610	-0.7874450
H	-6.9588140	1.5892900	0.2566260
C	-0.4368830	1.5112300	1.1009490
N	-0.2809900	1.4872340	-0.1986960
N	-0.0728530	2.8417180	1.6272390
C	0.1986850	2.8369430	-0.5414320
C	0.2988910	3.5903430	0.5978200
C	-0.1148500	3.2602730	3.0357660
C	0.5237440	3.3551240	-1.7919690
C	0.7292880	4.9131610	0.5773330
H	0.8279990	3.0535990	3.4975260
H	-0.3161960	4.3097510	3.0901350
H	-0.8869210	2.7221310	3.5448920

C	0.9647690	4.6940280	-1.8555260
H	0.4418600	2.7577660	-2.6759140
C	1.0674490	5.4721600	-0.6724340
H	0.8011980	5.4862140	1.4780760
H	1.2231230	5.1250520	-2.8001810
H	1.4027180	6.4866860	-0.7292740
C	2.3358420	-0.7911070	1.1158310
N	2.1621600	-0.5658130	-0.1620710
N	3.7713690	-0.9824080	1.4028200
C	3.5180060	-0.6091400	-0.7357240
C	4.4272510	-0.8621180	0.2566580
C	4.3653530	-1.2563190	2.7192760
C	3.9103620	-0.4333690	-2.0598000
C	5.7922150	-0.9588160	0.0055950
H	4.4096400	-2.3136180	2.8775710
H	5.3538500	-0.8484040	2.7564070
H	3.7649240	-0.8063130	3.4820860
C	5.2870200	-0.5238820	-2.3561590
H	3.1916550	-0.2361170	-2.8275570
C	6.2268510	-0.7862690	-1.3248440
H	6.4877250	-1.1571170	0.7941650
H	5.6243290	-0.3941050	-3.3632740
H	7.2689890	-0.8533590	-1.5579670
C	0.8573740	-3.2244850	-1.2413080
H	1.1208610	-2.4410030	-1.9207420
H	1.6655530	-3.3931000	-0.5606350
H	0.6604480	-4.1215060	-1.7903730

74

Mn_IV_MeTBEN_Oxo_G3_Quartet.log

74

Final Energy = -2827.7669035800

C	-0.9273830	0.3269290	1.9544350
H	-1.9940570	0.1466330	1.7574350
H	-0.8297850	0.5387040	3.0287260
N	-0.1571980	-0.8780500	1.5608920
C	-0.8185100	-2.1779100	1.8483530
H	-0.7044600	-2.4540780	2.9071820
H	-1.8941760	-2.0837090	1.6577200
C	-0.1713960	-3.2175920	0.9555610
H	0.9054340	-3.2915970	1.1552130
H	-0.6003300	-4.2147020	1.1275110
N	-0.3443750	-2.8368510	-0.4886850
C	1.2094740	-0.8481310	2.1644700

H	1.3448120	-1.7085110	2.8343420
H	1.3003620	0.0418370	2.8035680
C	-1.7529100	-3.1117430	-0.9151840
H	-2.1435250	-3.9847590	-0.3715270
H	-1.7462620	-3.3761540	-1.9769770
Mn	-0.0282990	-0.7755140	-0.5843300
O	0.2223760	-0.6584050	-2.2418440
C	-2.6825860	-1.8904880	-0.7893340
N	-2.3857970	-0.6766950	-1.1799460
N	-4.0404680	-1.9152020	-0.2103320
C	-3.5782750	0.1226760	-0.8508970
C	-4.5247370	-0.6824740	-0.2751450
C	-4.7299790	-3.0919440	0.3380780
C	-3.7999260	1.4819100	-1.0542890
C	-5.7600300	-0.1956740	0.1402910
H	-4.5124390	-3.1783780	1.3821590
H	-5.7855840	-2.9825920	0.2015510
H	-4.3938030	-3.9714010	-0.1702920
C	-5.0412030	2.0149030	-0.6464180
H	-3.0535760	2.1021050	-1.5050880
C	-6.0200940	1.1772630	-0.0498710
H	-6.4871500	-0.8402560	0.5882980
H	-5.2457380	3.0556610	-0.7874450
H	-6.9588140	1.5892900	0.2566260
C	-0.4368830	1.5112300	1.1009490
N	-0.2809900	1.4872340	-0.1986960
N	-0.0728530	2.8417180	1.6272390
C	0.1986850	2.8369430	-0.5414320
C	0.2988910	3.5903430	0.5978200
C	-0.1148500	3.2602730	3.0357660
C	0.5237440	3.3551240	-1.7919690
C	0.7292880	4.9131610	0.5773330
H	0.8279990	3.0535990	3.4975260
H	-0.3161960	4.3097510	3.0901350
H	-0.8869210	2.7221310	3.5448920
C	0.9647690	4.6940280	-1.8555260
H	0.4418600	2.7577660	-2.6759140
C	1.0674490	5.4721600	-0.6724340
H	0.8011980	5.4862140	1.4780760
H	1.2231230	5.1250520	-2.8001810
H	1.4027180	6.4866860	-0.7292740
C	2.3358420	-0.7911070	1.1158310
N	2.1621600	-0.5658130	-0.1620710

N	3.7713690	-0.9824080	1.4028200
C	3.5180060	-0.6091400	-0.7357240
C	4.4272510	-0.8621180	0.2566580
C	4.3653530	-1.2563190	2.7192760
C	3.9103620	-0.4333690	-2.0598000
C	5.7922150	-0.9588160	0.0055950
H	4.4096400	-2.3136180	2.8775710
H	5.3538500	-0.8484040	2.7564070
H	3.7649240	-0.8063130	3.4820860
C	5.2870200	-0.5238820	-2.3561590
H	3.1916550	-0.2361170	-2.8275570
C	6.2268510	-0.7862690	-1.3248440
H	6.4877250	-1.1571170	0.7941650
H	5.6243290	-0.3941050	-3.3632740
H	7.2689890	-0.8533590	-1.5579670
C	0.8573740	-3.2244850	-1.2413080
H	1.1208610	-2.4410030	-1.9207420
H	1.6655530	-3.3931000	-0.5606350
H	0.6604480	-4.1215060	-1.7903730

74

Mn_IV_MeTBEN_Oxo_G3_Sextet.log

74

Final Energy = -2828.1502394000

C	-0.6863710	0.6383030	2.3763780
H	-1.6522110	0.1285490	2.3893080
H	-0.4871990	0.9629340	3.4004050
N	0.3380820	-0.3304740	1.9137720
C	0.1288330	-1.6621360	2.5428600
H	0.4149000	-1.6317180	3.5965580
H	-0.9426910	-1.8670880	2.5102040
C	0.8911000	-2.7355110	1.8098740
H	1.9569360	-2.5029910	1.7805490
H	0.7984460	-3.6843140	2.3481670
N	0.4205910	-2.8467850	0.4202320
C	1.7068680	0.2018020	2.1952730
H	2.1017010	-0.2504080	3.1077610
H	1.6325730	1.2737800	2.3922660
C	-0.8973840	-3.4935050	0.3282550
H	-1.1626850	-3.9868770	1.2688850
H	-0.8648560	-4.2908970	-0.4174850
Mn	0.1546570	-0.5466110	-0.2780690
O	0.1388720	-0.8590140	-2.0921800
C	-1.9623750	-2.5312690	-0.0529290

N	-1.7323180	-1.2454410	-0.3332710
N	-3.2595720	-2.8387280	-0.1700220
C	-2.9584590	-0.6911390	-0.6779570
C	-3.9309490	-1.6893020	-0.5646020
C	-3.8818610	-4.1252430	0.0683020
C	-3.3054010	0.5885470	-1.0889120
C	-5.2694760	-1.4592350	-0.8259160
H	-4.5595630	-4.0590840	0.9166830
H	-4.4415240	-4.4271390	-0.8136810
H	-3.1222410	-4.8699910	0.2754720
C	-4.6433950	0.8265820	-1.3553660
H	-2.5606630	1.3609570	-1.2174990
C	-5.6079230	-0.1750240	-1.2225670
H	-6.0125020	-2.2382350	-0.7280470
H	-4.9481560	1.8118790	-1.6806570
H	-6.6420440	0.0539790	-1.4404080
C	-0.7160280	1.7654400	1.4100750
N	-0.3232630	1.5739970	0.1637460
N	-1.1311680	3.0276840	1.6429380
C	-0.4833000	2.7885780	-0.4722840
C	-0.9983820	3.7136950	0.4469030
C	-1.6340900	3.5919710	2.8774990
C	-0.2297480	3.1613660	-1.7872010
C	-1.2753400	5.0277130	0.1015310
H	-1.0317440	4.4507430	3.1652900
H	-2.6673300	3.9084300	2.7502340
H	-1.5902500	2.8503080	3.6669240
C	-0.5047600	4.4706090	-2.1376650
H	0.1650850	2.4492260	-2.5002640
C	-1.0171680	5.3864660	-1.2101750
H	-1.6725750	5.7361650	0.8154750
H	-0.3209950	4.7983440	-3.1520000
H	-1.2176860	6.4008300	-1.5277110
C	2.6115050	-0.0064810	1.0393950
N	2.1411040	-0.2957480	-0.1691590
N	3.9489170	0.1128630	1.0401990
C	3.2317770	-0.3684320	-1.0157850
C	4.3803420	-0.1086160	-0.2595600
C	4.8134030	0.4322130	2.1584930
C	3.3161970	-0.6427780	-2.3741990
C	5.6482360	-0.1045840	-0.8186150
H	5.5684540	-0.3422860	2.2699260
H	5.3034230	1.3881590	1.9868750

H 4.2317910 0.4928400 3.0710230
C 4.5797020 -0.6405540 -2.9363210
H 2.4268860 -0.8470940 -2.9559330
C 5.7233680 -0.3754590 -2.1734780
H 6.5313030 0.1002470 -0.2295270
H 4.6894430 -0.8476380 -3.9921320
H 6.6912730 -0.3826760 -2.6558790
C 1.4054140 -3.5245150 -0.4321290
H 1.0709030 -3.4886590 -1.4662500
H 2.3583430 -3.0050450 -0.3632710
H 1.5554200 -4.5664390 -0.1362120
90
TS_MnMeTBEN_Thioanisole_Quartet.log
90
Final Energy = -3495.5853819100
C -0.0649580 1.5449790 -2.4986620
H 0.8829780 1.0593400 -2.7848170
H -0.3541710 2.1749020 -3.3581070
N -1.0645410 0.4987030 -2.2255180
C -0.9475090 -0.6300960 -3.1726660
H -1.3528900 -0.3553430 -4.1609510
H 0.1233350 -0.8270170 -3.3285690
C -1.6472020 -1.8649320 -2.6568590
H -2.7162620 -1.6562660 -2.4961090
H -1.6099890 -2.6537760 -3.4301630
N -1.0880210 -2.3369620 -1.3819820
C -2.4307990 1.0752910 -2.2161140
H -2.9256530 0.9232990 -3.1899900
H -2.3599820 2.1679940 -2.0889460
C 0.2174830 -2.9869000 -1.5491250
H 0.2835470 -3.5250310 -2.5122470
H 0.3251540 -3.7671320 -0.7768030
Mn -0.6567500 -0.3111920 -0.1411890
O -0.6297210 -0.8748280 1.6532710
C 1.3364280 -2.0159400 -1.4057440
N 1.2061970 -0.8550810 -0.7656030
N 2.5933570 -2.1988320 -1.8810220
C 2.4460940 -0.2318580 -0.8350370
C 3.3236180 -1.0700940 -1.5521780
C 3.1150720 -3.3356100 -2.6069060
C 2.9005960 0.9936720 -0.3387610
C 4.6477170 -0.7235700 -1.8156680
H 3.9976190 -3.7355360 -2.0925910

H	2.3633410	-4.1264320	-2.6631920
H	3.4002950	-3.0441500	-3.6256700
C	4.2161030	1.3536920	-0.6114150
H	2.2645300	1.6293800	0.2772220
C	5.0752040	0.5124790	-1.3393820
H	5.3157860	-1.3857720	-2.3688250
H	4.5944670	2.3061480	-0.2332160
H	6.1027000	0.8299550	-1.5274900
C	0.1415530	2.3449830	-1.2600300
N	-0.2495190	1.8843440	-0.0852330
N	0.7808660	3.5401580	-1.1731620
C	0.1610280	2.8167440	0.8441150
C	0.8193870	3.8699250	0.1734650
C	1.3924010	4.3010420	-2.2388420
C	0.0495360	2.8181310	2.2381120
C	1.3793050	4.9516000	0.8530510
H	1.0137640	5.3302680	-2.2372000
H	2.4835670	4.3241170	-2.1172040
H	1.1591090	3.8457100	-3.2051420
C	0.6063970	3.8946210	2.9206340
H	-0.4500620	1.9963660	2.7559040
C	1.2571550	4.9419080	2.2402360
H	1.8915790	5.7608420	0.3298230
H	0.5415160	3.9308240	4.0100130
H	1.6814980	5.7667980	2.8160510
C	-3.2399910	0.5300000	-1.0978270
N	-2.6803500	-0.0586300	-0.0465590
N	-4.5852720	0.6155370	-0.9856280
C	-3.7153300	-0.3800270	0.8176180
C	-4.9256780	0.0423150	0.2309260
C	-5.5218820	1.2120220	-1.9121190
C	-3.7195850	-1.0120210	2.0652100
C	-6.1630690	-0.1394260	0.8473100
H	-6.3016300	0.4882680	-2.1773350
H	-5.9937840	2.0953960	-1.4640110
H	-5.0054340	1.5180270	-2.8251480
C	-4.9513750	-1.1965360	2.6841540
H	-2.7842700	-1.3305030	2.5223100
C	-6.1520550	-0.7695000	2.0879180
H	-7.0922160	0.1953080	0.3832710
H	-4.9889170	-1.6836380	3.6606760
H	-7.0964510	-0.9337530	2.6104070
C	-2.0313830	-3.2086160	-0.6823810

H	-1.6615210	-3.4301770	0.3266240
H	-2.9972230	-2.6993630	-0.5804630
H	-2.1996400	-4.1609810	-1.2137390
C	2.1199270	-1.4245830	2.4411430
C	2.8601300	-0.2896360	2.8223970
C	4.2114540	-0.2105180	2.5087000
C	4.8334250	-1.2440950	1.8034510
C	4.0935710	-2.3664730	1.4177840
C	2.7428260	-2.4668820	1.7287540
H	2.3720000	0.5177260	3.3753280
H	4.7839840	0.6685030	2.8126090
H	4.5779330	-3.1772130	0.8676420
H	2.1878910	-3.3567310	1.4266740
S	0.4071480	-1.3793800	2.8547070
C	-0.0123580	-3.1193000	2.8392000
H	0.6105560	-3.6559710	3.5642320
H	-1.0654550	-3.1924610	3.1302160
H	0.1188770	-3.5336510	1.8329880
H	5.8932520	-1.1751860	1.5503750

TS_MnMeTBEN_Thioanisole_Quartet
90
2 4

C	-0.06495800	1.54497900	-2.49866200
H	0.88297800	1.05934000	-2.78481700
H	-0.35417100	2.17490200	-3.35810700
N	-1.06454100	0.49870300	-2.22551800
C	-0.94750900	-0.63009600	-3.17266600
H	-1.35289000	-0.35534300	-4.16095100
H	0.12333500	-0.82701700	-3.32856900
C	-1.64720200	-1.86493200	-2.65685900
H	-2.71626200	-1.65626600	-2.49610900
H	-1.60998900	-2.65377600	-3.43016300
N	-1.08802100	-2.33696200	-1.38198200
C	-2.43079900	1.07529100	-2.21611400
H	-2.92565300	0.92329900	-3.18999000
H	-2.35998200	2.16799400	-2.08894600
C	0.21748300	-2.98690000	-1.54912500
H	0.28354700	-3.52503100	-2.51224700
H	0.32515400	-3.76713200	-0.77680300
Mn	-0.65675000	-0.31119200	-0.14118900
O	-0.62972100	-0.87482800	1.65327100
C	1.33642800	-2.01594000	-1.40574400
N	1.20619700	-0.85508100	-0.76560300

N	2.59335700	-2.19883200	-1.88102200
C	2.44609400	-0.23185800	-0.83503700
C	3.32361800	-1.07009400	-1.55217800
C	3.11507200	-3.33561000	-2.60690600
C	2.90059600	0.99367200	-0.33876100
C	4.64771700	-0.72357000	-1.81566800
H	3.99761900	-3.73553600	-2.09259100
H	2.36334100	-4.12643200	-2.66319200
H	3.40029500	-3.04415000	-3.62567000
C	4.21610300	1.35369200	-0.61141500
H	2.26453000	1.62938000	0.27722200
C	5.07520400	0.51247900	-1.33938200
H	5.31578600	-1.38577200	-2.36882500
H	4.59446700	2.30614800	-0.23321600
H	6.10270000	0.82995500	-1.52749000
C	0.14155300	2.34498300	-1.26003000
N	-0.24951900	1.88434400	-0.08523300
N	0.78086600	3.54015800	-1.17316200
C	0.16102800	2.81674400	0.84411500
C	0.81938700	3.86992500	0.17346500
C	1.39240100	4.30104200	-2.23884200
C	0.04953600	2.81813100	2.23811200
C	1.37930500	4.95160000	0.85305100
H	1.01376400	5.33026800	-2.23720000
H	2.48356700	4.32411700	-2.11720400
H	1.15910900	3.84571000	-3.20514200
C	0.60639700	3.89462100	2.92063400
H	-0.45006200	1.99636600	2.75590400
C	1.25715500	4.94190800	2.24023600
H	1.89157900	5.76084200	0.32982300
H	0.54151600	3.93082400	4.01001300
H	1.68149800	5.76679800	2.81605100
C	-3.23999100	0.53000000	-1.09782700
N	-2.68035000	-0.05863000	-0.04655900
N	-4.58527200	0.61553700	-0.98562800
C	-3.71533000	-0.38002700	0.81761800
C	-4.92567800	0.04231500	0.23092600
C	-5.52188200	1.21202200	-1.91211900
C	-3.71958500	-1.01202100	2.06521000
C	-6.16306900	-0.13942600	0.84731000
H	-6.30163000	0.48826800	-2.17733500
H	-5.99378400	2.09539600	-1.46401100
H	-5.00543400	1.51802700	-2.82514800

C	-4.95137500	-1.19653600	2.68415400
H	-2.78427000	-1.33050300	2.52231000
C	-6.15205500	-0.76950000	2.08791800
H	-7.09221600	0.19530800	0.38327100
H	-4.98891700	-1.68363800	3.66067600
H	-7.09645100	-0.93375300	2.61040700
C	-2.03138300	-3.20861600	-0.68238100
H	-1.66152100	-3.43017700	0.32662400
H	-2.99722300	-2.69936300	-0.58046300
H	-2.19964000	-4.16098100	-1.21373900
C	2.11992700	-1.42458300	2.44114300
C	2.86013000	-0.28963600	2.82239700
C	4.21145400	-0.21051800	2.50870000
C	4.83342500	-1.24409500	1.80345100
C	4.09357100	-2.36647300	1.41778400
C	2.74282600	-2.46688200	1.72875400
H	2.37200000	0.51772600	3.37532800
H	4.78398400	0.66850300	2.81260900
H	4.57793300	-3.17721300	0.86764200
H	2.18789100	-3.35673100	1.42667400
S	0.40714800	-1.37938000	2.85470700
C	-0.01235800	-3.11930000	2.83920000
H	0.61055600	-3.65597100	3.56423200
H	-1.06545500	-3.19246100	3.13021600
H	0.11887700	-3.53365100	1.83298800
H	5.89325200	-1.17518600	1.55037500

References:

1. Gupta, S.; Arora, P.; Kumar, R.; Awasthi, A.; Chandra, B.; Eerlapally, R.; Xiong, J.; Guo, Y.; Que, L.; Draksharapu, A. Formation of a Reactive [Mn(III)−O−Ce(IV)] Species and Its Facile Equilibrium with Related Mn(IV)(OX) (X = Sc or H) Complexes. *Angew. Chem. Int. Ed.* **2024**, *63*, e202316378.

2. Wu, X.; Seo, M. S.; Davis, K. M.; Lee, Y. M.; Chen, J.; Cho, K. B.; Push-kar, Y. N.; Nam, W., A highly reactive mononuclear non-heme manganese (IV)-oxo complex that can activate the strong C-H bonds of alkanes. *J. Am. Chem. Soc.* **2011**, *133* (50), 20088-91.
